# Metalloprotease SPRTN/DVC1 Orchestrates Replication-Coupled DNA-Protein Crosslink Repair

**DOI:** 10.1016/j.molcel.2016.09.032

**Published:** 2016-11-17

**Authors:** Bruno Vaz, Marta Popovic, Joseph A. Newman, John Fielden, Hazel Aitkenhead, Swagata Halder, Abhay Narayan Singh, Iolanda Vendrell, Roman Fischer, Ignacio Torrecilla, Neele Drobnitzky, Raimundo Freire, David J. Amor, Paul J. Lockhart, Benedikt M. Kessler, Gillies W. McKenna, Opher Gileadi, Kristijan Ramadan

**Affiliations:** 1 Cancer Research UK and Medical Research Council Oxford Institute for Radiation Oncology, Department of Oncology, University of Oxford, Oxford OX3 7DQ, UK; 2 Structural Genomics Consortium, University of Oxford, Oxford OX3 7DQ, UK; 3 TDI Mass Spectrometry Laboratory, Target Discovery Institute, Nuffield Department of Medicine, University of Oxford, Oxford OX3 7FZ, UK; 4 Unidad de Investigación, Hospital Universitario de Canarias, Instituto de Tecnologías Biomédicas, Ofra s/n, 38320 La Laguna, Tenerife, Spain; 5 Bruce Lefroy Centre for Genetic Health Research, Murdoch Childrens Research Institute, Royal Children’s Hospital, Parkville, VIC 3052, Australia; 6 Department of Paediatrics, The University of Melbourne, Parkville, VIC 3052, Australia

**Keywords:** SPARTAN/DVC1, DNA-dependent metalloprotease, DNA-protein crosslink repair, DNA replication, Ruijs-Aalfs/SPARTAN syndrome, cancer, aging

## Abstract

The cytotoxicity of DNA-protein crosslinks (DPCs) is largely ascribed to their ability to block the progression of DNA replication. DPCs frequently occur in cells, either as a consequence of metabolism or exogenous agents, but the mechanism of DPC repair is not completely understood. Here, we characterize SPRTN as a specialized DNA-dependent and DNA replication-coupled metalloprotease for DPC repair. SPRTN cleaves various DNA binding substrates during S-phase progression and thus protects proliferative cells from DPC toxicity. Ruijs-Aalfs syndrome (RJALS) patient cells with monogenic and biallelic mutations in *SPRTN* are hypersensitive to DPC-inducing agents due to a defect in DNA replication fork progression and the inability to eliminate DPCs. We propose that SPRTN protease represents a specialized DNA replication-coupled DPC repair pathway essential for DNA replication progression and genome stability. Defective SPRTN-dependent clearance of DPCs is the molecular mechanism underlying RJALS, and DPCs are contributing to accelerated aging and cancer.

## Highlights

• DNA-protein crosslinks (DPCs) stall DNA replication and induce genomic instability
• SPARTAN (SPRTN) is a DNA replication-coupled metalloprotease which proteolyses DPCs
• SPRTN metalloprotease is a fundamental enzyme in DPC repair pathway
• Ruijs-Aalfs syndrome is caused by a defect in DPC repair due to mutations in *SPRTN*

## Graphical Abstract

**Figure:**
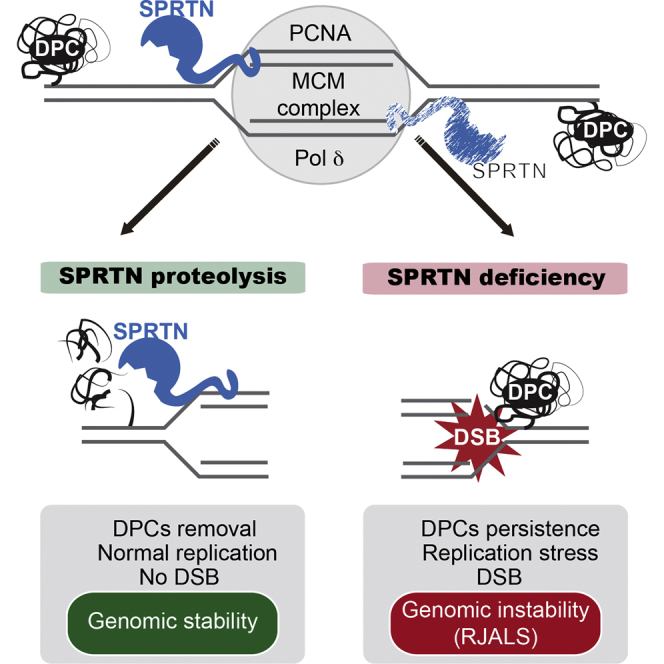

## Teaser

Monogenic mutations in *SPRTN* cause genomic instability, premature aging, and hepatocellular carcinoma. The molecular mechanism of how SPRTN protects genome stability and prevents accelerated aging and cancer is not clear. Vaz, Popovic, et al. show that SPRTN is a DNA replication-coupled metalloprotease for DNA-protein crosslink repair in proliferative human cells.

## Introduction

Numerous endogenous and exogenous factors constantly attack the genome causing a variety of chemically distinct DNA lesions (Lindahl, 1993). If not repaired, such DNA lesions lead to genomic instability and cell death (Jackson and Bartek, 2009). Cells have evolved multiple DNA repair pathways, specialized for distinct types of DNA lesions (Friedberg et al., 2006, Lindahl and Wood, 1999). DNA-protein crosslinks (DPCs) represent a so far under investigated type of DNA lesion caused by the covalent attachment of proteins to nucleobases, sugar, or broken phosphodiester bonds on the DNA backbone (Ashour et al., 2015, Tretyakova et al., 2015). Very little is known about how cells remove DPCs and repair DPC-induced DNA lesions (Barker et al., 2005, Connelly and Leach, 2004, Stingele and Jentsch, 2015, Tretyakova et al., 2015). DPCs are induced by chemical reactions catalyzed by products of cellular metabolism like aldehydes or by exogenous sources including UV-light and ionizing radiation (Ide et al., 2011, Shi et al., 2004). Virtually any protein in close vicinity to DNA could form a non-enzymatic DPC in the presence of a crosslinking compound such as formaldehyde (FA) (Shoulkamy et al., 2012). DPCs are also induced enzymatically when certain DNA-binding enzymes form transient covalent interactions with DNA during their physiological reaction cycles. The best-studied enzymatic DPCs are topoisomerases 1 and 2α (Topo1 and Topo2α), known as Topo1- or Topo2α-cleavage complexes (Topo-ccs) (Ashour et al., 2015, Maede et al., 2014). Topo1-ccs or Topo2-ccs are removed by tyrosyl-DNA phosphodiesterase-1 or -2 (TDP1 or TDP2), after proteolysis of Topos into small peptide fragments (max 15–108 amino acids) by an unknown mechanism (Debéthune et al., 2002, Interthal and Champoux, 2011). This suggests the existence of a protease that processes Topos upstream of TDP1 or 2 (Zhang et al., 2006). Despite the frequent occurrence of endogenous non-enzymatic and enzymatic DPCs in cells, the mechanism of DPC removal is still largely unknown (Ide et al., 2015).

Studies in bacteria, yeast, and higher eukaryotes suggest that several canonical DNA repair pathways, including nucleotide excision repair, homologous recombination, and Fanconi anemia repair pathway together with proteasome-dependent protein degradation are involved in the removal of DPCs (Barker et al., 2005, de Graaf et al., 2009, Nakano et al., 2007, Salem et al., 2009). Recently, a yeast DNA-dependent protease Wss1 (weak suppressor of smt3) was shown to protect yeast cells from FA-induced toxicity and, in coordination with Tdp1, process Topo1-ccs (Stingele et al., 2014).

We are currently not aware of any specialized DPC repair pathway in metazoans, although DPC removal is essential for DNA replication fork progression (Kuo et al., 2007, Reardon et al., 2006). Recent biochemical data in *Xenopus* egg extract demonstrated that DPC removal is coupled to DNA replication in a proteasome-independent, but protease-dependent manner (Duxin et al., 2014). However, the protease required for the removal of DPCs during DNA replication remained unknown (Duxin and Walter, 2015, Reardon et al., 2006).

Ruijs-Aalfs syndrome (RJALS), also known as SPARTAN syndrome, is a human autosomal recessive disease characterized by chromosomal instability, premature aging, and early onset hepatocellular carcinoma in children. RJALS is caused by monogenic and biallelic mutations in *SPRTN* (*DVC1*), and a single missense mutation in a putative metalloprotease SprT domain (SPRTN^Y117C^) is pathogenic and responsible for premature aging and liver cancer in humans (Lessel et al., 2014, Ramadan et al., 2016). While the roles of the C-terminal domains of SPRTN have been extensively characterized in translesion DNA synthesis and recruitment to DNA damage foci, the function of the SprT domain, localized in the N-terminal part, is completely unknown (Centore et al., 2012, Davis et al., 2012, Ghosal et al., 2012, Mosbech et al., 2012). At the cellular level, RJALS cells exhibit DNA replication stress, specifically slower replication, and increased numbers of stalled forks and DNA double-strand breaks (Lessel et al., 2014).

Given the importance of SPRTN in genome stability, and the fact that bioinformatic analysis suggests that SPRTN and the yeast protease Wss1 are both distantly related to the Zinicin family of metalloproteases (Stingele et al., 2015), we asked whether SPRTN is a metalloprotease responsible for DPC repair.

Here, we show that SPRTN is a DNA-dependent protease that protects human proliferative cells from DPC toxicity. SPRTN associates with the DNA replication machinery and removes DPCs during DNA synthesis, and thus RJALS is caused by a defect in DPC repair. Altogether, we identified the mechanism for DPC removal from chromatin in human cells and highlighted the importance of this mechanism for genome stability and its relevance for human pathogenesis in accelerated aging and carcinogenesis.

## Results

### SPRTN Prevents Accumulation of Endogenous DNA-Protein Crosslinks

**Figure 1:**
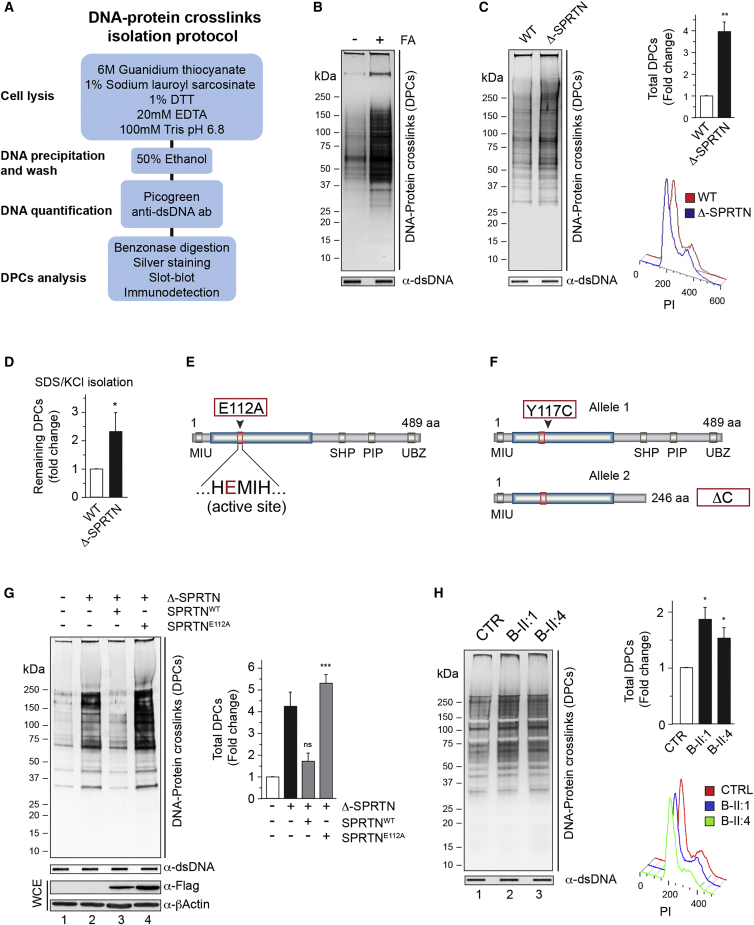
SPRTN Prevents Basal DNA-Protein Crosslinks Accumulation (A) Schematic of DPC isolation protocol (RADAR). (B) DPC accumulation in HeLa cells after FA treatment (2.5 mM, 30min). (C) SPRTN deficiency leads to DPCs accumulation (silver staining). The quantification and cell-cycle profiles are shown (right images). (D) Quantification of DPC isolates by SDS/KCl precipitation method. (E) Schematic of SPRTN protein domains and a putative protease active site. (F) Schematics of biallelic SPRTN mutations in RJALS patients. (G) Total DPC levels after ectopic expression of SPRTN WT or E112A in Δ-SPRTN HeLa cells and corresponding quantification. whole cell extract: WCE. (H) Total DPC levels in RJALS patient LCLs from family B (two patients; B-II:1 and B-II:4) and control LCLs (CTR). The quantification and cell-cycle profiles are shown (right image). Mean ± SEM, n = 3. See also Figure S1.

To investigate the role of SPRTN in DPC repair, we isolated total genomic DNA from HeLa cells and analyzed the amount of DPCs using rapid approach to DNA adducts recovery (RADAR) coupled to SDS-PAGE/silver staining (Figure 1A) (Kiianitsa and Maizels, 2013). The isolation of DNA under stringent denaturing conditions allows us to exclusively detect proteins that are crosslinked to DNA (DPCs). DPC isolates were quantified for total DNA amount to ensure equal amount of DNA for DPC analysis and then treated with Benzonase, to remove all DNA and RNA, prior to SDS-PAGE/silver staining detection. Proteinase K treatment of Benzonase-treated DPCs confirms specificity of protein staining by the silver-staining method (Figure S1A). In addition, treating the cells with FA, camptothecin (CPT), or etoposide (ETO), known DPC-inducing agents, as expected causes a huge accumulation of general (Figure 1B) or specific DPCs (Figure S1B).

Quantitative analysis of total DPCs by silver staining in different cell lines (HeLa, HEK293, and T24) revealed that SPRTN depletion resulted in a 2- to 5-fold increase in the total amount of proteins covalently attached to DNA (Figures S1C and S1D and data not shown). Similarly, CRISPR/Cas9-created SPRTN partial knockout HeLa cells (Δ-SPRTN, Figures S1E–S1G) showed a 3- to 4-fold increase in total DPCs (Figure 1C). Accumulation of DPCs observed in Δ-SPRTN cells was further confirmed by SDS/KCl precipitation assay, an indirect method for DPC isolation (Figure 1D). The increase in DPCs in SPRTN-deficient cells was not due to differences in cell-cycle stage (Figures 1C, S1C, and S1D). To investigate if a putative protease domain in SPRTN plays a role in DPC removal, we ectopically expressed SPRTN wild-type (SPRTN^WT^) or E112A (SPRTN^E112A^), a variant containing a glutamic acid to alanine change in a predicted protease active center (HEXXH, H; histidine, E; glutamic acid, X; and any amino acid; Figure 1E), in Δ-SPRTN HeLa cells. Overexpression of SPRTN^WT^, but not SPRTN^E112A^, completely rescued basal DPC accumulation (Figure 1G, compare lanes 3 and 4). To investigate whether RJALS lymphoblastoid cell lines (LCLs) with monogenic and biallelic mutations in *SPRTN* (Figure 1F; SPRTN-ΔC/SPRTN^Y117C^) are also deficient in removal of DPCs, we isolated and analyzed total DPCs in these cells (Figure 1H). RJALS cells showed a 1.5- to 2-fold increase in total DPCs when compared to control LCLs (Figure 1H, compare lane 1 to lanes 2 and 3).

The nucleotide excision repair (NER) and homologous recombination (HR) pathways have also been implicated in DPC repair. However, depletion of the main components of NER (XPC) and HR (MRE11) by small interfering (si)RNA did not lead to an increase to the total amount of DPCs (Figure S2A). Similarly, impairment of the DNA interstrand crosslink repair pathway, Fanconi anemia, by depletion of FANCD2 did not cause DPC accumulation compared to control cells (Figure S2A). Altogether, these results indicate that SPRTN is the main player involved in DPC removal, and, more specifically, that its putative metalloprotease active residue (E112), is essential for removal of endogenously occurring DPCs in human proliferative cells.

### RJALS and SPRTN-Depleted Cells Are Hypersensitive to DPC-Inducing Agents

**Figure 2:**
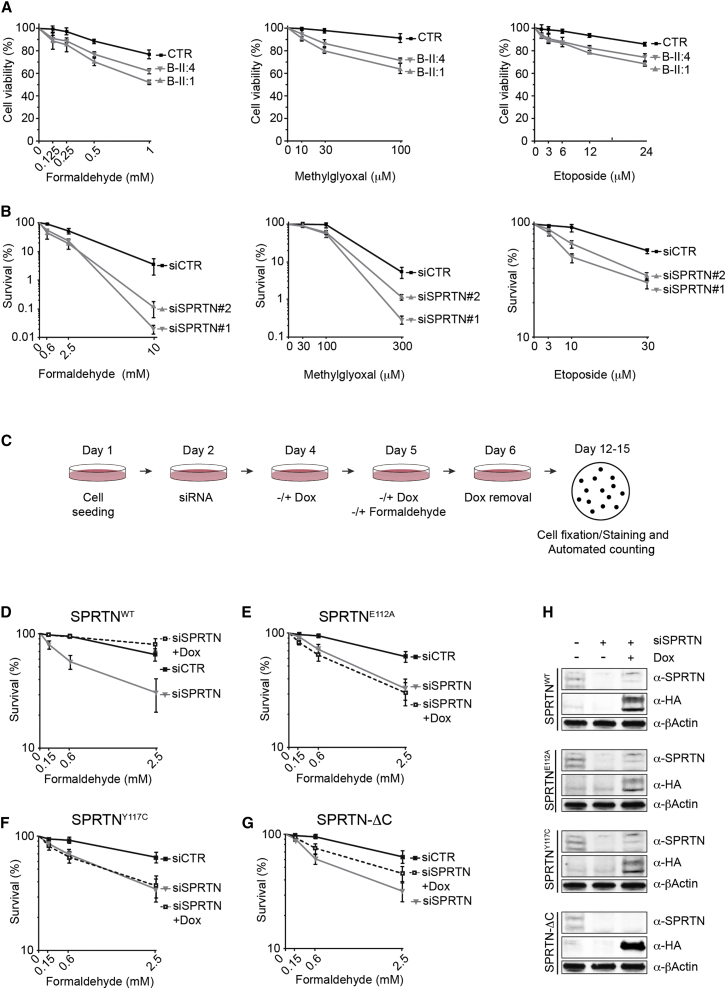
RJALS and SPRTN-Depleted Cells Are Hypersensitive to DPC-Inducing Agents (A) Cell viability assays of RJALS (B-II:1 and B-II:4) and control (CTR) LCLs after treatment with indicated chemicals. (B) Clonogenic survival assay of siRNA control (CTR) or siRNA SPRTN-depleted HeLa cells after treatment with indicated chemicals. (C) Schematic of experimental setup for clonogenic survival assays in doxycycline inducible (+Dox) SPRTN Flp-In HeLa cell lines. (D–G) Ectopic expression of SPRTN^WT^ (D), SPRTN^E112A^ (E), SPRTN^Y117C^ (F), and SPRTN-ΔC (G) after depletion of endogenous SPRTN. (H) Western blot (WB) demonstrating SPRTN depletion and/or overexpression. Mean ± SEM, n = 3. See also Figure S2.

We further asked whether RJALS patient LCLs and siRNA SPRTN-depleted HeLa cells are hypersensitive to DPC-inducing agents. We used FA and methylglyoxal, which induce general DPCs (Figure 1B and data not shown), and CPT and ETO, which induce specific enzymatic DPCs (Figure S1B). Both RJALS patient cells (Figure 2A) and SPRTN-depleted cells (Figures 2B and S2B) were hypersensitive to DPC-inducing agents. To analyze how individual SPRTN mutations found in RJALS patients affect cell sensitivity to DPC-inducing agents, we created doxycycline (DOX) inducible and stable Flp-In HeLa cell lines, expressing wild-type (WT), enzymatic dead protein (E112A), and patient variants of SPRTN (Figures 2C–2H and S2C–S2F). Ectopic expression of SPRTN^WT^, where endogenous SPRTN was depleted with siRNA targeting the 3′ UTR of SPRTN transcripts, rescued SPRTN-depleted cells’ hypersensitivity to FA (Figure 2D) or CPT (Figure S2C). Conversely, overexpression of SPRTN^E112A^ or patient variant SPRTN^Y117C^ was not able to rescue hypersensitivity to FA (Figures 2E and 2F, respectively) or CPT (Figures S2D and S2E, respectively). Ectopic expression of the truncated patient variant SPRTN-ΔC, which still contains an intact putative metalloprotease domain, partially rescued hypersensitivity to FA (Figure 2G) or CPT (Figure S2F). Both patient mutations are defective in their ability to protect cells from DPCs, although SPRTN-ΔC to a lesser extent, since RJALS LCLs are hypersensitive to DPC-inducing agents (Figure 2A).

Next, to address if the cytotoxicity observed in SPRTN-deficient cells is due to accumulation of DPCs, we co-depleted Topo1 and SPRTN and monitored cell survival following CPT treatment. Co-depletion of Topo1 completely rescued sensitivity to CPT in SPRTN-depleted cells (Figure S2G), confirming that CPT-induced toxicity in SPRTN-depleted cells is due to accumulation of Topo1-ccs. Co-depletion of TDP1, a key player in Topo1-cc removal, does not further hypersensitize SPRTN-depleted cells to CPT, suggesting that both proteins work in the same pathway (Figure S2H).

The Fanconi anemia pathway is known to protect from formaldehyde-induced toxicity. As expected, inactivation of the Fanconi anemia pathway by FANCD2 siRNA depletion hypersensitizes cells to FA-treatment, but not CPT-treatment (Figure S2I). This further suggests that the Fanconi anemia pathway is strictly involved in repair of DNA-interstrand crosslinks (also induced by FA treatment), but not DNA-protein crosslinks (e.g., removal of a specific DPC induced by CPT). Altogether, these results suggest that SPRTN forms a unique DNA repair pathway for DPC removal.

### SPRTN Is a DNA-Dependent Metalloprotease

Intensive work from several laboratories was unable to identify SPRTN protease activity (Davis et al., 2012, Kim et al., 2013, Mosbech et al., 2012). Our results led us to re-evaluate published data and investigate whether SPRTN is indeed a protease. To this end, we purified SPRTN^WT^, SPRTN^E112A^, two patient variants (SPRTN^Y117C^ and SPRTN-ΔC), and several C-terminally truncated variants of SPRTN using an *E. coli* protein expression system.

**Figure 3:**
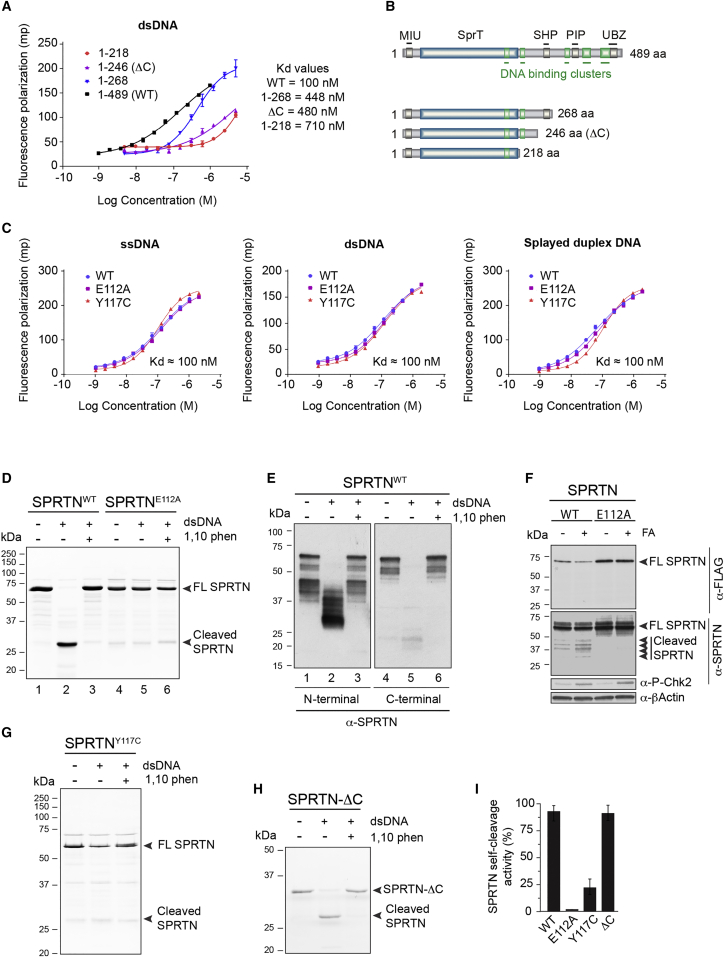
SPRTN Is a DNA-Dependent Metalloprotease with Auto-Proteolytic Properties (A) Fluorescence polarization DNA binding assay of SPRTN and its C-terminally truncated variants. (B) Schematic of in silico predicted DNA binding regions (green squares) in SPRTN protein and in SPRTN C-terminally truncated variants. (C) DNA binding affinity of SPRTN^WT^, SPRTN^E112A^, and SPRTN^Y117C^ toward ds, ss, and splayed DNA. (D) DNA induces auto-proteolysis of SPRTN. E112A mutation or 1,10 phenanthroline (phen) inhibits SPRTN activity. The cleavage was analyzed on SDS-PAGE gels and visualized by Coomassie blue staining. (E) WB illustrating SPRTN auto-cleavage. (F) WB of total cell lysates after ectopic expression of FLAG-SPRTN (WT or E112A) after FA treatment. The anti-phospho (P) Chk2 represents a positive control for activation of DNA damage signaling. (G and H) In vitro enzymatic reactions as in (D), with patient mutations as indicated. (I) Quantification of auto-cleavage activity. Mean ± SD, n = 3. See also Figure S3.

Considering that the SprT domain is classified as a putative metalloprotease domain, we first analyzed the presence of metal in the SPRTN protein. We purified SPRTN lacking the C-terminal Zn-binding UBZ domain and PIP-box (SPRTN 1–268) and analyzed it by mass spectrometry. Intact protein analysis of SPRTN under native and denaturing conditions revealed a mass increase of 126 daltons, which is consistent with the presence of two zinc ions (Figure S3A). The recent finding that the yeast protease Wss1 requires DNA to elicit proteolytic activity (Balakirev et al., 2015, Stingele et al., 2014) led us to investigate whether SPRTN binds DNA. We used 63 bp double-strand DNA probes (dsDNA) labeled with fluorescein isothiocyanate (FITC) to analyze SPRTN binding affinity by fluorescence polarization. SPRTN^WT^ protein showed high affinity to dsDNA (dissociation constant [K_D_] ≈100 nM) (Figure 3A). In silico analysis of the SPRTN secondary structure revealed the presence of five DNA binding regions: four motifs in the C-terminal part of the protein and one in the SprT protease domain (Figures 3B and S3B). The removal of predicted C-terminal DNA binding sites strongly decreased the DNA binding affinity of SPRTN (Figure 3A).

Next, we asked whether SPRTN binds different DNA structures. SPRTN^WT^ binds single-strand DNA (ssDNA), dsDNA, and splayed DNA with similar affinities (Figure 3C). SPRTN^E112A^ and SPRTN^Y117C^ exhibited similar affinities to DNA as SPRTN^WT^. The truncated variant of SPRTN-ΔC (1–246) showed reduced affinity to DNA (K_D_ ≈ 0.48 μM; Figure 3A), which correlates with the loss of putative DNA-binding sites (Figures 3B and S3B).

We observed increased levels of SPRTN^WT^ degradation when the reaction of SPRTN and DNA was incubated for a longer period of time and analyzed by SDS-PAGE/Coomassie blue staining. Full-length SPRTN^WT^ protein was mostly intact and ran at the predicted size of 55 kDa (Figure 3D, lane 1). However, incubation of SPRTN^WT^ with dsDNA induced strong degradation of SPRTN with several visible protein bands and a prominent accumulation of a ∼25 kDa fragment (Figure 3D, lane 2). Mass spectrometry analysis of SPRTN^WT^ degradation products identified at least five different cleavage products in the SPRTN protein (Figure S7C), with cleavage sites located in its C-terminal part (Figure S3C). SPRTN^WT^ auto-cleavage activity was inhibited in the presence of 1,10 phenanthroline, a known inhibitor of Zn^2+^-dependent metalloproteases (Figure 3D, lane 3). SPRTN auto-cleavage activity was abolished by the E112A mutation (Figure 3D, lane 5). Western blot analysis of SPRTN^WT^ auto-cleavage products with antibodies raised against N- or C-terminal fragments of SPRTN further confirmed mass spectrometry data and demonstrated that SPRTN protein was mainly cleaved within its C-terminal part, while the N-terminal fragment remained mostly intact (Figure 3E, lanes 2 and 5). These data suggest that SPRTN is a DNA and Zn-dependent protease, which possesses auto-cleavage activity in vitro. SPRTN^WT^, but not SPRTN^E112A^, auto-cleavage products were also visible in HEK293 cells, especially after FA treatment, demonstrating SPRTN auto-cleavage activity in vivo (Figure 3F).

We further asked what is the minimum length of DNA required to activate SPRTN cleavage activity (Figure S3D). Different sized probes of ss or dsDNA were incubated with SPRTN^WT^ protein. DNA fragments of 100-mer ssDNA or dsDNA were the most efficient activators of SPRTN auto-cleavage activity.

In order to investigate if RJALS patient mutations are defective with respect to their auto-cleavage activity, we tested the two patient variants, SPRTN^Y117C^ and SPRTN-ΔC (Figures 3G–3I). SPRTN-ΔC showed similar auto-cleavage activity to SPRTN^WT^, whereas SPRTN^Y117C^ showed an ∼80% reduction in auto-cleavage activity compared to SPRTN^WT^ (Figure 3I). Considering that SPRTN^Y117C^ binds DNA with similar affinity to SPRTN^WT^, we conclude that its enzymatic deficiency is not due to defective DNA binding. Our results suggest that SPRTN^Y117C^ directly affects the protease active center (E112), which is located only five amino acids upstream of the mutation. SPRTN-ΔC (1–246) still retains auto-cleavage activity similar to SPRTN^WT^ (Figures 3H and 3I) despite its lower DNA affinity (Figure 3A).

### Trans-Cleavage Activity of SPRTN Metalloprotease

To address whether SPRTN auto-cleavage occurs in *cis* or in *trans*, we incubated SPRTN^WT^ with the enzymatic-dead variant of SPRTN (SPRTN^E112A^). SPRTN^WT^ cleaved SPRTN^E112A^ (Figure S3E, compare lanes 2 and 4). These data suggest that SPRTN cleaves itself in *trans*. To test the importance of DNA-binding for trans-cleavage activity, we incubated two C-terminal truncated variants of SPRTN that have medium (SPRTN^1–268^) or low (SPRTN^1–218^) DNA affinity with SPRTN^E112A^ (Figure 3A for DNA affinity). SPRTN^1–268^ cleaved SPRTN^E112A^ with similar efficiency to SPRTN^WT^ (Figure S3E, compare lane 4 to lane 6). In contrast, SPRTN^1–218^ markedly lost trans-cleavage activity (Figure S3E, compare lane 4 to lane 8). Next, we asked if DNA is a scaffold or an allosteric activator for SPRTN auto-cleavage activity. SPRTN^WT^ auto-cleavage activity was maximal at equimolar concentrations of protein and DNA in the reaction. Increasing the concentration of DNA gradually inhibited SPRTN activity (Figure S3F). Altogether, these results show that DNA serves as a scaffold, which brings SPRTN and its substrate into proximity, rather than acting as an allosteric activator. Loss of its C-terminal DNA binding regions renders SPRTN unable to perform its proteolytic activity, suggesting that auto-cleavage reduces the proteolytic activity of the cleaved forms.

### Identification of SPRTN Substrates

We isolated DPCs from control and SPRTN-depleted HeLa cells (Figure S4A) and analyzed them by label-free quantitative mass spectrometry. Three independent experiments revealed 84 significantly increased (1.5-fold) proteins within the DPCs in SPRTN-depleted cells, in comparison to control cells (Figure S4B and Raw data repository: PRIDE). Although many of the identified substrates were DNA- and RNA-binding proteins, the majority were non-DNA binding proteins (Figure S4C). Given that any protein in close vicinity to DNA can be crosslinked, especially nuclear matrix proteins, this is not a surprising finding. Accordingly, non-DNA binding proteins such as Lamin B1 and DNA-PK are enriched in DPCs isolated from Δ-SPRTN cells (Figure 5G).

**Figure 4:**
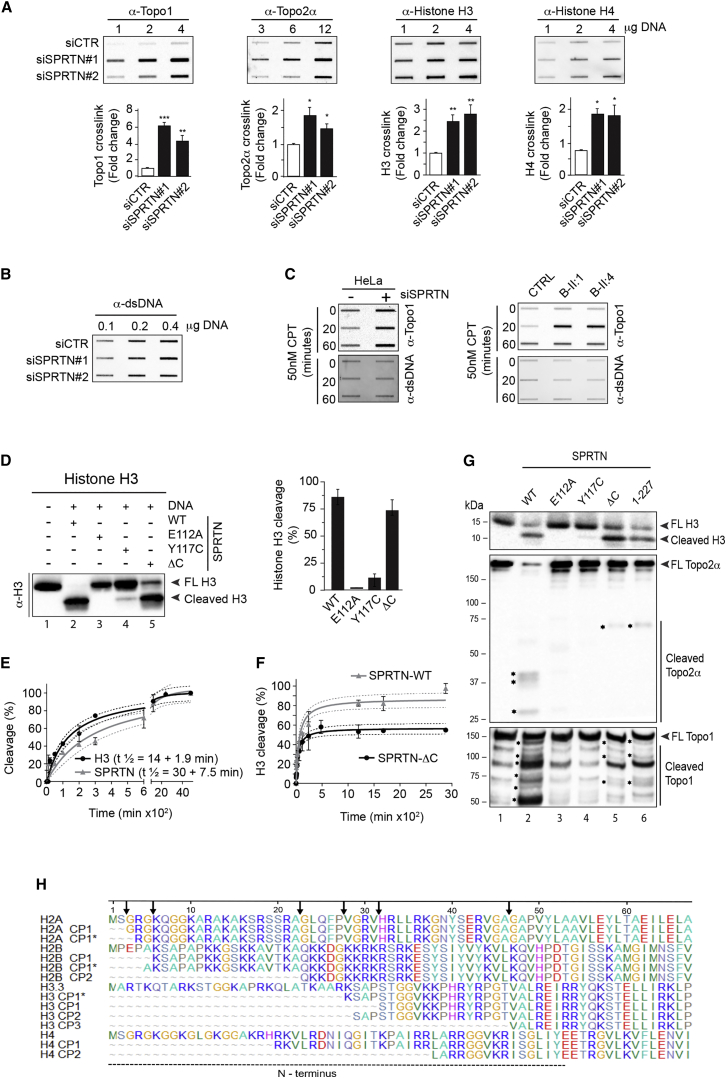
Identification of SPRTN Substrates (A) Slot-blots showing presence of Topo1, Topo2α, H3, and H4 DPCs after SPRTN depletion in HeLa cells and corresponding quantifications. Mean ± SEM, n = 3. (B) DNA loading controls for DPC analysis, prior to benzonase treatment as in (A). (C) Slot-blots showing accumulation of Topo1-ccs after continuous CPT treatment, as indicated, in Δ-SPRTN cells (left image) and RJALS LCLs (right image). (D) WB and corresponding quantification showing that SPRTN cleaves histone H3 in the presence of DNA. Mean ± SD, n = 3. (E) Time kinetics of H3 cleavage versus SPRTN self-cleavage within the same reaction mixtures expressed as increase in cleavage of either H3 or SPRTN over time (min). Mean ± SD, n = 3. (F) Comparison of time, response of H3 cleavage for SPRTN^WT^, and SPRTN-ΔC as in (D). y axis was normalized to a 0%–100% scale. Mean ± SD, n = 3. (G) Histone H3, Topo1 and Topo2α are substrates of SPRTN protease. The cleavage products (^∗^) were detected by WB using antibodies against histone H3 (upper image), Topo2α (middle image), or Topo1 (lower image). FL, full length. (H) Multiple sequence alignment of histones’ cleavage products (CP) showing cleavage sites (arrow) in their unstructured N-terminal tails (^∗^ denotes alternative cleavage products). See also Figures S4 and S5.

We focused on histones and DNA topoisomerases, DNA-binding proteins with well-characterized cellular functions, which emerged among the top hits (≥1.98-fold increase) in our mass spectrometry analysis (Figure S4D). To validate mass spectrometry data, we analyzed specific DPCs, isolated as shown in Figure 1A, by slot-blot immunodetection. Depletion of SPRTN with two different siRNAs significantly increased the amount of Topo1, Topo2α, histone H3, and histone H4 (Figures 4A and 4B). To demonstrate that hyperaccumulation of Topo1 was specific to loss of SPRTN protease activity, we ectopically expressed SPRTN^WT^ or SPRTN^E112A^ in 3′ UTR-targeted siRNA SPRTN-depleted cells. Ectopic expression of SPRTN^WT^, but not SPRTN^E112A^, rescued Topo1 accumulation (Topo1-cc) (Figure S4E). We further analyzed Topo1-ccs following treatment with CPT (Figure 4C). Both SPRTN-depleted cells and patient LCLs accumulate more Topo1-ccs compared with control cells, thus further confirming the fundamental importance of SPRTN protease activity in the removal of Topo1-ccs and protection from DPC cytotoxicity (Figures S2B–S2H).

### Characterization of SPRTN Enzymatic Activity

To characterize the ability of SPRTN to cleave the identified substrates, we performed in vitro cleavage activity assays using purified SPRTN^WT^ protein. SPRTN^WT^ cleaved all tested histones, H2A, H2B, H3, and H4, in a DNA-dependant manner, but not the cytosolic protein glutathione S-transferase (Figures 4D, S5A, and S5B). Kinetic analysis of SPRTN auto-cleavage and histone H3 cleavage in the same reaction revealed that SPRTN cleaves H3 with slightly faster kinetics than itself (Figure 4E). This suggests that SPRTN simultaneously cleaves substrates and itself, but with a higher preference for the substrate. This most probably leads to SPRTN inactivation as the cleaved SPRTN products lose DNA binding affinity (Figure 3A), enzymatic activity (Figure S3E), and have 2-fold lower substrate processivity kinetics (Figure 4F). This might be one of the mechanisms, beside cell-cycle regulation (see below), by which SPRTN protease self regulates to prevent deleterious and uncontrolled cleavage in its vicinity.

We next tested the cleavage activity of various SPRTN variants, focusing on histone H3 (Figures 4D and 4G). As expected, catalytically inactive SPRTN^E112A^ did not cleave histone H3. Similar to SPRTN auto-cleavage activity, patient variant SPRTN^Y117C^ was severely affected with respect to histone H3 cleavage (∼8% active), whereas SPRTN-ΔC retained similar activity to SPRTN^WT^. We extended the analysis of the cleavage activity to two other identified substrates, Topo1 and Topo2α. To this end, we purified YFP-Topo1 and GFP-Topo2α from whole cell extracts under denaturing conditions and incubated them with different variants of SPRTN. SPRTN^WT^, but not SPRTN^E112A^, cleaved Topo1 and Topo2α in vitro, thus confirming Topo1 and Topo2α as SPRTN substrates (Figure 4G, lanes 2 and 3). Characterization of patient mutations revealed that SPRTN^Y117C^ was unable to cleave Topo1 and Topo2α. Unexpectedly, in contrast to auto-cleavage (Figure 3H) and cleavage of histone H3 (Figures 4D and 4G), SPRTN-ΔC showed severely reduced proteolysis of Topo1 and Topo2α in vitro, similar to the smallest auto-cleaved form of SPRTN (1–227) (Figure 4G, lanes 5 and 6). These data show that SPRTN protease cleaves various DNA-binding substrates in a DNA-dependent manner. SPRTN protease activity is severely hampered by patient mutation Y117C for all tested substrates. Patient truncated variant SPRTN-ΔC, although able to cleave itself and histone H3, exhibits strongly reduced activity toward Topo1 and Topo2α, suggesting that the C-terminal part of SPRTN is required for optimal proteolysis of these substrates.

### SPRTN Is a Pleiotropic Protease for DNA-Binding Proteins

Next, we aimed to identify SPRTN protease cleavage sites and asked if SPRTN protease cleaves at a specific amino acid sequence motif. Cleavage products of H2A, H2B, H3, and H4 as well as auto-cleavage products of SPRTN were analyzed by mass spectrometry to identify cleavage sites (Figures 4H and S5C). We found that SPRTN cleaves histones H2A, H2B, H3, and H4 within their unstructured, positively charged N-terminal tail. All identified cleavage regions were enriched in arginine and lysine residues and were in very close proximity to serine residues in most cases. Analysis of SPRTN auto-cleavage sites (CS) also showed an abundance of lysine, arginine, and serine residues (CS 1 and 3), while specifically CS3 is heavily enriched in serines (and, to a lesser extent, lysines and arginines) (Figure S3C). Similar to cleavage sites of histones, which are present in disordered protein regions (N terminus), SPRTN cleaves itself in multiple places in its C terminus, which is predominantly disordered (Figure S5D). These results suggest that SPRTN is not a sequence-specific protease, but cleaves unstructured protein regions in the vicinity of lysine, arginine, and serine residues.

To test whether SPRTN also cleaves DPCs in vitro, we isolated total DPCs by SDS/KCl precipitation assay and incubated them with recombinant SPRTN. SPRTN^WT^, but not SPRTN^E112A^, cleaves DPCs in vitro (Figure S6A). Thus far, our in vitro and in vivo results suggest that SPRTN cleaves DNA-binding proteins regardless of their DNA-binding status (i.e., covalent DPCs or non-covalent). To further test this observation, we isolated chromatin-bound Topo1 or Topo2 under denaturing conditions from both untreated HEK293 cells and those treated with CPT or ETO, respectively. SPRTN cleavage efficiency of immunopurified Topo1 and Topo2α was similar between untreated and Topo1/2α-cc enriched samples (CPT- and ETO-treated, respectively; Figures S6B and S6C). Altogether, these data suggest that SPRTN does not specifically cleave DPCs, but DNA-binding substrates, and that SPRTN is indeed a pleiotropic protease as it cleaves the majority of high-molecular weight DPCs in vitro (Figure S6A, compare lanes 1 and 2).

### SPRTN Removes DPCs during S-Phase and Protects Proliferative Cells from DPC-Inducing Agents

**Figure 5:**
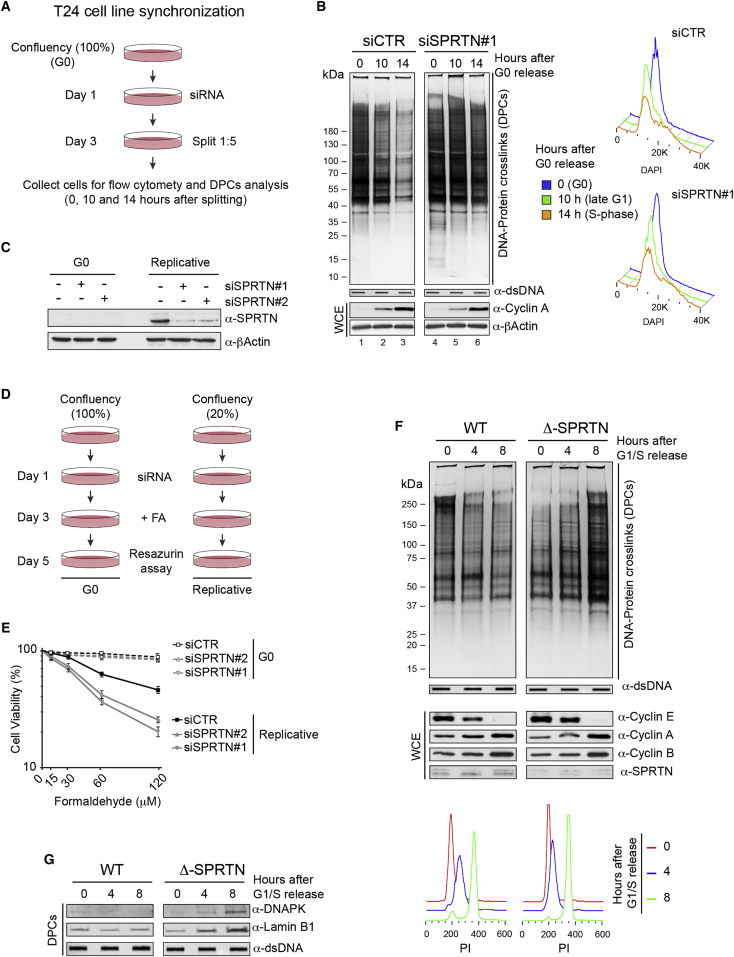
DPC Removal during S-Phase of the Cell Cycle (A) Schematic of the experimental approach used to synchronize T24 cell in G0 and monitor DPC levels during S-phase entry. (B) Total DPC levels in G0-, late G1-, and S-phase before and after SPRTN depletion in HeLa cells visualized by silver staining. The WB of cyclin A and cell-cycle profiles were used as a control of S-phase entry. (C) WB showing SPRTN expression or depletion in non-replicative and replicative T24 cells. (D) Schematic of cell viability measurement in replicative and non-replicative cells. (E) Cell viability of replicative and non-replicative T24 cells after SPRTN depletion and FA treatment. (F) Total DPC levels after G1/S release in HeLa WT and Δ-SPRTN cells visualized by silver staining. The WB of cyclins and cell-cycle profiles (lower images) were used to control S-phase progression. (G) WB showing presence of DNA-PK and Lamin B1 in DPCs in Δ-SPRTN HeLa cells after G1/S release. Whole cell extract: WCE. Mean ± SEM, n = 3.

Considering that SPRTN expression is absent in the G1-phase of the cell cycle, and is rapidly upregulated as cells enter the S-phase (Mosbech et al., 2012), we hypothesized that SPRTN is involved in the removal of DPCs during DNA synthesis. To test this hypothesis, we analyzed the levels of DPCs during S-phase progression in control and SPRTN-depleted cells. We used T24 cells, which arrest in the G0 phase by contact inhibition at 100% confluency and synchronously enter S-phase when diluted to lower densities. Cells were arrested in G0, treated with control-siRNA or SPRTN-siRNA for 2 days, and then diluted to enter S-phase (Figure 5A). We monitored the total amount of endogenous DPCs during S-phase progression. DPCs were rapidly removed as control cells progressed through S-phase. Conversely, SPRTN-depleted cells showed delayed kinetics of DPC removal during S-phase progression (Figure 5B, compare lanes 2 and 3 with 5 and 6). Western blot analysis of T24 cells revealed that SPRTN is not expressed in G0 cells (Figure 5C), further suggesting an essential role of SPRTN in protection from DPCs during DNA synthesis. To investigate this hypothesis, we treated G0-arrested or proliferative T24 cells with control or SPRTN siRNA for 2 days, exposed the cells to a sub-lethal dose of FA for an additional 2 days, and then monitored cell viability (Figure 5D). Indeed, only SPRTN-depleted proliferative, but not G0-arrested cells, were hypersensitive to FA (Figure 5E). Furthermore, Δ-SPRTN HeLa cells hugely accumulate total DPCs during S-phase progression compared to control cells (Figure 5F). These data show that SPRTN processes DPCs during S-phase progression, thus protecting cells from DPC cytotoxicity.

### SPRTN Is a Constitutive Part of the DNA Replication Machinery

**Figure 6:**
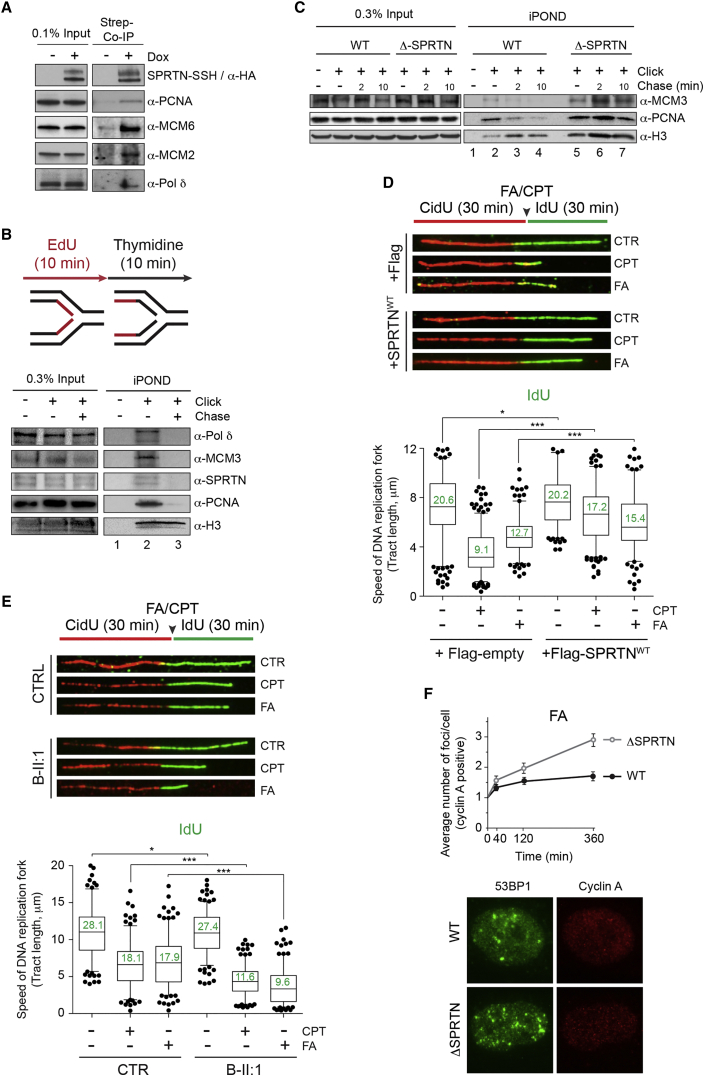
SPRTN Is a Part of the DNA Replication Machinery and Regulates DNA Replication Fork Progression (A) CoIP of SPRTN-Strep-Strep-His (SSH) from Flp-In HEK293 cells stably expressing SPRTN upon Dox induction showing association with replisome proteins. (B) Schematic of the iPOND approach (upper image). iPOND is illustrating that SPRTN moves with the replisome. HEK293 cells were treated with EdU for 10 min to label nascent DNA (lane 2) and then chased with thymidine where indicated (mature DNA, lane 3). (C) iPOND showing increased retention of replisome proteins on mature DNA in Δ-SPRTN HeLa cells (lane 6 and 7). (D) DNA fiber assay in HeLa cells after overexpression of FLAG-SPRTN^WT^ or empty FLAG-vector. The representative DNA fibers are shown (upper image). The quantification of IdU-labeled tract length in the presence of DPC-inducing agents (50 μM FA, 25 nM CPT) is shown. The numbers (in green) are mean values of tract length in kilobases. (E) DNA fiber assay in RJALS LCLs (B-II:1) and control LCLs after FA or CPT treatment performed as in (D). (F) Quantification of 53BP1 foci in cyclin A positive HeLa WT or Δ-SPRTN cells after FA treatment (50 μM). The data represent fold-change in 53BP1 foci compared to untreated cells (time point 0) (upper image). The representative micrographs of 53BP1 foci in Cyclin A positive cell at 6 hr after FA treatment are shown (lower image). Mean ± SEM, n = 3. See also Figure S6.

Having demonstrated that SPRTN protects proliferative human cells from DPCs and knowing that patient mutation SPRTN^Y117C^ is essential for unperturbed DNA replication fork progression (Lessel et al., 2014), we asked whether SPRTN is a part of the DNA replication machinery. We isolated SPRTN-SSH from total HEK293 cell extracts over Streptactin sepharose under high salt and detergent conditions to remove all unspecific binding proteins from SPRTN-complexes in vivo. SPRTN co-precipitated with the main components of the DNA replication machinery: PCNA, minichromosome maintenance complex (MCM) subunits 2 and 6 and DNA polymerase δ (Figure 6A). We next asked if SPRTN is physically present at sites of DNA replication forks. To address this question, we isolated proteins from nascent DNA by iPOND technology. Similar to PCNA, MCM3, and DNA polymerase δ, SPRTN was present on nascent DNA (Figure 6B, lane 2) and moved with the replisome as shown by thymidine chase (Figure 6B, lane 3). Altogether, these data suggest that SPRTN is part of the DNA replication machinery and moves with the replisome during DNA synthesis.

### SPRTN Orchestrates DNA Replication Fork Progression by Removal of DPCs

We further asked whether SPRTN inactivation affects the progression of the DNA replication machinery. We isolated DNA replication forks by iPOND and monitored their progression in control and Δ-SPRTN HeLa cells (Figure 6C). Knock out of SPRTN severely affected progression of DNA replication, as shown by retention of PCNA and MCM3 on mature DNA (thymidine chase, Figure 6C, compare lanes 3 and 4 with lanes 6 and 7). Defects in DNA replication fork progression in Δ-SPRTN cells were demonstrated by DNA fiber assay (Figure S6D). Stalled DNA replication forks in Δ-SPRTN cells accumulated Topo1 (Figure S6E), one of the substrates of SPRTN protease in vitro and in vivo. These results indicate that SPRTN regulates replisome progression and prevents accumulation of Topo1 at sites of DNA replication forks. We further asked whether SPRTN physically interacts with Topos. To this end, we co-precipitated SPRTN, as described earlier, and analyzed the presence of Topo1 and Topo2α in the SPRTN complex. Co-immunoprecipitation experiments revealed that SPRTN indeed forms a complex with both Topo1 and Topo2α in vivo (Figure S6F).

To investigate how DPCs affect the progression of DNA replication, we employed the DNA fiber assay (Figure 6D). Treatment of control cells with a low dose of FA or CPT caused a strong reduction of DNA synthesis, detected as a decrease in DNA replication fork velocity when the second nucleotide (IdU) was incubated along with FA or CPT. Ectopic expression of SPRTN^WT^ rescued the DPC-induced DNA replication fork progression defect in both control and Δ-SPRTN cells (Figures 6D and S6D). By measuring DNA replication fork velocity in RJALS LCLs, we found that RJALS cells showed a stronger reduction (∼2.5-fold) in DNA replication fork progression than control cells when challenged with FA or CPT (Figure 6E). Altogether, these results suggest that SPRTN is essential for the progression of DNA replication forks challenged with DPC-inducing agents and is a limiting factor in this process since reduced fork speed in FA/CPT-treated control or Δ-SPRTN cells can be rescued by SPRTN^WT^ overexpression.

### SPRTN Prevents DPC-Induced DSBs in the S-Phase

We hypothesized that the decreased velocity of DNA replication forks in SPRTN-depleted or RJALS cells in the presence of DPC-inducing agents leads to prolonged stalling of replisomes and consequently to DNA replication fork collapse, visualized as DNA ds breaks (DSBs), a phenotype observed in RJALS patients (Lessel et al., 2014). We continuously exposed control or Δ-SPRTN cells to mild doses of FA or CPT and monitored the formation of 53BP1 foci, a recognized marker for DSBs, over a period of 6 hr by immunofluorescence microscopy in fixed cells. Cyclin A staining was used as a marker for S-/G2-phase-positive cells. The formation of 53BP1 foci was induced in both cyclin A positive and negative cells after FA or CPT treatment, further confirming DPC-induced genotoxicity (Figures 6F and S6G–S6I). However, the average number of 53BP1 foci per cell was 2- to 3-fold increased after FA or CPT treatment in cyclin A-positive Δ-SPRTN HeLa cells compared to control HeLa cells (Figures 6F and S6G). In contrast, the number of 53BP1 foci in cyclin A-negative cells was the same in both Δ-SPRTN and control cells (Figures S6H and S6I). These results suggest that SPRTN prevents DPC-induced DSBs during DNA synthesis.

## Discussion

**Figure 7:**
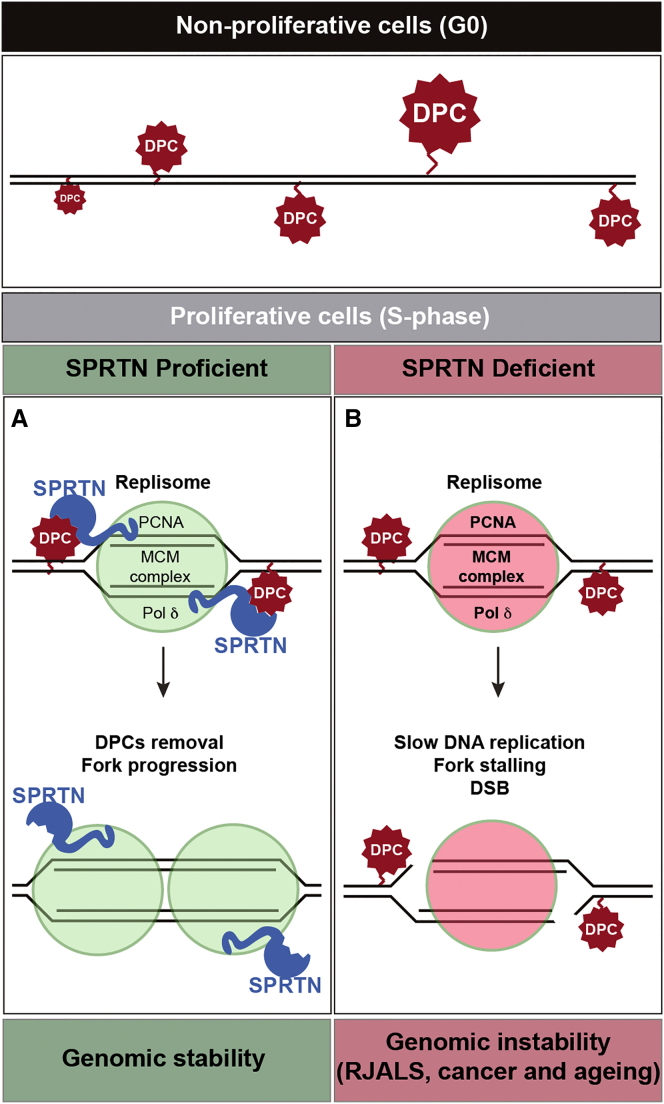
Model of DNA Replication-Coupled DPC Repair SPRTN is a constitutive part of the replisome and cleaves DPCs during replication fork progression. SPRTN protease protects proliferative cells from DPC-induced cytotoxicity (left). SPRTN deficiency (SPRTN mutations in RJALS, SPRTN haploinsufficient mice) causes stalling of the DNA replication fork due to pathological accumulation of DPCs, which in turn leads to DSBs and genomic instability (right).

We have revealed mechanistic insights into SPRTN protease activity for DPC repair and established SPRTN as a DNA replication-coupled protease (Figure 7). Our work identifies the missing protease involved in DNA replication and in the processing of Topoisomerase 1 and 2 crosslinks (Ashour et al., 2015, Duxin et al., 2014, Gao et al., 2014, Interthal and Champoux, 2011). Biochemical characterization of SPRTN mutations from RJALS patients shows that RJALS is caused by a defect in SPRTN protease activity, rendering it unable to process DPCs during DNA replication and therefore leading to DNA replication stress, one of the main causes of genome instability and cancer.

### DNA Replication-Coupled SPRTN Proteolysis

Our finding is in agreement with recent biochemical data in *Xenopus* egg extract, showing that DNA replication-coupled proteolysis is essential for DPC removal (Duxin et al., 2014), a model proposed by the Sancar laboratory (Reardon et al., 2006). However, the enzyme for this pathway remained unknown until now, when we show that SPRTN is an active protease and constitutive part of the replisome. Taking into consideration that DPCs are ubiquitous and that virtually any protein present in close proximity to DNA can be crosslinked, the proteolytic activity of SPRTN in DNA replication is essential. Cell-cycle dynamics of SPRTN expression support our hypothesis, with SPRTN being absent in G0 of T24-cells, and emerging upon entry into S-phase. This is in agreement with published data, showing that the level of SPRTN is downregulated in cells in G1 phase by the E3-ubiquitin ligase APC-complex and upregulated as cells enter S-phase (Mosbech et al., 2012). We therefore propose this to be a DNA repair pathway for the removal of DPCs in proliferative cells. We cannot exclude a potential role for SPRTN in non-proliferative cells, but if it exists, this function is not associated with DNA replication-coupled DPC repair.

### DNA-Protein Crosslink Repair

Our work addresses an emerging question in the field of DNA repair: how are DPCs removed from chromatin? Despite the frequent occurrence of DPCs, we poorly understand the process of DPC repair (Ide et al., 2015). It is believed that DPC repair partially depends on two canonical DNA repair pathways, NER and HR (Barker et al., 2005, Nakano et al., 2007). However, both repair pathways are limited by the fact that NER can only act on smaller protein crosslinks, not larger than 11 kDa, and HR relies on DSB formation that leads to recombinogenic events and associated genomic instability. As a consequence, other repair mechanisms should be involved in DPC processing and maintenance of genomic stability. Indeed, DPC accumulation is neither observed upon inactivation of key NER and HR players nor the Fanconi anemia pathway (Figure S2A), suggesting that the SPRTN-dependent DPC repair pathway is the main, specialized pathway for DPC repair in human cells. Early embryonic lethality of SPRTN knockout mice (Maskey et al., 2014), and the identification of SPRTN as one of the essential genes in the human genome (Hart et al., 2015, Wang et al., 2015), further supports the conclusion that SPRTN is a critical component of the unique DNA repair system, namely, DPC repair pathway.

### Characterization of RJALS Patient Mutations

We demonstrated that the protease activity of patient mutation SPRTN^Y117C^ is severely affected on all tested substrates. The reason why the Y117C mutation affects SPRTN enzymatic activity is not clear. Sequence comparisons show that this residue is not strictly conserved among SPRTN homologs (Figure S7A), although it is commonly hydrophobic, even among the wider zincin family. The close proximity of Y117 to the catalytic center, located only two residues downstream of the second potential Zn-binding histidine (H115), and the non-conservative nature of the substitution indicate that the variant may be unable to form the active site with the correct geometry. This is possibly a result of steric effects involving the positioning of the end of the HEXXH-containing helix and the subsequent loop containing the third potential Zn^2+^ ligand.

The other pathogenic mutation, SPRTN-ΔC, is proteolytically active toward itself (auto-cleavage) and histones (Figures 3H and 4D), but is severely impaired in processing Topo1 and Topo2α (Figure 4G). The sensitivity of SPRTN-depleted cells to CPT could not be completely restored by SPRTN-ΔC, suggesting that this patient mutation, although proteolytically active, cannot properly process certain substrates, such as Topo1 and Topo2. One reason could be that the C-terminal part is important for binding particular substrates and is involved in SPRTN substrate specificity. The increased levels of DPCs in RJALS patient cells, and the inability of DNA replication in RJALS cells to cope with the induction of non-enzymatic (FA) or enzymatic (CPT) DPCs, demonstrates that DPCs such as Topo1 and Topo2α, and most probably many others, are the main cause of RJALS syndrome. We propose that RJALS is a human disease linked to defective DPC repair pathway.

### Comparison between SPRTN and Wss1

Bioinformatic analysis suggests that SPRTN and Wss1 have a common ancestor (Stingele et al., 2015). While it is true that both enzymes are distantly related to the zinc metalloprotease superfamily, the two sequences can only be aligned over a relatively short 95 amino acid (aa) residue region with 24% sequence identity (comparing the human and *S. cerevisiae* enzymes) (Figure S7B). This common region includes the conserved HEXXH motif, shared among a wider set of enzymes with diverse functions, which provides the platform for Zn^2+^ binding via two histidines, with a neighboring glutamate residue thought to play a role in catalysis (Hooper, 1994). Beside this common core, no other regions of sequence similarity could be found. Wss1 and SPRTN show similar enzymatic properties, but their cellular function is partially uncoupled. SPRTN interacts with DNA replication machinery and binds ubiquitinated substrates via its Ub-binding domains, while Wss1 binds and processes SUMOylated substrates via its SUMO-interacting motifs (Balakirev et al., 2015). Inactivation of SPRTN causes a massive accumulation of total DPCs in proliferative human cells, including Topo1, Topo2α, and histones. In contrast, yeast cells lacking Wss1 do not accumulate total DPCs and do not show hypersensitivity to CPT (Stingele et al., 2014).

## Experimental Procedures

### DNA-Protein Crosslinks Isolation

DPCs were detected using a modified rapid approach to DNA adduct recovery (RADAR) assay (Kiianitsa and Maizels, 2013). The patient material used in this study (LCLs) was approved by the Human Research Ethics Committee of the Royal Children’s Hospital and the Oxford Research Ethics Committee Oxford Radcliffe Biobank (Ref.: 15/A156), University of Oxford. See the Supplemental Experimental Procedures for details.

### DNA-Protein Crosslinks Detection

Specific DPCs were detected using a vacuum slot-blot manifold (Bio-Rad) followed by immunodetection. DPCs were visualized using the Bio-Rad ChemiDoc XRS Plus Analyzer. See the Supplemental Experimental Procedures for details.

### Protein Purification

For overexpression in *E. coli* cells, SPRTN constructs were cloned in to either the pNIC-ZB vector (full-length SPRTN-WT, SPRTN-E112A, and SPRTN-Y117C), or pNIC28-Bsa4 vector (all other truncated constructs). For purification of full-length constructs containing a TEV cleavable Z-basic-his tag, cells lysates were applied to a Ni-sepharose IMAC gravity flow column. Elution fractions were applied directly to a 5 mL HiTrap SP HP column, washed, and eluted. The purification tag was cleaved with the addition of 1:20 mass ratio of His-tagged TEV protease during overnight dialysis. Protein identities were verified by LC/ESI-TOF mass spectrometry. See the Supplemental Experimental Procedures for details.

### Fluorescence Polarization DNA Binding Assays and DNA Probe Annealing

See the Supplemental Experimental Procedures for details.

### Generation of CRISPR/Cas9 SPRTN Partial Knockout HeLa Cells

See the Supplemental Experimental Procedures for details.

### SPRTN Substrate Cleavage Assay

SPRTN enzymatic reactions were performed in 150 mM NaCl and 25 mM Tris (pH 7.4) in a PCR block at 37°C. The reaction volume was typically 10 μL and contained: *E. coli* purified recombinant SPRTN (1–10 mg/mL solution), substrate (typically 1 mg/mL solution), and 100 bp dsDNA oligonucleotide probe. See the Supplemental Experimental Procedures for details.

### Isolation of Proteins On Nascent DNA

See the Supplemental Experimental Procedures for details.

### DNA Fiber Assay

The DNA fiber assay was performed as described previously (Lessel et al., 2014). See the Supplemental Experimental Procedures for details.

### Mass Spectrometry Analysis

See the Supplemental Experimental Procedures for details.

### Quantitative Proteomics and Data Analysis

See the Supplemental Experimental Procedures for details.

### Statistical Analysis

See the Supplemental Experimental Procedures for details.

## Author Contributions

B.V. performed in vivo and in vitro DPCs analysis, cellular toxicity, and biochemically characterized SPRTN self-cleavage activity. M.P. performed characterization of SPRTN enzymatic activity toward the substrates, identification of cleavage sites, and co-immunoprecipitation (coIP) experiments. J.A.N., H.A., and O.G. purified proteins and performed DNA binding assays. J.F. performed iPOND, and S.H. performed the DNA fiber assay. A.N.S. created stable cell lines. B.V., I.V., R.F., I.T., and B.M.K. performed mass spectrometry and data analysis. R.F. created reagents, G.W.M. provided reagents and analyzed data, and D.J.A. and P.J.L. secured RJALS/SPARTAN patient material. M.P. and J.A.N. analyzed the cleavage products. B.V. and N.D. analyzed DSB formation. B.V. and K.R. designed the majority of the experiments, and B.V., M.P., and K.R. analyzed data and prepared the manuscript. K.R. supervised the project.

## Accession Numbers

The accession number for the mass spectrometry raw data reported in this paper is PRIDE: PXD004154 (https://www.ebi.ac.uk/pride/archive/).

## Supplemental Information

Document S1. Supplemental Experimental Procedures and Figures S1–S7

Document S2. Article plus Supplemental Information
