# Supplementary material for: Metalloprotease SPRTN/DVC1 Orchestrates Replication-Coupled DNA-Protein Crosslink Repair
Source: Mol Cell. 2016 Nov 17;64(4):704–19. doi: 10.1016/j.molcel.2016.09.032 (PMC5128727; doi:10.1016/j.molcel.2016.09.032)
Supplement: Document S1. Supplemental Experimental Procedures and Figures S1–S7 [file mmc1.pdf]

Molecular Cell, Volume *64*

Supplemental Information

Metalloprotease SPRTN/DVC1 Orchestrates Replication-Coupled DNA-Protein Crosslink Repair

Bruno Vaz, Marta Popovic, Joseph A. Newman, John Fielden, Hazel Aitkenhead, Swagata Halder, Abhay Narayan Singh, Iolanda Vendrell, Roman Fischer, Ignacio Torrecilla, Neele Drobnitzky, Raimundo Freire, David J. Amor, Paul J. Lockhart, Benedikt M. Kessler, Gillies W. McKenna, Opher Gileadi, and Kristijan Ramadan

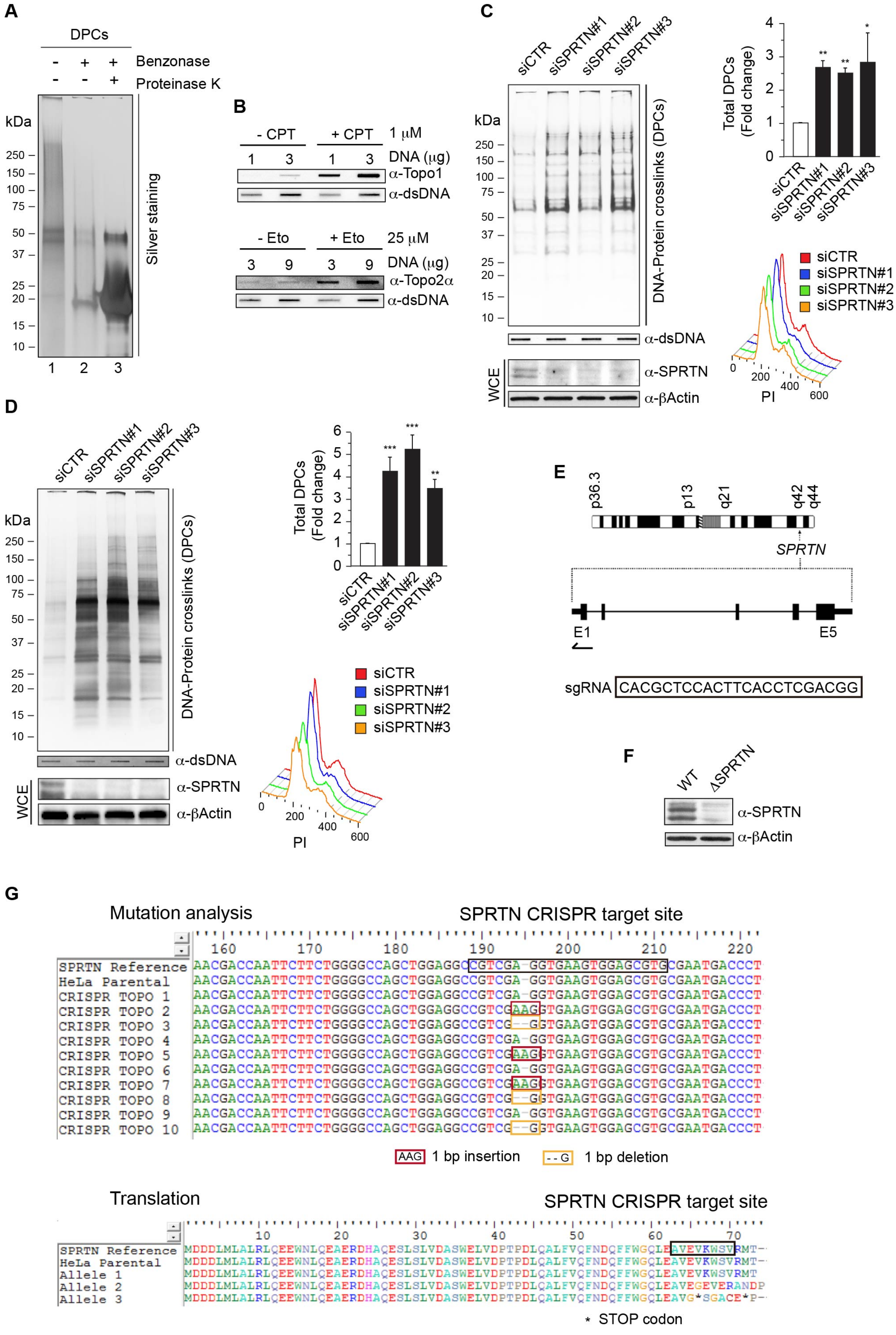

Figure S1 (referred to Figure 1)

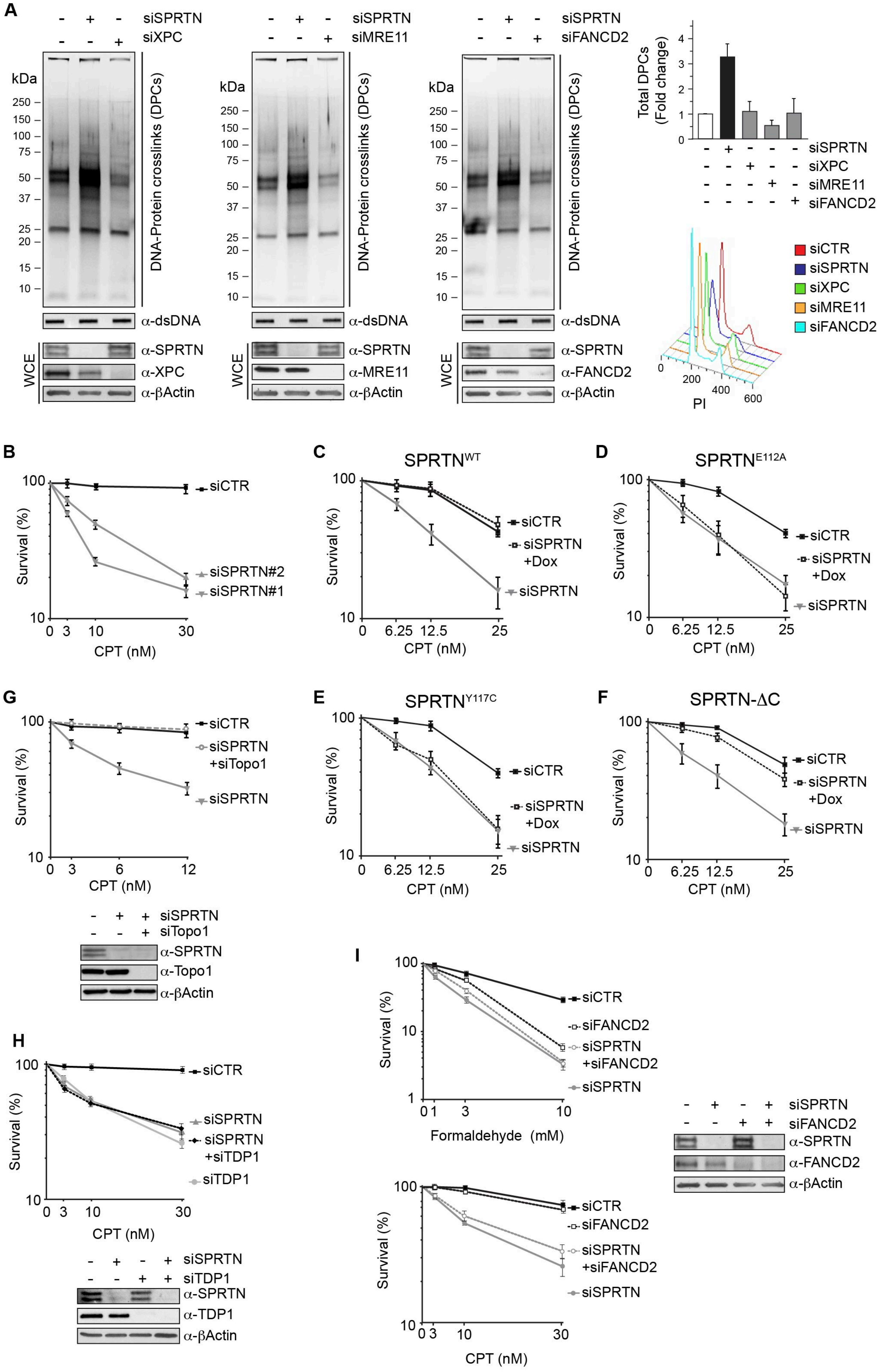

Figure S2 (referred to Figure 1 and 2)

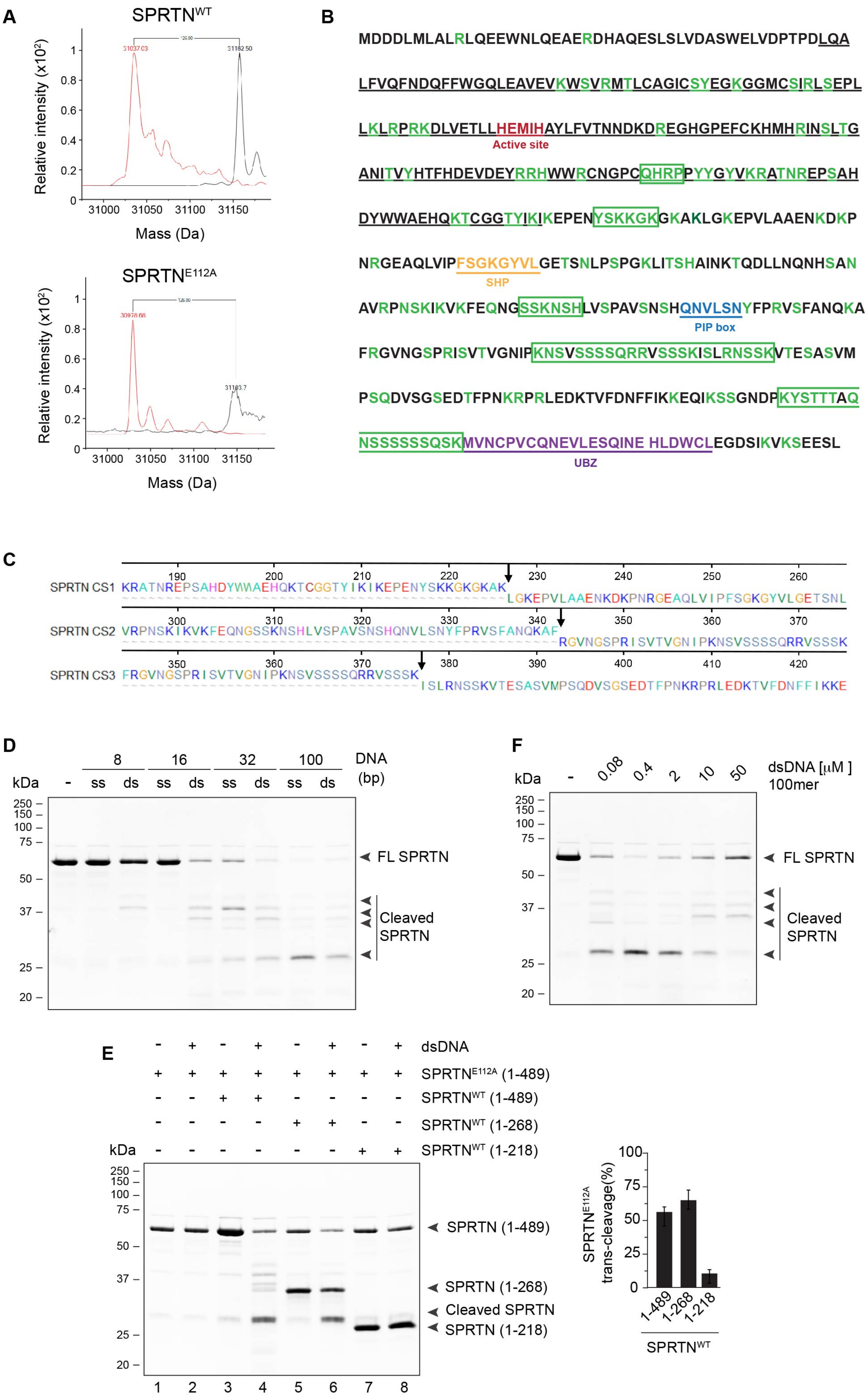

Figure S3 (referred to Figure 3)

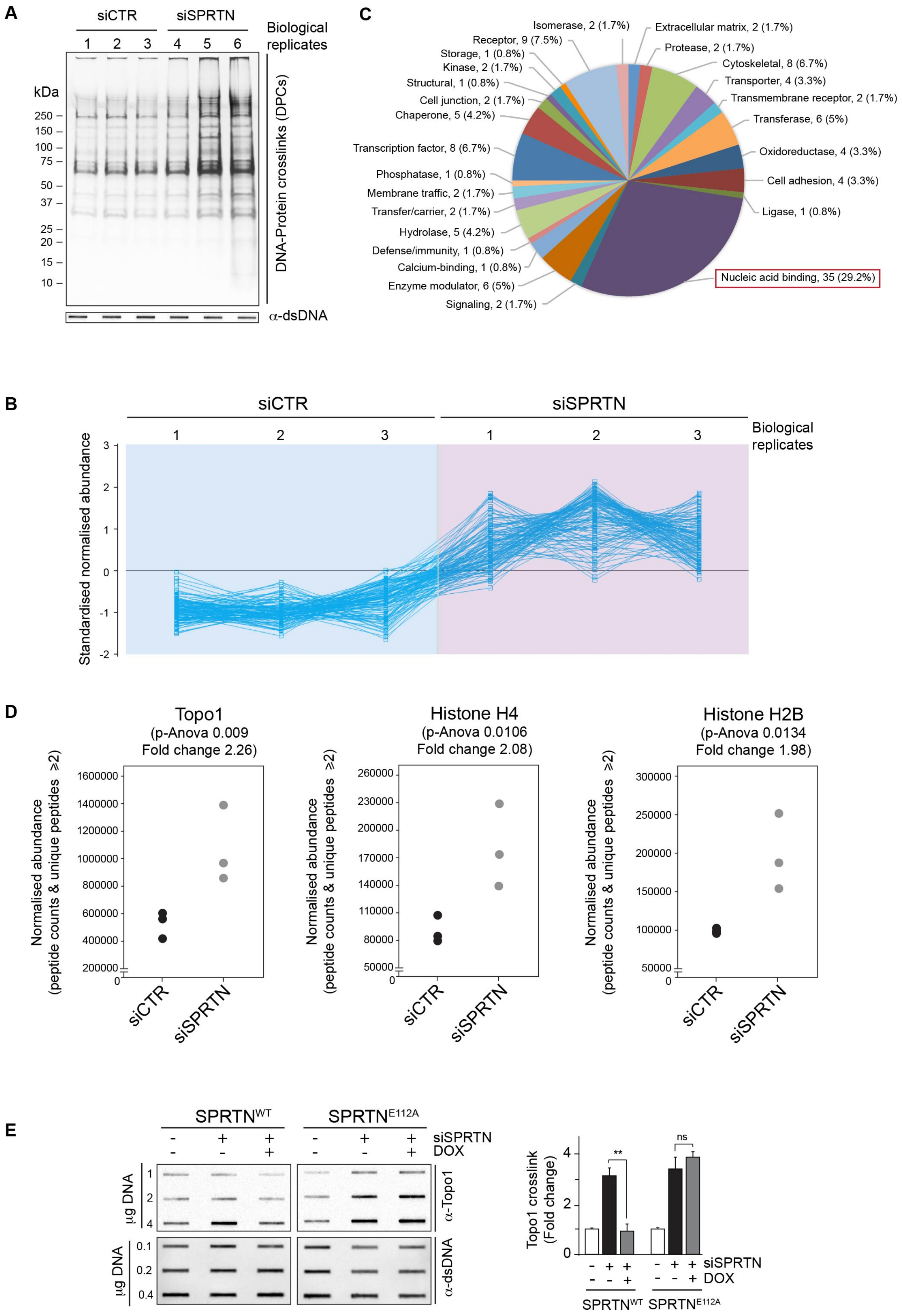

Figure S4 (referred to Figure 4)

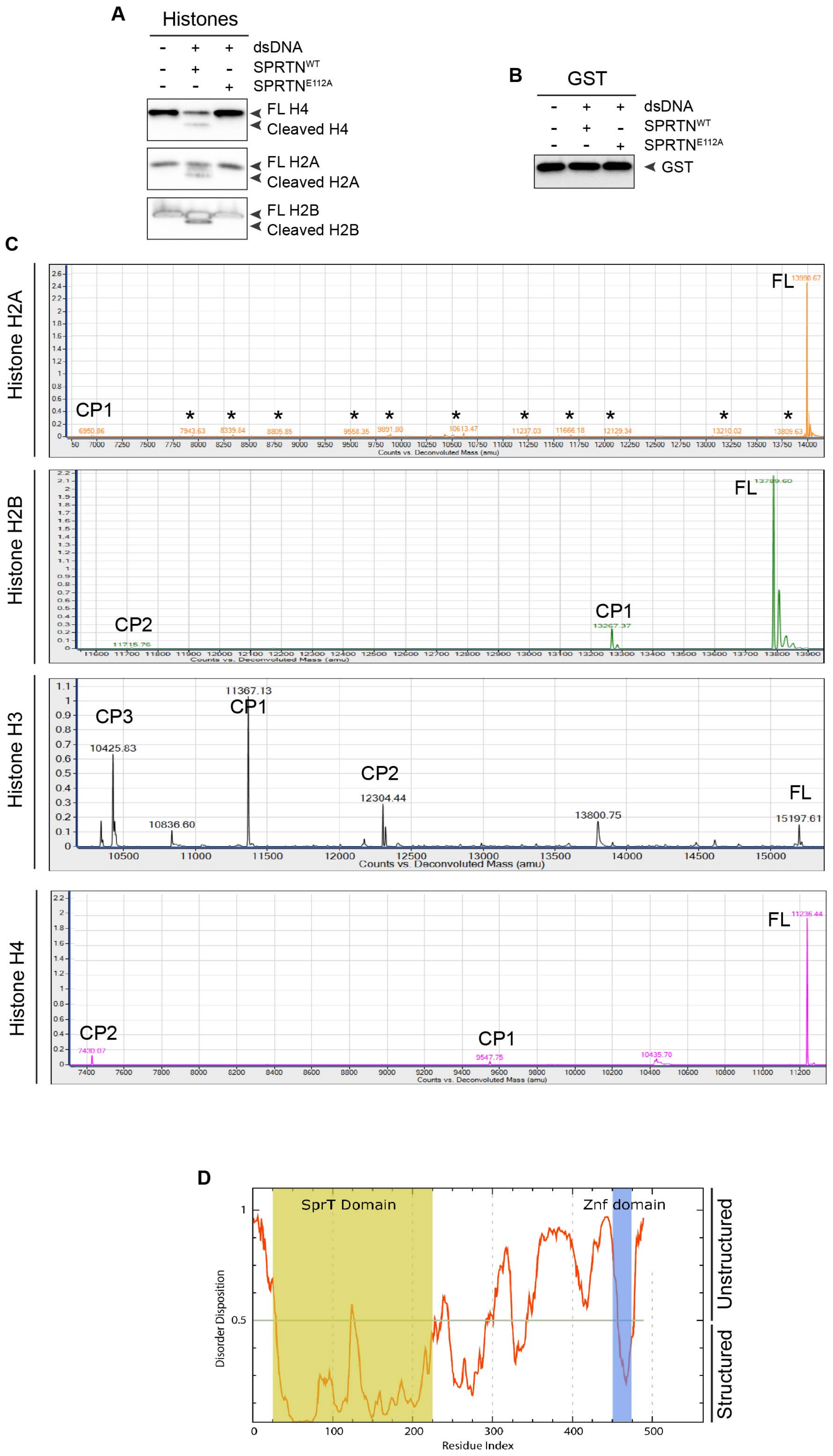

Figure S5 (referred to Figure 4)

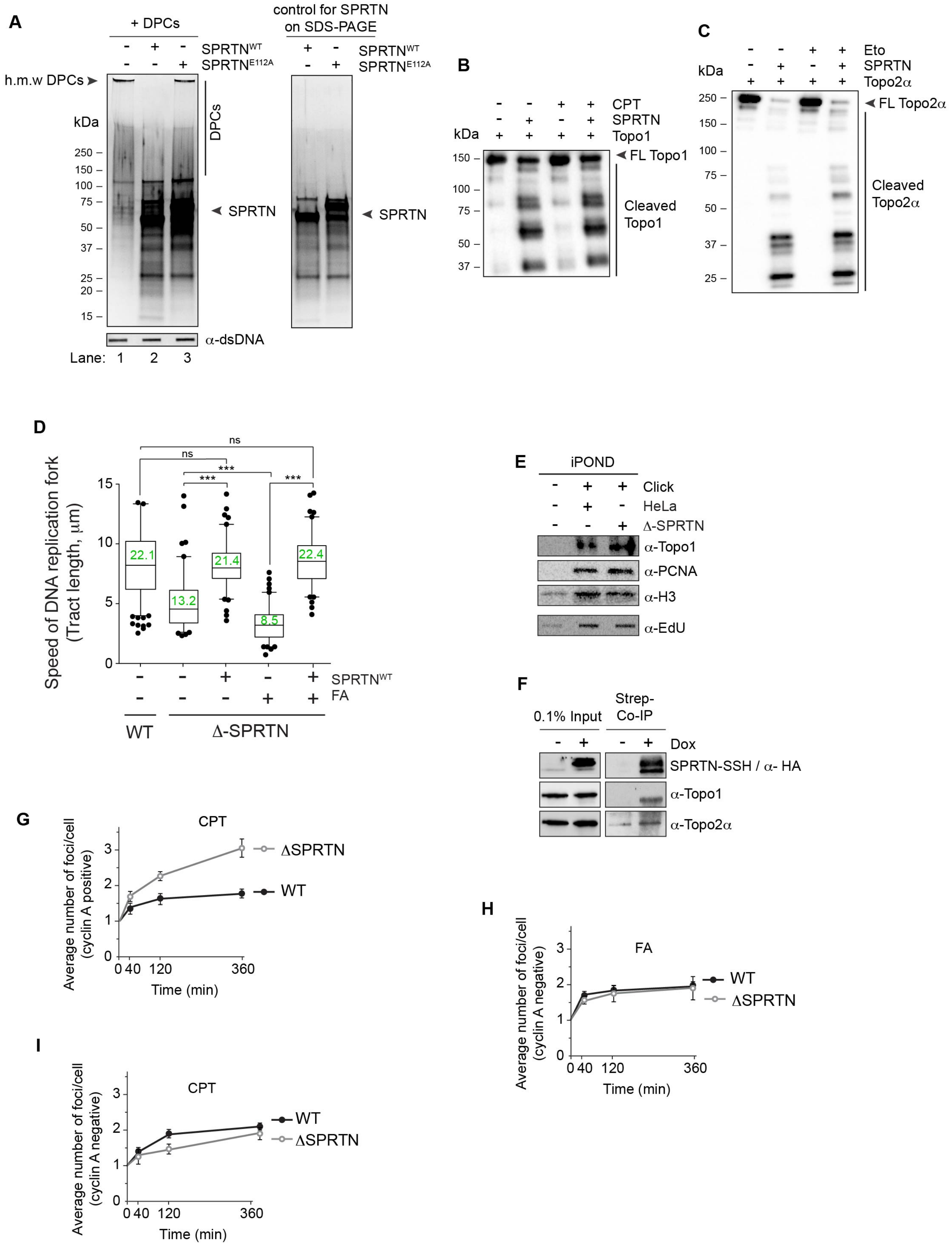

Figure S6 (referred to Figure 4 and 6)

A

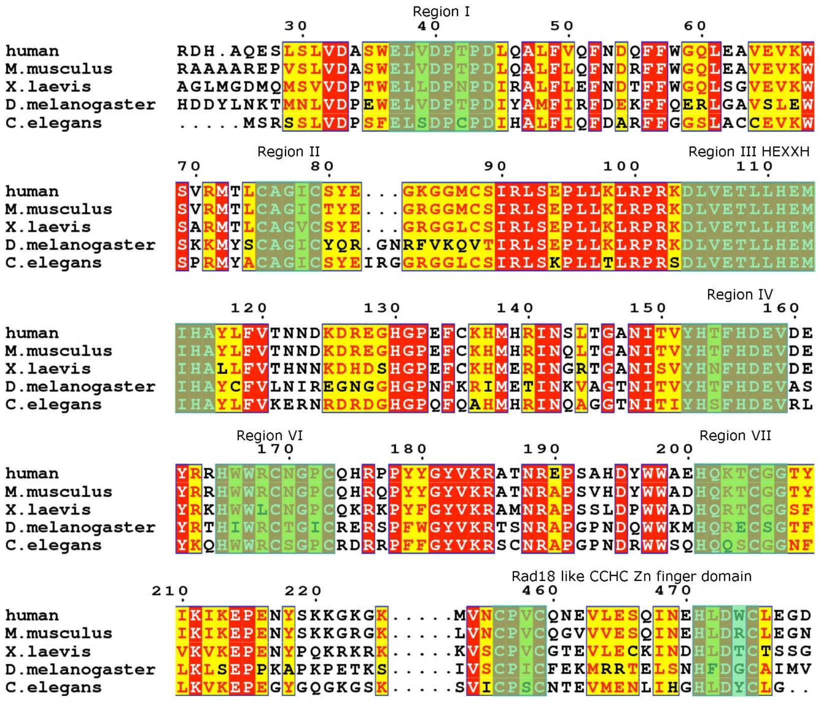

B

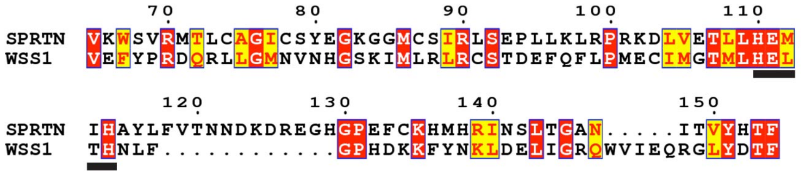

C

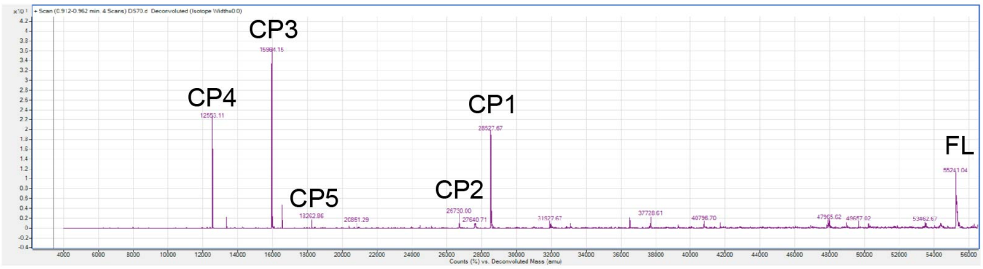

Figure S7 (referred to Figures 7 and 3)

SUPPLEMENTRY FIGURE LEGENDS

Figure S1. SPRTN prevents accumulation of DNA-protein crosslinks (DPCs). Related to Figure 1. (A) Total DPCs isolated from HEK293T cells (lane 1), and after benzonase (30 min, lane 2) or proteinase K digestion (3h, lane 3) at 55 ˚C, resolved with SDS-PAGE and visualised using silver staining. (B) Analysis of enzymatic DPCs by slot-blot; Top1-ccs after CPT treatment (upper panel) and Top2-ccs after ETO treatment (lower panel). dsDNA as a loading control for DPC isolates before benzonase treatment (C) Depletion of SPRTN in HeLa cells with three different siRNAs causes accumulation of total DPCs. Total amount of DPCs was quantified and expressed as a fold change (n = 3). Corresponding cell cycle profiles for each sample (PI; propidium iodide). WCE; whole cell extract. (D) Depletion of SPRTN in HEK293 cells with three different siRNAs leads to a significant accumulation of total DPCs as in C. Total amount of DPCs was quantified and expressed as a fold change (n = 3). WCE; whole cell extract. Cell cycle progression was monitored by propidium iodide (PI) (right panels). (E) Schematics of CRISPR/Cas9 SPRTN partial knock-out in HeLa cells (Δ-SPRTN). The sgRNA sequence targeted the first exon of *SPRTN*. (F) CRISPR partial knock-out was confirmed by western blot with α-SPRTN antibody. (G) Confirmation of SPRTN partial knock-out in HeLa cells by genomic DNA sequencing. Ten parental and 25 CRISPR clones were sequenced. The CRISPR/Cas9 plasmid induced either a 1bp deletion or 1bp insertion, leading to a frameshift and introduction of a premature stop codon. Out of 25 CRISPR clones, 17 were altered while the rest remained unchanged indicating partial knock-out of SPRTN (sequences from 10 clones that show a representative distribution of mutations are shown).

Figure S2. SPRTN protects cells from the DPC-inducing agents and acts independently of HR, NER, and Fanconi Anemia pathways in DPC removal. Related to Figure 2. (A) Total DPC levels after depletion of SPRTN, XPC, MRE11 or FANCD2 in HeLa cells visualised by silver staining with corresponding slot blots showing equal amount of DNA used for DPC analysis and Western blots for depletion efficiencies. WCE; whole cell extract. Corresponding quantification of DPC levels expressed as a fold change to DPCs levels in HeLa WT cells and cell cycle profiles (PI, propidium iodide) (right panel). (B) Clonogenic survival assays of siRNA control (CTR) or siRNA SPRTN-depleted HeLa cells treated with CPT for 24 hrs. Colonies were analysed after 7 days by automated cell counter (n = 3). (C - F) Cell survival of SPRTN depleted Flp-In HeLa cells after doxycycline-induced expression of $SPRTN^{WT}$ (C), $SPRTN^{E112A}$ (D), $SPRTN^{Y117C}$ (E), or SPRTN-ΔC (F) following CPT treatment. (G) Cell survival after SPRTN and Topo1 co-depletion following CPT treatment with corresponding WB to monitor depletion efficiency (lower panel). (H) Cell survival after SPRTN and TDP1 co-depletion following CPT treatment with corresponding WB showing depletion efficiencies (lower panel). (I) Cell survival after SPRTN and FANCD2 co-depletion following FA (upper panel) or CPT (lower panel) treatment with corresponding WB showing depletion efficiencies (right panel).

Figure S3. SPRTN is a zinc and DNA-dependent protease. Related to Figure 3. (A) Comparison of denaturing (shown in red) and native (shown in black) mass spectra for wild type and E112A SPRTN constructs (SPRTN 1-268 amino acid long truncated variant to avoid Zn-binding UBZ-domain). (B) Schematic of the SPRTN protein sequence showing the active site HEXXH (in red), p97 interacting SHP domain, PCNA binding domain (PIP box) and UBZ domain. *In silico* predicted DNA binding residues are shown in green. Four or more consecutive residues with DNA binding capacity are enclosed in green squares. (C) Identification of SPRTN self-cleavage sites. Self-cleavage sites of the SPRTN protease were identified by mass spectrometry analysis. Only cleavage sites (CS) which could be determined with high confidence (unique peptide matches, ppm = 100) are shown (CS 1-3) (See also Figure S7C). (D) Characterization of SPRTN auto-cleavage activity. *In vitro* enzymatic reactions were performed for 2 hours at 37˚C with purified SPRTN^{WT} and DNA probes of different lengths and visualized on gels by Coomassie Blue staining. (E) SPRTN self-cleavage occurs in trans. The enzymatic dead protein SPRTN^{E112A} was incubated with the full length or different C-terminal truncations of the SPRTN^{WT} protein for 2 hours at 37˚C. Trans-cleavage of SPRTN^{E112A} was quantified and expressed as a percentage of cleavage (n = 3), left panel. (F) Titration of the optimal DNA concentration to induce SPRTN self-cleavage. *In vitro* enzymatic reactions were performed with increasing concentrations of dsDNA (100mer) for 2 hours at 37˚C.

Figure S4. Quantitative mass spectrometry analysis of DNA-protein crosslinks in SPRTN-depleted cells. Related to Figure 4. (A) Total DPCs were isolated in triplicate from siRNA control (CTR) or siRNA SPRTN-depleted (siSPRTN#1) HeLa cells according to the RADAR protocol and were visualized by silver staining on SDS-PAGE gels. Samples were processed for mass spectrometry analysis. (B) Abundance profile of the 114 proteins significantly increased (p-ANOVA <0.05) in siSPRTN#1 as compared to control (n = 3 biological replicates). Proteins selected were identified and label-free quantified with at least two unique peptides using Progenesis IQP software. (C) Pie chart showing protein function classification of the 114 significantly overexpressed proteins in siSPRTN#1, which was performed using PANTHER Classification System (Protein ANalysis THrough Evolutionary Relationships, version 10.0). Proteins were identified and quantified with at least two unique peptides. (D) Increased DNA topoisomerase 1, Histone H4 and Histone H2B association to DNA in SPRTN#1 depleted cells. Normalised abundance of three proteins differentially increased in siSPRTN#1 depleted cells as compared to control (fold change ≥ 1.5; p-ANOVA <0.05). (E) Total DPCs were isolated by the RADAR protocol as in Figure 1A from stable Flip/In HeLa cell lines depleted for endogenous SPRTN by 3' UTR siRNA. Ectopic expression of SPRTN^{WT} or SPRTN^{E112A} was induced by DOX and Topo1 levels in DPCs were analyzed by slot blot using a specific antibody against Topo1. Equal amount of DNA was loaded and confirmed by slot blot followed by immune-detection against dsDNA (lower panel). Total amount of Topo1 was quantified and expressed as a fold change compared to siCTR cells (n = 3, right panel).

Figure S5. SPRTN cleaves histones H2A, H2B and H4. Related to Figure 4. (A) SPRTN^{WT} cleaves core histones *in vitro*. Enzymatic reactions were performed with purified SPRTN proteins as indicated and different histones in the presence of dsDNA for 8 hrs at 37˚C. Proteolytic activity was monitored by Western blot using antibodies against the specific histones. (B) Cytosolic protein glutathione S-transferase (GST) is not cleaved by SPRTN. Enzymatic reactions were performed as described above. (C) Mass spectrometry analysis of histones H2A, H2B, H3 and H4 cleavage products (CP) after SPRTN proteolysis. * denotes unspecific peaks. Peaks that are not labelled as CP had more than one unique peptide hit, thus making it impossible to determine exact cleavage site (see Methods) (D) Disorder propensity plot for human SPRTN with the known domain boundaries overlaid. Values greater than 0.5 are considered to be likely disordered.

Figure S6. SPRTN cleaves Topo1-ccs, Topo2α-ccs, and DPCs of high molecular weight and prevents protein accumulation at the replisome and DSB formation. Related to Figure 6. (A) Left gel: *In vitro* enzymatic reactions of SPRTN cleavage of DPCs. Incubation of DPCs isolated from HeLa cells with SPRTN^{WT}, but not SPRTN^{E112A}, leads to a reduction of high molecular weight (h.m.w.) DPCs, and DPCs above 100 kDa (lane 2 in comparison to lanes 1 and 3). Right gel: analysis of SPRTN WT or E112A on SDS-PAGE/Silver staining to demonstrate that products in lanes 2 and 3 are coming from recombinant SPRTN in the reaction. (B) SPRTN cleaves Topo1 and Topo1-ccs with similar efficiency. Topo1 or Topo1-ccs (+CPT) were immunoprecipitated under denaturing conditions from chromatin fraction of HEK293T cells before or after CPT treatment and in vitro enzymatic reactions were performed as in Figure 4F. (C) SPRTN cleaves Topo2α and Topo2α-ccs (+ETO) with similar efficiency. Topo2α and Topo2α-ccs were immunoprecipitated under denaturing conditions from chromatin fraction of HEK293T cells before or after ETO treatment and in vitro enzymatic reactions were performed as in Figure 4F. (D) Ectopic expression of SPRTN^{WT} rescues DNA replication fork velocity in Δ-SPRTN cells before and after FA treatment (n = 3). Numbers in green indicates mean value of DNA synthesis in kilobases (E) Topo1 protein levels increase at the replisome in Δ-SPRTN cells. iPOND was performed as in Figure 6B. (F) SPRTN forms a complex with Topo1 and Topo2α *in vivo*. Co-IP of SPRTN-SSH tag was performed upon doxycycline induction of SPRTN^{WT} expression in Flp-In 293 cells using StrepTactin beads. (G) Graphic representation of the fold-change in the average 53BP1 foci following treatment with camptothecin (25 nM) in HeLa WT or Δ-SPRTN cells. DSB formation was monitored in cyclin A positive cells, as described in Figure 6F. (H and I) Graphic representation of the fold-change in the average 53BP1 foci following treatment with FA (50 μM, H) or CPT (25 nM, I), respectively in HeLa WT or Δ -SPRTN negative for cyclin A.

Figure S7. SPRTN protein alignment and mass spectrometry analysis of SPRTN auto-cleavage (A) Multiple sequence alignment of SPRTN homologues from a diverse set of multicellular eukaryotes. Regions with dense clusters of strictly conserved residues, which also contain possible consensus motifs for metal binding, are numbered and highlighted in green (I-VII). (B) Pairwise

sequence alignment of human SPRTN and *S. cerevisiae* WSS1 shows low similarities over a short region of the catalytic domain around the HEXXH motif, underlined in black. (C) Mass spectrometry analysis of full length SPRTN-WT auto-cleavage products (CP) identified at least five cleaved products within the C-terminal region of SPRTN.

EXTENDED EXPERIMENTAL PROCEDURES

Chemicals, plasmids and recombinant proteins

Formaldehyde (FA), Camptothecin (CPT), Etoposide (Eto), methylglyoxal, hygromycin, puromycin, 5-Chloro-2′ deoxyuridine (CldU) and 5-Iodo-2′-deoxyuridine (IdU) were purchased from Sigma-Aldrich. Topo1/YFP and Topo2α/GFP plasmid constructs were a kind gift from Prof Sherif El-Khamisy. pcDNA3.1/Flag (Invitrogen) and pCDNA5/FRT/TO-cSSH (Invitrogen) were used in the mammalian expression system and pNIC-ZB vector and pNIC28-Bsa4 were used in the E.coli expression system.

Antibodies

The following primary antibodies were used in this study: anti-SPRTN (rabbit, polyclonal) raised against the N-terminal part (1–240 aa) of SPRTN (home made, dilution 1:1000); anti-SPRTN raised against the C-terminal part of SPRTN (Atlas HPA 025073, dilution 1:1000); anti-Topoisomerase 1 (Bethyl A302-589A, dilution 1:5000); anti–topoisomerase 2α (Bethyl A300-054A, dilution 1:10000); anti-dsDNA (Abcam ab27156, dilution 1:5000); anti-histone 2A (Cell signalling 2578, dilution 1:1000); anti-histone 2B (Cell signalling 2934, dilution 1:1000); anti-histone 3 (Abcam ab1791, dilution 1:1000); anti-histone 4 (dilution 1:1000); anti-PCNA (Abcam ab29, dilution 1:1000); anti-MCM6 (Santa Cruz sc-9843, dilution 1:1000); anti-MCM2 (Cell signalling 4007, dilution 1:1000); anti-Polδ (Abcam ab10362, dilution 1:1000); anti-βActin (abcam ab6276, dilution 1:1000); anti-Lamin B1 (ThermoFisher Scientific PA5-19468, dilution 1:1000); anti-cyclin A (Santa Cruz sc751, dilution 1:1000); anti-cyclin B1 (BD Biosciences 610219, dilution 1:1000); anti-cyclin E (Millipore 05-363, dilution 1:1000); anti-DNA-PK (Cell signalling 12311S, dilution 1:1000) and anti-rat and anti-mouse 5-bromo-2′-deoxyuridine (BrdU) (anti mouse Abcam ab6326, dilution 1:500, and anti rat BD Biosciences 347580, dilution 1:100, respectively). Secondary antibodies used in this study are as follows: anti-mouse horseradish peroxidase (HRP) (Sigma A2304, dilution 1:50000); anti-rabbit HRP (Sigma A05451, dilution 1:50000); Anti-rat Cy3 (Jackson ImmunoResearch 712-116-153, dilution 1:300) and anti-mouse Alexa-488 (Molecular Probes A11001, dilution 1:300).

Cloning and site directed mutagenesis

The I.M.A.G.E. full-length *SPRTN* cDNA clone (IRATp970E1156D, ImaGenes) was cloned into pcDNA3.1 (containing the Flag-tag at the N-terminus) and pCDNA5/FRT/TO-cSSH vector (containing Strep and HA tags at the C-terminus) for expression in a mammalian system. For expression in E. coli, *SPRTN* cDNA clone was cloned into pNIC-ZB vector with N-terminal His and ZB tags (full length $SPRTN^{WT}$, $SPRTN^{E112A}$, and $SPRTN^{Y117C}$) or pNIC28-Bsa4 vector with N-terminal His tag (all other truncated constructs). Site-directed mutagenesis was performed using

mutagenic primers by PCR using AccuPrime Pfx DNA polymerase (Invitrogen) according to the manufacturer instructions. DNA sequence was verified by Source Biosciences sequencing service, Oxford, UK.

Mammalian cells, siRNA and transfection protocols

HeLa, HEK293T and T24 cells were cultured in Dulbecco's modified Eagle's medium with 10% fetal calf serum. Epstein-Barr virus (EBV)-transformed LCLs were cultured in RPMI medium with 15% fetal calf serum. Cells were grown to 50–80% confluence before treatment with CPT, ETO, FA or methylglyoxal at indicated concentrations. siRNA transfections were performed using Lipofectamine RNAiMax reagent (Invitrogen) according to the manufacturer's protocol, and depletion was assayed at 72 h post-transfection. siRNA sequences are as follows:

siSPRTN#1 (GUCAGGAAGUUCUGGUUAA);

siSPRTN#2 (CACGAUGAGGUGGAUGAGUAU);

siSPRTN#3 (AGCCAAUAUAACGGUAUACCA);

siTopo1 (GGUCCCUGUUGAGAAACGA);

siTDP1 (GGAUAUUGCGUUUGGAACA);

siMre11 (GAUAGACAUUAGUCCGGUU)

siXPC (SMARTpool, 40nM) (Dharmacon), and

siFANCD2 (SMARTpool, 40nM) (Dharmacon).

Plasmid transfections were performed using FuGene HD reagent (Promega) according to the manufacturer's protocol, and drug response was assayed at 24-48 h post-transfection. For denaturing immunoprecipitation (IP) of Topo1/YFP and Topo2α/GFP, HEK293T cells were transfected with corresponding recombinant plasmid using polyethyleneimine (PEI) reagent (Tom et al., 2008) and collected for IP after 48h.

Generation of SPRTN/Flp-In T-REx stable cell lines

HeLa and HEK-293 SPRTN/Flp-In T-REx stable cell lines were prepared according to manufacturer's protocol for doxycycline inducible constitutive expression of SPRTN variants (WT, E112A, Y117C and DC). The *SPRTN* cDNA was amplified by PCR and cloned in pCDNA5/FRT/TO-cSSH vector (containing Strep and HA tags at C-terminal) using BamH1 and Not1 restriction sites to generate DVC1-WT-FRT/TO construct. The construct was used as a template to generate the E112A, Y117C and DC variants by site directed mutagenesis. The DVC1-wt and DVC1-mutation-FRT/TO constructs were transfected with pOG44 vector into Flip-In host cell lines for site-specific integration in genome. The transformed cells were selected under hygromycin B to generate DVC1-Flp-In T-REx stable cell lines. The expression of DVC1 was confirmed by doxycycline induction (1μg/ml) followed by western blot analysis.

Generation of Crispr/Cas9 SPRTN partial knock-out HeLa cells (Δ-SPRTN)

The CRISPR plasmid was obtained from the Genome Engineering Oxford (GEO) centre, Sir William Dunn School of Pathology, Oxford, UK. The donor plasmid (pX459v2), containing the sgRNA, Cas9 and puromycin resistance marker, was transfected into HeLa cells using Fugene HD (Promega). The sgRNA sequence (CACGCTCCACTTCACCTCGACGG) targeted the first exon of *SPRTN*. 24h after transfection, cells were selected with 0.6 µg/ml puromycin for 72h and then seeded as single cells in a 96-well plate (one cell/well) in order to generate a population of cells derived from a single clone (i.e. a genetically homogenous population of cells). In parallel, 40 distinct CRISPR-targeted HeLa cell clones (each grown from a single cell) were tested for SPRTN protein expression by Western blotting. Only those HeLa clones, which showed a reduction in SPRTN protein levels, were selected for sequencing. Genomic DNA was isolated from each individual pool of genetically-homogenous HeLa cells and the region containing the SPRTN sgRNA target site was amplified by PCR and subcloned into Topo vectors to achieve allele separation and transformed into bacteria. Plasmid was then isolated from 25 bacterial colonies and sequenced. The clones in Figure S1 refer to plasmids isolated from individual bacterial colonies derived from HeLa cells grown from a single clone. 70% (i.e. 17/25 or 2/3) of the sequences showed Cas9-mediated mutations in exon 1 of *SPRTN*. Knowing that HeLa cells (cancer cell line) have three copies of chromosome 1, where the *SPRTN* gene is located, this result suggests that 2 out of 3 alleles of the SPRTN gene have been knocked-out. Therefore we called our SPRTN-knock out cells "partial knock-out" as 2 alleles are knocked-out and 1 allele is still present.

Western Blot (WB)

Standard protocols for sodium dodecyl sulfate-polyacrylamide gel electrophoresis (SDS PAGE) and immunoblotting were used (Henderson and Wolf, 1992). Nitrocellulose membrane (GEHealthcare) or PVDF (BioRad) were used to transfer proteins from polyacrylamide gels depending on the antibody.

DNA-protein crosslinks isolation

DPCs were detected using a modified rapid approach to DNA adduct recovery (RADAR) assay (Kiianitsa and Maizels, 2013). In brief, 1.5 to 2 x 10^6 cells were lysed in 1 ml of M buffer (MB), containing 6 M GTC, 10 mM Tris–HCl (pH 6.8), 20 mM EDTA, 4% Triton X100, 1% Sarkosyl and 1% dithiothreitol. DNA was precipitated by adding 1 ml of 100% ethanol and was washed three times in wash buffer (20 mM Tris–HCl pH 6.8, 150 mM NaCl and 50% ethanol) and DNA was solubilized in 1 ml of 8 mM NaOH. A small aliquot of the recovered DNA was digested with 50 µg/ml proteinase K (Invitrogen) for 3 hours at 50°C and quantified using PicoGreen dye (Invitrogen) according to manufacturer instructions to determine DNA concentration. DNA concentration was further confirmed by slot-blot analysis followed by immunodetection with antibody against dsDNA.

As confirmation of results obtained by RADAR assay for DPC isolation, DPCs were isolated using

KCl/SDS precipitation assay (Zhitkovich and Costa, 1992). In brief, approximately $2x10^6$ cells were lysed in 1 ml denaturing lysis buffer (2 % SDS, 20 mM Tris/HCl pH 7.5) followed by sonication (5 cycles, 20 sec). Proteins were then precipitated in buffer containing 200 mM KCl, 20 mM Tris, pH 7.5 (assay buffer) and incubated on ice for 5 min. The precipitate was pelleted by centrifugation at 4°C (15,000g, 5min). Supernatant was used for quantifying soluble DNA. The pellet was resuspended in 1ml assay buffer and incubated at 55°C for 5 min, cooled on ice for 5 min, and precipitated by centrifugation (15,000g, 5min). Pellet was washed three times in assay buffer prior to final resuspension in 500 µl of assay buffer. Proteins were digested with 0.2 mg/ml Proteinase K (55°C, 3 hours). Samples were cooled on ice for 5 min then centrifuged. The final supernatant contained the crosslinked DNA. Soluble and crosslinked DNA were quantified by PicoGreen. The amount of DPCs was calculated as the ratio between DNA precipitated by SDS/KCl and total DNA (SDS/KCl precipitated plus soluble DNA).

DNA-protein crosslinks detection

Total DPCs were visualized by silver staining (Sigma) as recommended by the manufacturer after electrophoretic separation on polyacrylamide gels. DNA was digested with benzonase (Invitrogen) for 30 minutes at 37°C. Proteins were precipitated by standard Trichloroacetic Acid (TCA) protocol (Link and LaBaer, 2011) and resolved by SDS-PAGE gel. Specific DPCs were detected using a vacuum slot-blot manifold (Bio-Rad) followed by immunodetection. In brief, equal amounts of DNA were diluted in Tris-buffered saline (TBS) and applied to either a polyvinylidene difluoride (PVDF, Millipore) or nitrocellulose (Bio-Rad, Hercules CA) membrane using a vacuum slot-blot manifold. The membrane was then blocked in 3%BSA in TBST (TBS containing 0.1% Tween 20), incubated with primary antibodies followed by incubation with horseradish peroxidase-conjugated secondary antibodies. DPCs were visualized using the Bio-Rad ChemiDoc XRS Plus Analyzer. Sample loading was further confirmed by slot-blot detection of dsDNA with a specific antibody α-dsDNA. For the dsDNA detection samples were digested with proteinase K, diluted in Tris/Borate/EDTA (TBE) buffer, and applied to nylon membrane (Hybond N+).

Colony forming assay

Cell survival after exposure to CPT, Eto, FA or methylglyoxal was determined by standard clonogenic assay. In brief, cells were seeded in 6-well plates and incubated overnight. Cells were exposed to increase doses of the drugs diluted in DMEM, washed and incubated again in fresh medium. Cells were exposed for 24 hours with CPT and methylglyoxal, 1 hour with Eto and 20 minutes with FA. Colonies were fixed 7-10 days later, and the number of clones was counted using the automated colony counter GelCount™ (DTI-Biotech). The number of colonies in treated samples was expressed as a percentage of colony numbers in the untreated samples.

Cell viability assay

Cell viability assay was performed with resazurin dye according to the manufacturer's instructions (Cell signalling).

Cell cycle analysis

For flow cytometry analysis, 0.2 x 10^6 – 0.5 x 10^6 cells were harvested by centrifugation and fixed in ice-cold methanol, followed by resuspension in Phosphate-buffered saline (PBS) containing 1% Bovine serum albumin (BSA), 20 µg/ml of propidium iodide or 10 µg/ml DAPI and 10 µg/ml of RNase A. Flow cytometry was performed on a FACScalibur instrument (BD Biosciences). Cell-cycle phase distributions were analyzed using FlowJo software. Please describe here in brief or cite a paper for thymidine block and release.

Cell cycle synchronization

HeLa cells were synchronized at G1/S of the cell cycle by double thymidine treatment, as described previously(Harper, 2005). T24 cells were synchronized as described previously (Jin et al., 1997).

Protein purification

For overexpression in *E.coli* cells SPRTN constructs were cloned in to either the pNIC-ZB vector (full length SPRTN-WT, SPRTN-E112A, and SPRTN-Y117C), or pNIC28-Bsa4 vector (all other truncated constructs). For purification of full length constructs containing a TEV cleavable Z-basic-his tag, cell pellets were thawed and resuspended in lysis buffer (100 mM HEPES pH 7.5, 500 mM NaCl, 10% glycerol, 10 mM imidazole, 1 mM Tris (2-carboxyethyl) phosphene (TCEP), 0.1% DDM, 1 mM MgCl, 1 x set III protease inhibitors (Merck). Cells were lysed by sonication and 1 unit of Benzonase was added to lysates before the cell were debris pelleted by centrifugation. Lysates were applied to a Ni-sepharose IMAC gravity flow column, washed with 2 column volumes of wash buffer (50 mM HEPES pH 7.5, 500 mM NaCl, 10% glycerol, 45 mM imidazole, 1 mM TCEP), and eluted in elution buffer (50 mM HEPES pH 7.5, 500 mM NaCl, 10% glycerol, 300 mM imidazole, 1 mM TCEP). Elution fractions were applied directly to a 5ml Hitrap SP HP column (GE healthcare), washed with wash buffer (50 mM HEPES pH 7.5, 500 mM NaCl, 1 mM TCEP) and eluted with elution buffer (50 mM HEPES pH 7.5, 1M NaCl, 1 mM TCEP). The purification tag was cleaved with the addition of 1:20 mass ratio of His-tagged TEV protease during overnight dialysis into buffer A (20 mM HEPES, pH 7.5, 500 mM NaCl, 0.5 mM TCEP). Samples were concentrated by ultrafiltration using a 30 kDa molecular weight cut off centrifugal concentrator and loaded on to size exclusion chromatography using a HiLoad 16/60 Superdex 200 column at 1 ml/min in buffer A. The same protocol was used for purification of the truncated constructs with the exception of the omission of the SP HP column and the inclusion of a Ni-sepharose rebind following TEV cleavage (to remove his-

tagged TEV protease). Protein identities were verified by LC/ESI-TOF Mass spectrometry and protein concentrations were determined by absorbance at 280nm (Nanodrop) using the calculated molecular mass and extinction coefficients.

Fluorescence polarisation DNA binding assays and DNA probe annealing

DNA binding was measured using a fluorescence polarisation based assay. DNA oligonucleotides with the sequences as follows

OD1 - ATC GAT AGT CGG ATC CTC TAG ACA GCT CCA TGT AGC AAG GCA CTG GTA GAA TTC GGC AGC GTC,

OD2 - GAC GCT GCC GAA TTC TAC CAG TGC CTT GCT ACA TGG AGC TGT CTA GAG GAT CCG ACT ATC GAT, and

OD3 - GAC GCT GCC GAA TTC TAC CAG TGC CTT GCT AGG ACA TCT TTG CCC ACC TGC AGG TTC ACC C were mixed together at a 10 μM concentration in a buffer consisting of 10 mM HEPES pH 7.5, 50 mM NaCl in the following combinations, single stranded (ss) =OD1, double stranded (ds) OD1+OD2 and splayed duplex OD1+OD3. For all substrates the OD1 oligo was labelled on the 5' end with Fluorescein isothiocyanate and substrates were formed by heating 96°C and allowing to cool on a heat block over 2 hrs. Probes were used at a final concentration of 10 nM and binding experiments were performed in a buffer containing 10 mM HEPES pH 7.5, 150 mM NaCl, with varying protein concentration. Measurements were performed in 384 well plates (30 μl final volume) in a POLARstar plate reader (BMG Labtech) with excitation at 485 nm and emission at 520 nm. Kinetic constants were calculated from binding curves using a 4 parameter logarithmic binding equation using the program PRISM (GraphPad).

***In vitro* self –cleavage assays**

SPRTN self-cleavage assays were performed in 20 μl reaction volume, containing 2 μl SPRTN (1 mg/ml), 10 μl of reaction buffer (25 mM Tris pH 7.5, 150 mM NaCl), 6 μl H2O, and 1 μl DNA (10 μM). Several types of DNA probes were used for induction of cleavage, ssDNA and dsDNA of various lengths: 100, 32, 16, and 8 bp. Samples were incubated for 2 hours at 37°C, and stopped by the addition of 2X Laemmli buffer and resolved by SDS-PAGE followed by coomassie blue staining.

***In vitro* cleavage of the SPRTN substrates**

SPRTN enzymatic reactions with histones were performed in 150 mM NaCl, 25 mM Tris pH 7.4 in a PCR block at 37°C. The reaction volume was typically 10 μl and contained: E. coli purified recombinant SPRTN (1 – 10 mg/ml solution), substrate (typically 1 mg/ml solution) and 100 bp long dsDNA oligonucleotide probe (**OD4** ACGCGGGTTAGCGGTACCCAGTCCAGTGACCTAGGCAGCTTTAAGCTAGTACGACTTGCT TAGATTGCAGTCGACGACGTAGCTGGCATAGAGGTACAGC) (40 μM stock solution) in

Klenow buffer without DTT. Conditions for cleavage reactions of histones were performed with the 3:1 molar ratio of SPRTN:substrate and 15:1 molar ratio of SPRTN: dsDNA in a 10µl volume. The cleavage reactions for Topo1 and Topo2α were performed in 16 µl volume and 30:1 molar ratio of SPRTN : dsDNA. Reactions were incubated for 20h at 37˚C and stopped by the addition of 2X Laemmli buffer. Reactions were resolved by SDS-PAGE followed by western blotting with substrate specific antibody. The enzyme (SPRTN) loading was monitored with α-SPRTN (N-terminal) in each reaction mixture.

***In vitro* DPCs cleavage**

DPCs were prepared according to the SDS/KCl precipitation assasy with the following modifications. Samples were washed in buffer containing 200 mM KCl, 20 mM Tris (pH 7.5), and DPCs were separated from total proteins using the QIAquick PCR Purification Kit (Qiagen). I*n vitro* DPC cleavage was performed in 150 mM NaCl, 25 mM Tris pH 7.4 in a PCR block at 37°C for 3 hours. The reaction volume was typically 20 µl and contained 10 µg of DPCs and 1 µg of recombinant SPRTN protein. Reactions were stopped by the addition of 2X Laemmli buffer, resolved by SDS-PAGE and visualized by silver staining.

Immunoprecipitation

To isolate Topo1 and Topo2α, HEK293T cells were transiently transfected with GFP-Topo2α or YFP-Topo1 using PEI (polyethyleneimine) reagent (Tom et al., 2008). Both proteins were immunoprecipitated using GFP-trap beads (Chromotek) in denaturing conditions (1% SDS, 5mM EDTA). Cell lysates were digested overnight with benzonase nuclease (Sigma) to further solubilize the sample. Sonication was avoided due to the fact that it disrupts binding of YFP-Topo1 or GFP-Topo2α to the GFP beads. Samples were diluted ten times in the IP buffer (150 mM NaCl, 10 mM Tris, 0.5 mM EDTA pH 7.5) with 1% Triton and precleared with bab-20 beads (Chromotek) for 30 min at 4°C. After discarding the bab-20 beads (50 G, 30 sec centrifugation), precleared samples were incubated with GFP beads (Chromotek) for 4h at 4°C, followed by five washes in the IP buffer. Cells were treated with 10 µM CPT or 25 µM ETO for 1h, when indicated.

Co-immunoprecipitation

To isolate SPRTN-interacting proteins, lysates from Flp-In 293 cells upon doxycycline induction of SPRTN-WT-SSH (Strep-Strep-HA tag) were prepared. Samples were lysed in 1% Triton, 150 mM NaCl, 50 mM Tris, 1mM EDTA pH 7.4 for 1h at 4°C with 10 mM NEM and protease and phosphatase inhibitor cocktails (ThermoFisher Scientific) and avidin (IBA), followed by centrifugation at 16,000 g for 10 min. The pellet was digested with nuclease benzonase in the bensonase buffer (Invitrogen) including protease and phosphatase inhibitor cocktail, overnight at 4°C. Both fractions were pooled and diluted 10 times in IP buffer (150 mM NaCl, 50 mM Tris, pH 8)

followed by preclearing of the lysate with blank sepharose (IBA) for 1h at 4°C. Samples were incubated with Strep-Tactin sepharose (IBA) for 2h at 4°C, washed 5 times in IP buffer containing 0.05% NP-40 and eluted in 2X Laemmli for 10 min at 95°C.

Cellular fractionation

Cellular fractionation was performed as previously described (Mendez and Stillman, 2000) with the slight modifications as follows: after isolation of cytosolic and nuclear soluble fractions, the chromatin fraction was digested with benzonase 1µl of benzonase (200u/ml) in a buffer containing 50 mM Tris, pH 7.9, 50 mM NaCl and 5 mM KCl overnight at 4˚C with gentle agitation.

Isolation of Proteins On Nascent DNA (iPOND)

iPOND was performed as described in (Sirbu et al., 2012) with the following modifications. Newly synthesized DNA in HEK293 or HeLa wild-type or SPRTN-knockout cells (~2 x 10^8 cells per condition) was labelled via incubation with 10 µM Edu for 10 and 15 minutes, respectively. For thymidine chases, cell culture media was supplemented with 10 µM thymidine and incubated for 2 or 10 minutes as indicated. HEK293 cells were synchronised by double thymidine block, and washed thoroughly with 1 x PBS followed by cell culture media and released into S-phase for 4 hours before EdU labelling. For experiments in HEK293 cells, chromatin was fragmented into 50-300 bp fragments by sonication with a Bioruptor Plus sonicator (30 seconds ON, 30 seconds OFF for 50 cycles). In HeLa cells, chromatin was fragmented into 400 bp fragments (30 seconds ON, 30 seconds OFF for 5 cycles). To isolate proteins on Edu-labelled DNA, samples from incubated overnight with streptavidin-coupled agarose beads (Merck Millipore). Aliquots of each extract were kept for loading controls.

DNA fiber assay

The DNA fiber assay was performed as described previously (Lessel et al., 2014). Briefly, asynchronous LCL or HeLa cells were labelled with 30 µM of CldU (Sigma, C6891) for 30 min, and then labelled with 250 µM of IdU (Sigma, 17125) for an additional 30 min. HeLa cells were washed 3 times with warm PBS after 1st nucleotide (CldU) incubation. DNA replication was inhibited by treating cells with ice-cold PBS. Cells were lysed in 200 mM Tris-HCl pH 7.4, 50 mM EDTA and 0.5% SDS, DNA fibers were spread onto glass slides, fixed with 3:1 methanol and acetic acid, denatured with 2.5 M HCl, blocked with 2% BSA and stained with anti-rat and anti-mouse 5-bromo-2′-deoxyuridine (BrdU) that specifically recognize either CldU (Abcam-Ab6326, dilution 1:500) or IdU (BD- 347580, dilution 1:100). Anti-rat Cy3 (dilution 1:300, Jackson Immuno Research, 712-116-153) and anti-mouse Alexa-488 (dilution 1:300, Molecular Probes, A11001) were used as the respective secondary antibodies. Microscopy was done using a Leica DMRB microscope with a

DFC360FX camera. The lengths of the CldU- and IdU-labelled tracts were measured by ImageJ software and converted into micron scale. Statistical analysis was done by GraphPad Prism software using unpaired *t*-test. Mean value was then converted into kilo base (1µm = 2.59 kb) to precisely determine the replication fork speed as shown previously (Petermann et al., 2010). For the DNA fiber assay under genotoxic stress, the second nucleotide (IdU) was either mock treated or incubated in the presence of 25 nM CPT or 50 µM formaldehyde (30 min).

Mass spectrometry

Mass Spectrometry of Intact Proteins

For mass spectrometry under denaturing conditions 50-µl protein samples were injected at ~0.02 mg/ml in 0.1% formic acid onto a 2.1 mm x 12.5 mm Zorbax 5 µm 300SB-C3 guard column (Agilent) resolved by reversed-phase chromatography at 40 °C. The solvent system was 0.1% formic acid in LC-MS grade water (buffer A) and 0.1% formic acid in LC-MS grade methanol (buffer B), and proteins were eluted with a linear gradient of 5–95% buffer B over 1.5 min at 1.0 ml/min. Protein masses were determined using an Agilent 6530 QTOF. The analysis of MS spectra was performed in ICP-MS MassHunter Software (Agilent). Analysis of histone and SPRTN peptides was done using Protein Analysis Worksheet (PAWS). Only cleavage products (CPs) which could be determined with high confidence (unique peptide matches, ppm 50) are taken into consideration for the analysis of SPRTN cleavage site. Histone protein sequences H2A (AAN59960.1), H2B (AAN59961.1), H3 (NP_002098.1) and H4 (NP_778224) without initial Methionine residue corresponded to the recombinant protein molar mass as stated by the manufacturer (NEB). For mass spectrometry under native conditions protein samples, 50 µl at 1 mg/ml were buffer exchanged into a buffer consisting of 50 mM ammonium acetate pH 6.5, and injected directly at a flow rate of 6 µl per minute. Protein masses were determined using an Agilent 6530 QTOF.

Mass spectrometry for the identification of proteins crosslinked to the DNA

DPCs were isolated as described previously. Equal amounts of DNA were digested with benzonase (Invitrogen) for 60 minutes at 37°C. Proteins were precipitated by standard Trichloroacetic Acid (TCA) protocol and ressuspend in 6M urea solution. Proteins were then reduced with dithiothreitol (5 mM) alkylated with iodoacetamide (20 mM) and precipitated again via standard methanol/chloroform extraction. Proteins were resuspended in 50 ml 6M urea and urea was adjusted to a final concentration of 1 M. Samples were digested at 37 °C ON with trypsin. Peptides were purified using SEP-C18 purification columns and peptides dry using speed vac. Dried tryptic peptides were reconstituted in 15µl of LC-MS grade water containing 2% acetonitrile and 0.1% trifluoroacetic acid. Samples were subsequently analysed by nano-liquid chromatography tandem mass spectrometry (LC-MS/MS) using a Dionex Ultimate 3000 UPLC coupled to a Q Exactive hybrid quadrupole orbitrap mass spectrometer (ThermoFisher Scientific) as described previously (Chung et al., 2016; Lochmatter et al., 2016; Michalski et al., 2011). Briefly, peptides were desalted on a PepMapC100 column (100µm x 20mm,

5μm particle size, ThermoFisher Scientific) for 3minute at a flow rate of 8 μl/min and separated on a directly coupled nEASY column (PepMap C18, 75 μm x 500mm, 2 μm particle, ThermoFisher Scientific) using a multistep gradient starting with 3 min at 2% Acetonitrile in 5% DMSO with 0.1% Formic acid (buffer B), followed by 60 min linear gradient up to 35% buffer B at 250nl/min flow rate, 7 min linear gradient up to 99% buffer B and maintained at 99% buffer B for 5 min at a flow rate of 250nl/min before reverting to 2% buffer B for 3 min prior to a last 4 min at 99% solvent B. Full MS scans were acquired over the m/z range of 380 - 1800 at a resolution of 70,000 at 200m/z (AGC target of $3x10^6$ ions). MS/MS data was acquired in a data dependent manner by selecting the 15 most abundant precursor ions for HCD fragmentation (CE of 28) and on MS/MS resolution of 17,500.

Quantitative proteomics and data analysis

Label-free quantitation was used to identify overexpressed proteins in SPRTN depleted cells. Raw LC-MS/MS data was uploaded into Progenesis QI Proteomics v2.0 software (Waters). After sample runs alignment, filtering, peak detection and quantification/normalisation (using default parameters), a peak list containing all peptide precursor ions detected across all experimental conditions and biological replicates was generated and exported as mgf file. Peptide and protein identifications was performed using MASCOT v2.5. Data was searched against the Human UniProt SwissProt database (20,268 Homo sapiens sequences; retrieved 20151126) using the Decoy function, whilst selecting trypsin as enzyme (allowing 1 miscleavages), peptide charge of +2, +3, +4 ions, peptide tolerance of 10 ppm and MS/MS of 0.05 Da; $^{\#13}C$ at 1; Carboamidomethylation as fixed modification, and Oxidation (M) and Deamidation (N and Q) as a variable modification. MASCOT data search results were filtered using ion score cut off at 20 and a false discovery rate (FDR) of 1%. Filtered data was imported into Progenesis for subsequent revision of normalization and statistical analysis. Only proteins identified with at least two peptides (peptide counts) and quantified with at least two peptides (unique peptides) were considered for statistical analysis. Differentially expressed proteins and in particular proteins overexpressed in SPRTN depleted cells were selected by p-Anova $p<0.05$ and fold change. The gene ontology tool (PANTHER; Protein ANalysis THrough Evolutionary relationships, version 10.) was used to determine the protein function class of the proteins significantly overexpressed in SPRTN depleted cells.

Statistical analysis

Statistical analysis was done by GraphPad Prism software using unpaired *t*-test or as otherwise described under individual methods subheadings. p value = ns; non-significant, $*p < 0.05$, $**p < 0.01$ and $***p < 0.001$.

In silico methods

Disordered protein regions were predicited using PONDR-FIT (Xue et al., 2010).

DNA binding residues were predicted using MetaDBsite (Si et al., 2011).

SUPPLEMENTARY REFERENCES

Chung, V.Y., Konietzny, R., Charles, P., Kessler, B., Fischer, R., and Turney, B.W. (2016). Proteomic changes in response to crystal formation in Drosophila Malpighian tubules. Fly (Austin) *10*, 91-100.

Harper, J.V. (2005). Synchronization of cell populations in G1/S and G2/M phases of the cell cycle. Methods Mol Biol *296*, 157-166.

Henderson, C.J., and Wolf, C.R. (1992). Immunodetection of proteins by Western blotting. Methods Mol Biol *10*, 221-233.

Jin, Y., Xu, X.L., Yang, M.C., Wei, F., Ayi, T.C., Bowcock, A.M., and Baer, R. (1997). Cell cycle-dependent colocalization of BARD1 and BRCA1 proteins in discrete nuclear domains. Proc Natl Acad Sci U S A *94*, 12075-12080.

Kiianitsa, K., and Maizels, N. (2013). A rapid and sensitive assay for DNA-protein covalent complexes in living cells. Nucleic acids research *41*, e104.

Lessel, D., Vaz, B., Halder, S., Lockhart, P.J., Marinovic-Terzic, I., Lopez-Mosqueda, J., Philipp, M., Sim, J.C., Smith, K.R., Oehler, J*., et al.* (2014). Mutations in SPRTN cause early onset hepatocellular carcinoma, genomic instability and progeroid features. Nature genetics *46*, 1239-1244.

Link, A.J., and LaBaer, J. (2011). Trichloroacetic acid (TCA) precipitation of proteins. Cold Spring Harb Protoc *2011*, 993-994.

Lochmatter, C., Fischer, R., Charles, P.D., Yu, Z., Powrie, F., and Kessler, B.M. (2016). Integrative Phosphoproteomics Links IL-23R Signaling with Metabolic Adaptation in Lymphocytes. Sci Rep *6*, 24491.

Mendez, J., and Stillman, B. (2000). Chromatin association of human origin recognition complex, cdc6, and minichromosome maintenance proteins during the cell cycle: assembly of prereplication complexes in late mitosis. Mol Cell Biol *20*, 8602-8612.

Michalski, A., Damoc, E., Hauschild, J.P., Lange, O., Wieghaus, A., Makarov, A., Nagaraj, N., Cox, J., Mann, M., and Horning, S. (2011). Mass spectrometry-based proteomics using Q Exactive, a high-performance benchtop quadrupole Orbitrap mass spectrometer. Mol Cell Proteomics *10*, M111 011015.

Petermann, E., Woodcock, M., and Helleday, T. (2010). Chk1 promotes replication fork progression by controlling replication initiation. Proc Natl Acad Sci U S A *107*, 16090-16095.

Si, J., Zhang, Z., Lin, B., Schroeder, M., and Huang, B. (2011). MetaDBSite: a meta approach to improve protein DNA-binding sites prediction. BMC Syst Biol *5 Suppl 1*, S7.

Sirbu, B.M., Couch, F.B., and Cortez, D. (2012). Monitoring the spatiotemporal dynamics of proteins at replication forks and in assembled chromatin using isolation of proteins on nascent DNA. Nat Protoc *7*, 594-605.

Tom, R., Bisson, L., and Durocher, Y. (2008). Transfection of Adherent HEK293-EBNA1 Cells in a Six-Well Plate with Branched PEI for Production of Recombinant Proteins. CSH Protoc *2008*, pdb prot4978.

Xue, B., Dunbrack, R.L., Williams, R.W., Dunker, A.K., and Uversky, V.N. (2010). PONDR-FIT: a meta-predictor of intrinsically disordered amino acids. Biochim Biophys Acta *1804*, 996-1010.

Zhitkovich, A., and Costa, M. (1992). A simple, sensitive assay to detect DNA-protein crosslinks in intact cells and in vivo. Carcinogenesis *13*, 1485-1489.
