# Supplementary material for: Metalloprotease SPRTN/DVC1 Orchestrates Replication-Coupled DNA-Protein Crosslink Repair
Source: Mol Cell. 2016 Nov 17;64(4):704–19. doi: 10.1016/j.molcel.2016.09.032 (PMC5128727; doi:10.1016/j.molcel.2016.09.032)
Supplement: Document S2. Article plus Supplemental Information [file mmc2.pdf]

Molecular Cell

Article

Metalloprotease SPRTN/DVC1 Orchestrates Replication-Coupled DNA-Protein Crosslink Repair

Graphical Abstract

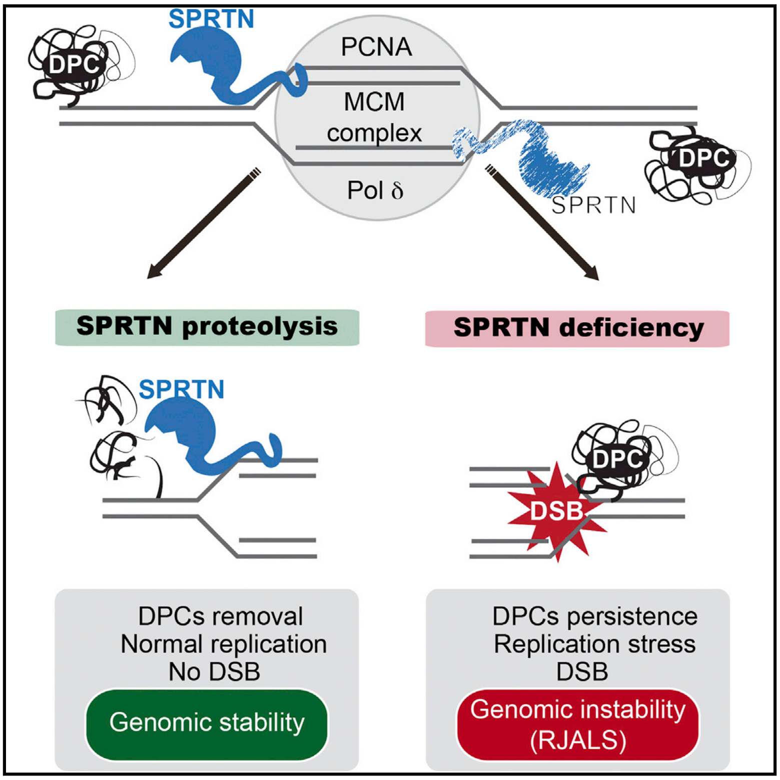

Authors

Bruno Vaz, Marta Popovic,
Joseph A. Newman, ...,
Gillies W. McKenna, Opher Gileadi,
Kristijan Ramadan

Correspondence

kristijan.ramadan@oncology.ox.ac.uk

In Brief

Monogenic mutations in *SPRTN* cause genomic instability, premature aging, and hepatocellular carcinoma. The molecular mechanism of how SPRTN protects genome stability and prevents accelerated aging and cancer is not clear. Vaz, Popovic, et al. show that SPRTN is a DNA replication-coupled metalloprotease for DNA-protein crosslink repair in proliferative human cells.

Highlights

- DNA-protein crosslinks (DPCs) stall DNA replication and induce genomic instability
- SPARTAN (SPRTN) is a DNA replication-coupled metalloprotease which proteolyses DPCs
- SPRTN metalloprotease is a fundamental enzyme in DPC repair pathway
- Ruijs-Aalfs syndrome is caused by a defect in DPC repair due to mutations in *SPRTN*

Accession Numbers

PXD004154

Vaz et al., 2016, Molecular Cell *64*, 704–719
November 17, 2016 © 2016 The Authors. Published by Elsevier Inc.
http://dx.doi.org/10.1016/j.molcel.2016.09.032

Molecular Cell
Article

Metalloprotease SPRTN/DVC1 Orchestrates Replication-Coupled DNA-Protein Crosslink Repair

Bruno Vaz,[1,7] Marta Popovic,[1,7] Joseph A. Newman,[2] John Fielden,[1] Hazel Aitkenhead,[2] Swagata Halder,[1] Abhay Narayan Singh,[1] Iolanda Vendrell,[1,3] Roman Fischer,[3] Ignacio Torrecilla,[1] Neele Drobnitzky,[1] Raimundo Freire,[4] David J. Amor,[5,6] Paul J. Lockhart,[5,6] Benedikt M. Kessler,[3] Gillies W. McKenna,[1] Opher Gileadi,[2] and Kristijan Ramadan[1,8,*]
[1]Cancer Research UK and Medical Research Council Oxford Institute for Radiation Oncology, Department of Oncology, University of Oxford, Oxford OX3 7DQ, UK
[2]Structural Genomics Consortium, University of Oxford, Oxford OX3 7DQ, UK
[3]TDI Mass Spectrometry Laboratory, Target Discovery Institute, Nuffield Department of Medicine, University of Oxford, Oxford OX3 7FZ, UK
[4]Unidad de Investigación, Hospital Universitario de Canarias, Instituto de Tecnologías Biomédicas, Ofra s/n, 38320 La Laguna, Tenerife, Spain
[5]Bruce Lefroy Centre for Genetic Health Research, Murdoch Childrens Research Institute, Royal Children's Hospital, Parkville, VIC 3052, Australia
[6]Department of Paediatrics, The University of Melbourne, Parkville, VIC 3052, Australia
[7]Co-first author
[8]Lead Contact
*Correspondence: kristijan.ramadan@oncology.ox.ac.uk
http://dx.doi.org/10.1016/j.molcel.2016.09.032

SUMMARY

The cytotoxicity of DNA-protein crosslinks (DPCs) is largely ascribed to their ability to block the progression of DNA replication. DPCs frequently occur in cells, either as a consequence of metabolism or exogenous agents, but the mechanism of DPC repair is not completely understood. Here, we characterize SPRTN as a specialized DNA-dependent and DNA replication-coupled metalloprotease for DPC repair. SPRTN cleaves various DNA binding substrates during S-phase progression and thus protects proliferative cells from DPC toxicity. Ruijs-Aalfs syndrome (RJALS) patient cells with monogenic and biallelic mutations in *SPRTN* are hypersensitive to DPC-inducing agents due to a defect in DNA replication fork progression and the inability to eliminate DPCs. We propose that SPRTN protease represents a specialized DNA replication-coupled DPC repair pathway essential for DNA replication progression and genome stability. Defective SPRTN-dependent clearance of DPCs is the molecular mechanism underlying RJALS, and DPCs are contributing to accelerated aging and cancer.

INTRODUCTION

Numerous endogenous and exogenous factors constantly attack the genome causing a variety of chemically distinct DNA lesions (Lindahl, 1993). If not repaired, such DNA lesions lead to genomic instability and cell death (Jackson and Bartek, 2009). Cells have evolved multiple DNA repair pathways, specialized for distinct types of DNA lesions (Friedberg et al., 2006; Lindahl and Wood, 1999). DNA-protein crosslinks (DPCs) represent a so far under investigated type of DNA lesion caused by the covalent attachment of proteins to nucleobases, sugar, or broken phosphodiester bonds on the DNA backbone (Ashour et al., 2015; Tretyakova et al., 2015). Very little is known about how cells remove DPCs and repair DPC-induced DNA lesions (Barker et al., 2005; Connelly and Leach, 2004; Stingele and Jentsch, 2015; Tretyakova et al., 2015). DPCs are induced by chemical reactions catalyzed by products of cellular metabolism like aldehydes or by exogenous sources including UV-light and ionizing radiation (Ide et al., 2011; Shi et al., 2004). Virtually any protein in close vicinity to DNA could form a non-enzymatic DPC in the presence of a crosslinking compound such as formaldehyde (FA) (Shoulkamy et al., 2012). DPCs are also induced enzymatically when certain DNA-binding enzymes form transient covalent interactions with DNA during their physiological reaction cycles. The best-studied enzymatic DPCs are topoisomerases 1 and 2α (Topo1 and Topo2α), known as Topo1- or Topo2α-cleavage complexes (Topo-ccs) (Ashour et al., 2015; Maede et al., 2014). Topo1-ccs or Topo2-ccs are removed by tyrosyl-DNA phosphodiesterase-1 or -2 (TDP1 or TDP2), after proteolysis of Topos into small peptide fragments (max 15–108 amino acids) by an unknown mechanism (Debéthune et al., 2002; Interthal and Champoux, 2011). This suggests the existence of a protease that processes Topos upstream of TDP1 or 2 (Zhang et al., 2006). Despite the frequent occurrence of endogenous non-enzymatic and enzymatic DPCs in cells, the mechanism of DPC removal is still largely unknown (Ide et al., 2015).

Studies in bacteria, yeast, and higher eukaryotes suggest that several canonical DNA repair pathways, including nucleotide excision repair, homologous recombination, and Fanconi anemia repair pathway together with proteasome-dependent protein degradation are involved in the removal of DPCs (Barker

 © 2016 The Authors. Published by Elsevier Inc.
This is an open access article under the CC BY license (http://creativecommons.org/licenses/by/4.0/).

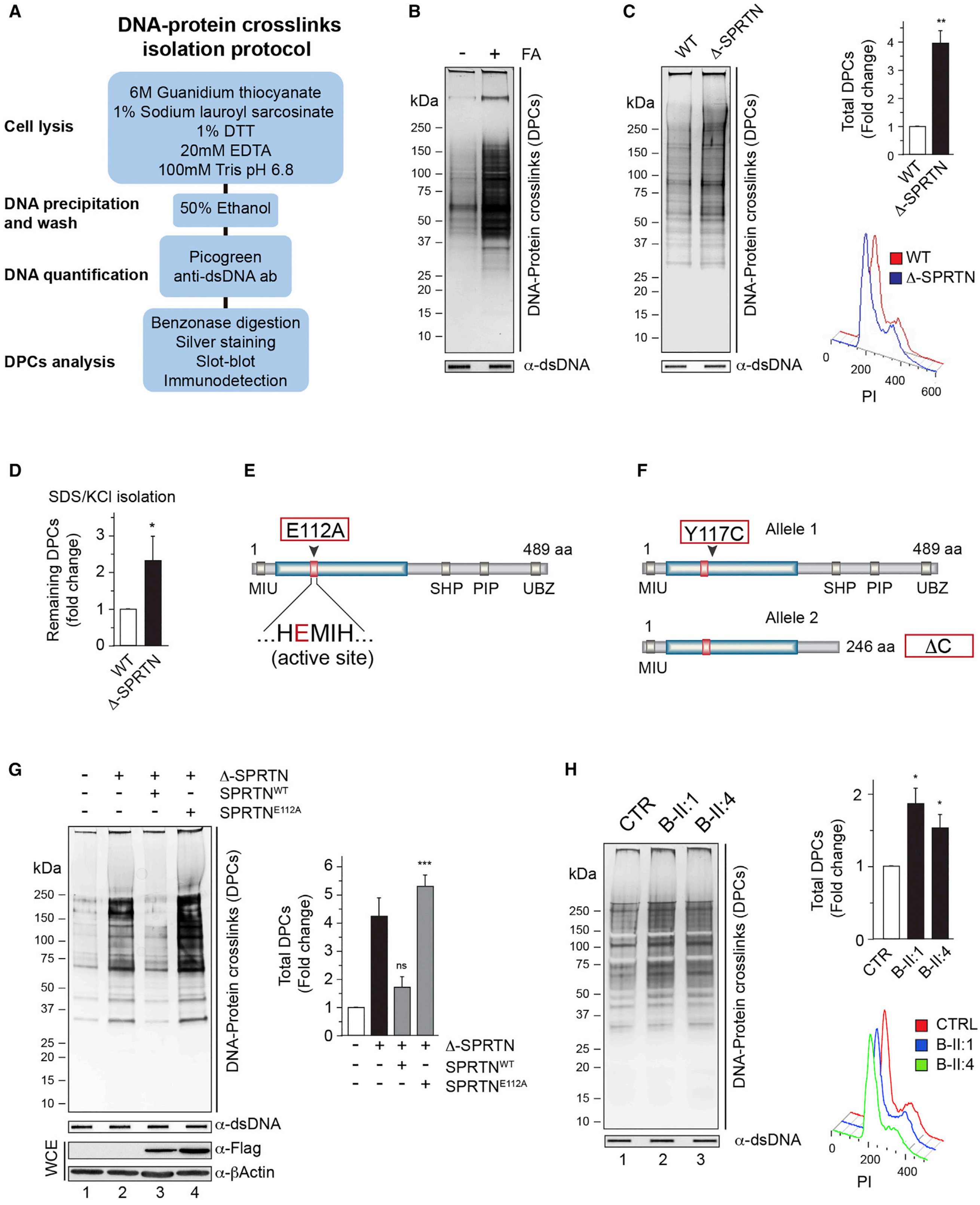

(legend on next page)

et al., 2005; de Graaf et al., 2009; Nakano et al., 2007; Salem et al., 2009). Recently, a yeast DNA-dependent protease Wss1 (weak suppressor of smt3) was shown to protect yeast cells from FA-induced toxicity and, in coordination with Tdp1, process Topo1-ccs (Stingele et al., 2014).

We are currently not aware of any specialized DPC repair pathway in metazoans, although DPC removal is essential for DNA replication fork progression (Kuo et al., 2007; Reardon et al., 2006). Recent biochemical data in *Xenopus* egg extract demonstrated that DPC removal is coupled to DNA replication in a proteasome-independent, but protease-dependent manner (Duxin et al., 2014). However, the protease required for the removal of DPCs during DNA replication remained unknown (Duxin and Walter, 2015; Reardon et al., 2006).

Ruijs-Aalfs syndrome (RJALS), also known as SPARTAN syndrome, is a human autosomal recessive disease characterized by chromosomal instability, premature aging, and early onset hepatocellular carcinoma in children. RJALS is caused by monogenic and biallelic mutations in *SPRTN* (*DVC1*), and a single missense mutation in a putative metalloprotease SprT domain ($SPRTN^{Y117C}$) is pathogenic and responsible for premature aging and liver cancer in humans (Lessel et al., 2014; Ramadan et al., 2016). While the roles of the C-terminal domains of SPRTN have been extensively characterized in translesion DNA synthesis and recruitment to DNA damage foci, the function of the SprT domain, localized in the N-terminal part, is completely unknown (Centore et al., 2012; Davis et al., 2012; Ghosal et al., 2012; Mosbech et al., 2012). At the cellular level, RJALS cells exhibit DNA replication stress, specifically slower replication, and increased numbers of stalled forks and DNA double-strand breaks (Lessel et al., 2014).

Given the importance of SPRTN in genome stability, and the fact that bioinformatic analysis suggests that SPRTN and the yeast protease Wss1 are both distantly related to the Zinicin family of metalloproteases (Stingele et al., 2015), we asked whether SPRTN is a metalloprotease responsible for DPC repair.

Here, we show that SPRTN is a DNA-dependent protease that protects human proliferative cells from DPC toxicity. SPRTN associates with the DNA replication machinery and removes DPCs during DNA synthesis, and thus RJALS is caused by a defect in DPC repair. Altogether, we identified the mechanism for DPC removal from chromatin in human cells and highlighted the importance of this mechanism for genome stability and its relevance for human pathogenesis in accelerated aging and carcinogenesis.

RESULTS

SPRTN Prevents Accumulation of Endogenous DNA-Protein Crosslinks

To investigate the role of SPRTN in DPC repair, we isolated total genomic DNA from HeLa cells and analyzed the amount of DPCs using rapid approach to DNA adducts recovery (RADAR) coupled to SDS-PAGE/silver staining (Figure 1A) (Kiianitsa and Maizels, 2013). The isolation of DNA under stringent denaturing conditions allows us to exclusively detect proteins that are crosslinked to DNA (DPCs). DPC isolates were quantified for total DNA amount to ensure equal amount of DNA for DPC analysis and then treated with Benzonase, to remove all DNA and RNA, prior to SDS-PAGE/silver staining detection. Proteinase K treatment of Benzonase-treated DPCs confirms specificity of protein staining by the silver-staining method (Figure S1A). In addition, treating the cells with FA, camptothecin (CPT), or etoposide (ETO), known DPC-inducing agents, as expected causes a huge accumulation of general (Figure 1B) or specific DPCs (Figure S1B).

Quantitative analysis of total DPCs by silver staining in different cell lines (HeLa, HEK293, and T24) revealed that SPRTN depletion resulted in a 2- to 5-fold increase in the total amount of proteins covalently attached to DNA (Figures S1C and S1D and data not shown). Similarly, CRISPR/Cas9-created SPRTN partial knockout HeLa cells (Δ-SPRTN, Figures S1E–S1G) showed a 3- to 4-fold increase in total DPCs (Figure 1C). Accumulation of DPCs observed in Δ-SPRTN cells was further confirmed by SDS/KCl precipitation assay, an indirect method for DPC isolation (Figure 1D). The increase in DPCs in SPRTN-deficient cells was not due to differences in cell-cycle stage (Figures 1C, S1C, and S1D). To investigate if a putative protease domain in SPRTN plays a role in DPC removal, we ectopically expressed SPRTN wild-type ($SPRTN^{WT}$) or E112A ($SPRTN^{E112A}$), a variant containing a glutamic acid to alanine change in a predicted protease active center (HEXXH, H; histidine, E; glutamic acid, X; and any amino acid; Figure 1E), in Δ-SPRTN HeLa cells. Overexpression of $SPRTN^{WT}$, but not $SPRTN^{E112A}$, completely rescued basal DPC accumulation (Figure 1G, compare lanes 3 and 4). To investigate whether RJALS lymphoblastoid cell lines (LCLs) with monogenic and biallelic mutations in *SPRTN* (Figure 1F; SPRTN-ΔC/$SPRTN^{Y117C}$) are also deficient in removal of DPCs, we isolated and analyzed total DPCs in these cells (Figure 1H). RJALS cells showed a 1.5- to 2-fold increase in total DPCs when compared to control LCLs (Figure 1H, compare lane 1 to lanes 2 and 3).

Figure 1. SPRTN Prevents Basal DNA-Protein Crosslinks Accumulation

(A) Schematic of DPC isolation protocol (RADAR).

(B) DPC accumulation in HeLa cells after FA treatment (2.5 mM, 30min).

(C) SPRTN deficiency leads to DPCs accumulation (silver staining). The quantification and cell-cycle profiles are shown (right images).

(D) Quantification of DPC isolates by SDS/KCl precipitation method.

(E) Schematic of SPRTN protein domains and a putative protease active site.

(F) Schematics of biallelic SPRTN mutations in RJALS patients.

(G) Total DPC levels after ectopic expression of SPRTN WT or E112A in Δ-SPRTN HeLa cells and corresponding quantification. whole cell extract: WCE.

(H) Total DPC levels in RJALS patient LCLs from family B (two patients; B-II:1 and B-II:4) and control LCLs (CTR). The quantification and cell-cycle profiles are shown (right image). Mean ± SEM, n = 3.

See also Figure S1.

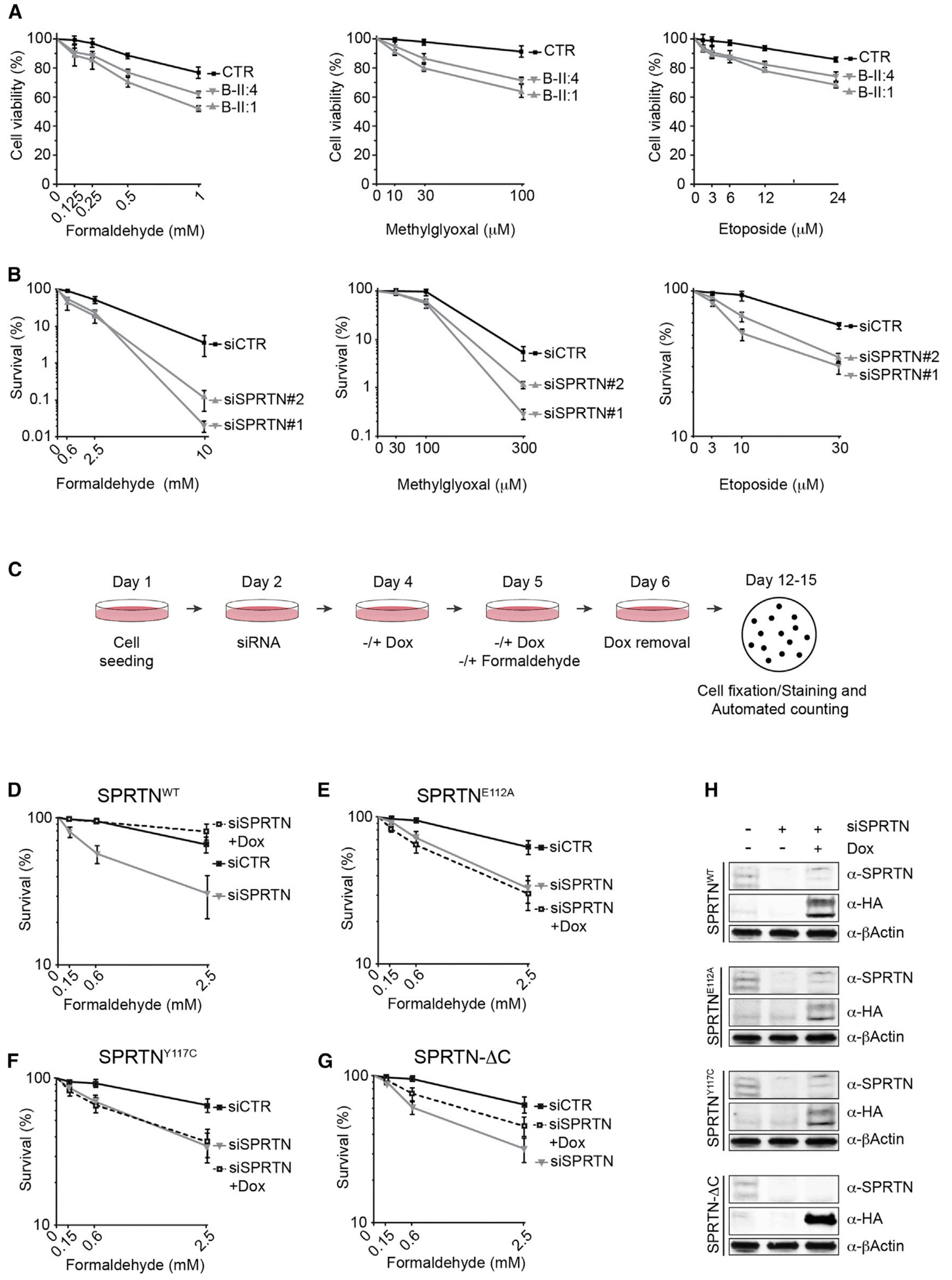

Figure 2. RJALS and SPRTN-Depleted Cells Are Hypersensitive to DPC-Inducing Agents

(A) Cell viability assays of RJALS (B-II:1 and B-II:4) and control (CTR) LCLs after treatment with indicated chemicals.

(B) Clonogenic survival assay of siRNA control (CTR) or siRNA SPRTN-depleted HeLa cells after treatment with indicated chemicals.

(legend continued on next page)

The nucleotide excision repair (NER) and homologous recombination (HR) pathways have also been implicated in DPC repair. However, depletion of the main components of NER (XPC) and HR (MRE11) by small interfering (si)RNA did not lead to an increase to the total amount of DPCs (Figure S2A). Similarly, impairment of the DNA interstrand crosslink repair pathway, Fanconi anemia, by depletion of FANCD2 did not cause DPC accumulation compared to control cells (Figure S2A). Altogether, these results indicate that SPRTN is the main player involved in DPC removal, and, more specifically, that its putative metalloprotease active residue (E112), is essential for removal of endogenously occurring DPCs in human proliferative cells.

RJALS and SPRTN-Depleted Cells Are Hypersensitive to DPC-Inducing Agents

We further asked whether RJALS patient LCLs and siRNA SPRTN-depleted HeLa cells are hypersensitive to DPC-inducing agents. We used FA and methylglyoxal, which induce general DPCs (Figure 1B and data not shown), and CPT and ETO, which induce specific enzymatic DPCs (Figure S1B). Both RJALS patient cells (Figure 2A) and SPRTN-depleted cells (Figures 2B and S2B) were hypersensitive to DPC-inducing agents. To analyze how individual SPRTN mutations found in RJALS patients affect cell sensitivity to DPC-inducing agents, we created doxycycline (DOX) inducible and stable Flp-In HeLa cell lines, expressing wild-type (WT), enzymatic dead protein (E112A), and patient variants of SPRTN (Figures 2C–2H and S2C–S2F). Ectopic expression of $SPRTN^{WT}$, where endogenous SPRTN was depleted with siRNA targeting the 3′ UTR of SPRTN transcripts, rescued SPRTN-depleted cells' hypersensitivity to FA (Figure 2D) or CPT (Figure S2C). Conversely, overexpression of $SPRTN^{E112A}$ or patient variant $SPRTN^{Y117C}$ was not able to rescue hypersensitivity to FA (Figures 2E and 2F, respectively) or CPT (Figures S2D and S2E, respectively). Ectopic expression of the truncated patient variant SPRTN-ΔC, which still contains an intact putative metalloprotease domain, partially rescued hypersensitivity to FA (Figure 2G) or CPT (Figure S2F). Both patient mutations are defective in their ability to protect cells from DPCs, although SPRTN-ΔC to a lesser extent, since RJALS LCLs are hypersensitive to DPC-inducing agents (Figure 2A).

Next, to address if the cytotoxicity observed in SPRTN-deficient cells is due to accumulation of DPCs, we co-depleted Topo1 and SPRTN and monitored cell survival following CPT treatment. Co-depletion of Topo1 completely rescued sensitivity to CPT in SPRTN-depleted cells (Figure S2G), confirming that CPT-induced toxicity in SPRTN-depleted cells is due to accumulation of Topo1-ccs. Co-depletion of TDP1, a key player in Topo1-cc removal, does not further hypersensitize SPRTN-depleted cells to CPT, suggesting that both proteins work in the same pathway (Figure S2H).

The Fanconi anemia pathway is known to protect from formaldehyde-induced toxicity. As expected, inactivation of the Fanconi anemia pathway by FANCD2 siRNA depletion hypersensitizes cells to FA-treatment, but not CPT-treatment (Figure S2I). This further suggests that the Fanconi anemia pathway is strictly involved in repair of DNA-interstrand crosslinks (also induced by FA treatment), but not DNA-protein crosslinks (e.g., removal of a specific DPC induced by CPT). Altogether, these results suggest that SPRTN forms a unique DNA repair pathway for DPC removal.

SPRTN Is a DNA-Dependent Metalloprotease

Intensive work from several laboratories was unable to identify SPRTN protease activity (Davis et al., 2012; Kim et al., 2013; Mosbech et al., 2012). Our results led us to re-evaluate published data and investigate whether SPRTN is indeed a protease. To this end, we purified $SPRTN^{WT}$, $SPRTN^{E112A}$, two patient variants ($SPRTN^{Y117C}$ and SPRTN-ΔC), and several C-terminally truncated variants of SPRTN using an *E. coli* protein expression system.

Considering that the SprT domain is classified as a putative metalloprotease domain, we first analyzed the presence of metal in the SPRTN protein. We purified SPRTN lacking the C-terminal Zn-binding UBZ domain and PIP-box (SPRTN 1–268) and analyzed it by mass spectrometry. Intact protein analysis of SPRTN under native and denaturing conditions revealed a mass increase of 126 daltons, which is consistent with the presence of two zinc ions (Figure S3A). The recent finding that the yeast protease Wss1 requires DNA to elicit proteolytic activity (Balakirev et al., 2015; Stingele et al., 2014) led us to investigate whether SPRTN binds DNA. We used 63 bp double-strand DNA probes (dsDNA) labeled with fluorescein isothiocyanate (FITC) to analyze SPRTN binding affinity by fluorescence polarization. $SPRTN^{WT}$ protein showed high affinity to dsDNA (dissociation constant [K_D] ≈100 nM) (Figure 3A). In silico analysis of the SPRTN secondary structure revealed the presence of five DNA binding regions: four motifs in the C-terminal part of the protein and one in the SprT protease domain (Figures 3B and S3B). The removal of predicted C-terminal DNA binding sites strongly decreased the DNA binding affinity of SPRTN (Figure 3A).

Next, we asked whether SPRTN binds different DNA structures. $SPRTN^{WT}$ binds single-strand DNA (ssDNA), dsDNA, and splayed DNA with similar affinities (Figure 3C). $SPRTN^{E112A}$ and $SPRTN^{Y117C}$ exhibited similar affinities to DNA as $SPRTN^{WT}$. The truncated variant of SPRTN-ΔC (1–246) showed reduced affinity to DNA (K_D ≈ 0.48 μM; Figure 3A), which correlates with the loss of putative DNA-binding sites (Figures 3B and S3B).

We observed increased levels of $SPRTN^{WT}$ degradation when the reaction of SPRTN and DNA was incubated for a longer period of time and analyzed by SDS-PAGE/Coomassie blue staining. Full-length $SPRTN^{WT}$ protein was mostly intact and ran at the predicted size of 55 kDa (Figure 3D, lane 1). However, incubation of $SPRTN^{WT}$ with dsDNA induced strong degradation of SPRTN with several visible protein bands and a prominent accumulation of a ∼25 kDa fragment (Figure 3D, lane 2). Mass spectrometry analysis of $SPRTN^{WT}$ degradation products

(C) Schematic of experimental setup for clonogenic survival assays in doxycycline inducible (+Dox) SPRTN Flp-In HeLa cell lines.
(D–G) Ectopic expression of $SPRTN^{WT}$ (D), $SPRTN^{E112A}$ (E), $SPRTN^{Y117C}$ (F), and SPRTN-ΔC (G) after depletion of endogenous SPRTN.
(H) Western blot (WB) demonstrating SPRTN depletion and/or overexpression. Mean ± SEM, n = 3.
See also Figure S2.

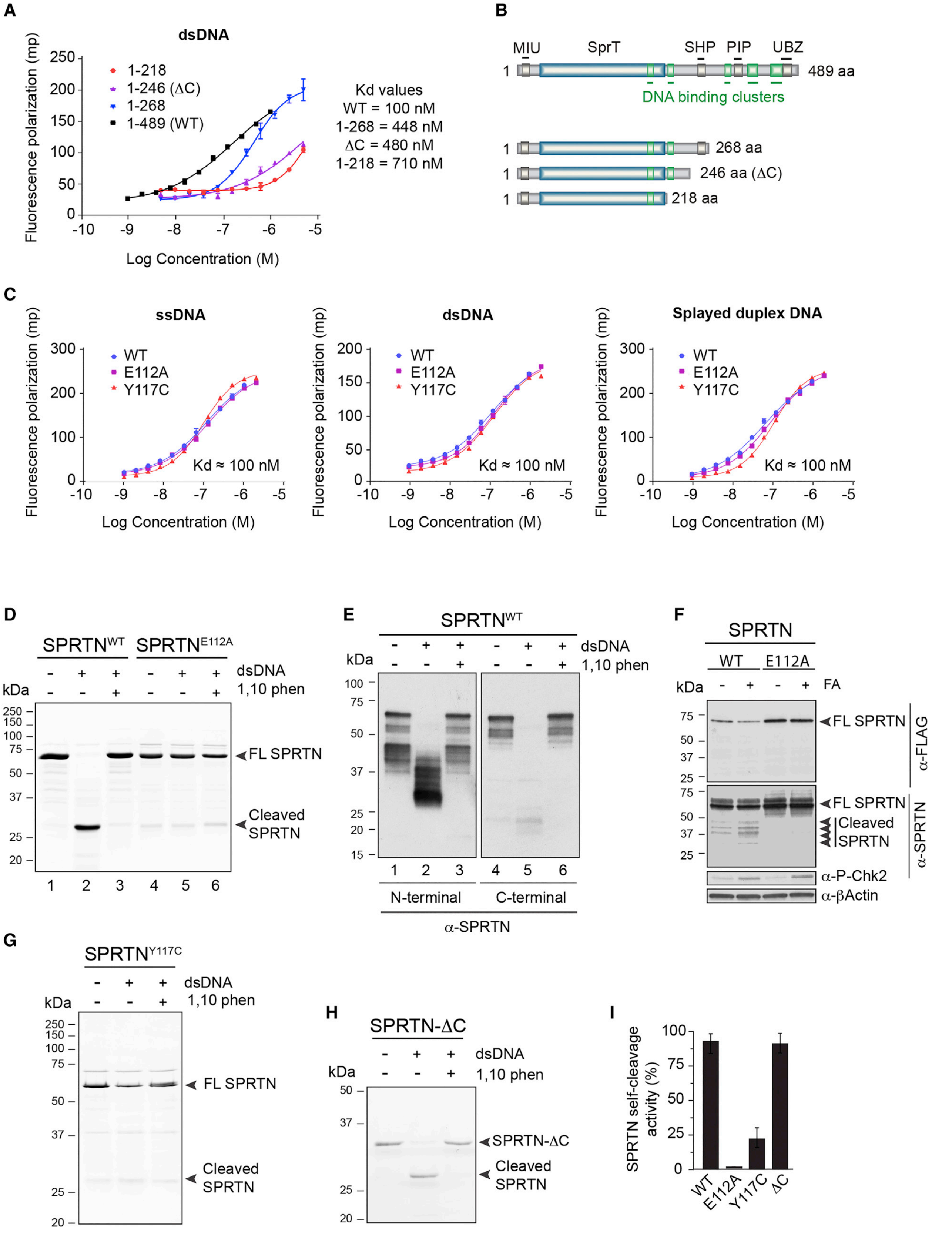

(legend on next page)

identified at least five different cleavage products in the SPRTN protein (Figure S7C), with cleavage sites located in its C-terminal part (Figure S3C). $SPRTN^{WT}$ auto-cleavage activity was inhibited in the presence of 1,10 phenanthroline, a known inhibitor of Zn^{2+}-dependent metalloproteases (Figure 3D, lane 3). SPRTN auto-cleavage activity was abolished by the E112A mutation (Figure 3D, lane 5). Western blot analysis of $SPRTN^{WT}$ auto-cleavage products with antibodies raised against N- or C-terminal fragments of SPRTN further confirmed mass spectrometry data and demonstrated that SPRTN protein was mainly cleaved within its C-terminal part, while the N-terminal fragment remained mostly intact (Figure 3E, lanes 2 and 5). These data suggest that SPRTN is a DNA and Zn-dependent protease, which possesses auto-cleavage activity in vitro. $SPRTN^{WT}$, but not $SPRTN^{E112A}$, auto-cleavage products were also visible in HEK293 cells, especially after FA treatment, demonstrating SPRTN auto-cleavage activity in vivo (Figure 3F).

We further asked what is the minimum length of DNA required to activate SPRTN cleavage activity (Figure S3D). Different sized probes of ss or dsDNA were incubated with $SPRTN^{WT}$ protein. DNA fragments of 100-mer ssDNA or dsDNA were the most efficient activators of SPRTN auto-cleavage activity.

In order to investigate if RJALS patient mutations are defective with respect to their auto-cleavage activity, we tested the two patient variants, $SPRTN^{Y117C}$ and SPRTN-ΔC (Figures 3G–3I). SPRTN-ΔC showed similar auto-cleavage activity to $SPRTN^{WT}$, whereas $SPRTN^{Y117C}$ showed an ~80% reduction in auto-cleavage activity compared to $SPRTN^{WT}$ (Figure 3I). Considering that $SPRTN^{Y117C}$ binds DNA with similar affinity to $SPRTN^{WT}$, we conclude that its enzymatic deficiency is not due to defective DNA binding. Our results suggest that $SPRTN^{Y117C}$ directly affects the protease active center (E112), which is located only five amino acids upstream of the mutation. SPRTN-ΔC (1–246) still retains auto-cleavage activity similar to $SPRTN^{WT}$ (Figures 3H and 3I) despite its lower DNA affinity (Figure 3A).

Trans-Cleavage Activity of SPRTN Metalloprotease

To address whether SPRTN auto-cleavage occurs in *cis* or in *trans*, we incubated $SPRTN^{WT}$ with the enzymatic-dead variant of SPRTN ($SPRTN^{E112A}$). $SPRTN^{WT}$ cleaved $SPRTN^{E112A}$ (Figure S3E, compare lanes 2 and 4). These data suggest that SPRTN cleaves itself in *trans*. To test the importance of DNA-binding for trans-cleavage activity, we incubated two C-terminal truncated variants of SPRTN that have medium ($SPRTN^{1–268}$) or low ($SPRTN^{1–218}$) DNA affinity with $SPRTN^{E112A}$ (Figure 3A for DNA affinity). $SPRTN^{1–268}$ cleaved $SPRTN^{E112A}$ with similar efficiency to $SPRTN^{WT}$ (Figure S3E, compare lane 4 to lane 6). In contrast, $SPRTN^{1–218}$ markedly lost trans-cleavage activity (Figure S3E, compare lane 4 to lane 8). Next, we asked if DNA is a scaffold or an allosteric activator for SPRTN auto-cleavage activity. $SPRTN^{WT}$ auto-cleavage activity was maximal at equimolar concentrations of protein and DNA in the reaction. Increasing the concentration of DNA gradually inhibited SPRTN activity (Figure S3F). Altogether, these results show that DNA serves as a scaffold, which brings SPRTN and its substrate into proximity, rather than acting as an allosteric activator. Loss of its C-terminal DNA binding regions renders SPRTN unable to perform its proteolytic activity, suggesting that auto-cleavage reduces the proteolytic activity of the cleaved forms.

Identification of SPRTN Substrates

We isolated DPCs from control and SPRTN-depleted HeLa cells (Figure S4A) and analyzed them by label-free quantitative mass spectrometry. Three independent experiments revealed 84 significantly increased (1.5-fold) proteins within the DPCs in SPRTN-depleted cells, in comparison to control cells (Figure S4B and Raw data repository: PRIDE). Although many of the identified substrates were DNA- and RNA-binding proteins, the majority were non-DNA binding proteins (Figure S4C). Given that any protein in close vicinity to DNA can be crosslinked, especially nuclear matrix proteins, this is not a surprising finding. Accordingly, non-DNA binding proteins such as Lamin B1 and DNA-PK are enriched in DPCs isolated from Δ-SPRTN cells (Figure 5G).

We focused on histones and DNA topoisomerases, DNA-binding proteins with well-characterized cellular functions, which emerged among the top hits (≥1.98-fold increase) in our mass spectrometry analysis (Figure S4D). To validate mass spectrometry data, we analyzed specific DPCs, isolated as shown in Figure 1A, by slot-blot immunodetection. Depletion of SPRTN with two different siRNAs significantly increased the amount of Topo1, Topo2α, histone H3, and histone H4 (Figures 4A and 4B). To demonstrate that hyperaccumulation of Topo1 was specific to loss of SPRTN protease activity, we ectopically expressed $SPRTN^{WT}$ or $SPRTN^{E112A}$ in 3′ UTR-targeted siRNA SPRTN-depleted cells. Ectopic expression of $SPRTN^{WT}$, but not $SPRTN^{E112A}$, rescued Topo1 accumulation (Topo1-cc) (Figure S4E). We further analyzed Topo1-ccs following treatment with CPT (Figure 4C). Both SPRTN-depleted cells and patient LCLs accumulate more Topo1-ccs compared with control cells, thus further confirming the fundamental importance of SPRTN protease activity in the removal of Topo1-ccs and protection from DPC cytotoxicity (Figures S2B–S2H).

Figure 3. SPRTN Is a DNA-Dependent Metalloprotease with Auto-Proteolytic Properties

(A) Fluorescence polarization DNA binding assay of SPRTN and its C-terminally truncated variants.

(B) Schematic of in silico predicted DNA binding regions (green squares) in SPRTN protein and in SPRTN C-terminally truncated variants.

(C) DNA binding affinity of $SPRTN^{WT}$, $SPRTN^{E112A}$, and $SPRTN^{Y117C}$ toward ds, ss, and splayed DNA.

(D) DNA induces auto-proteolysis of SPRTN. E112A mutation or 1,10 phenanthroline (phen) inhibits SPRTN activity. The cleavage was analyzed on SDS-PAGE gels and visualized by Coomassie blue staining.

(E) WB illustrating SPRTN auto-cleavage.

(F) WB of total cell lysates after ectopic expression of FLAG-SPRTN (WT or E112A) after FA treatment. The anti-phospho (P) Chk2 represents a positive control for activation of DNA damage signaling.

(G and H) In vitro enzymatic reactions as in (D), with patient mutations as indicated.

(I) Quantification of auto-cleavage activity. Mean ± SD, n = 3.

See also Figure S3.

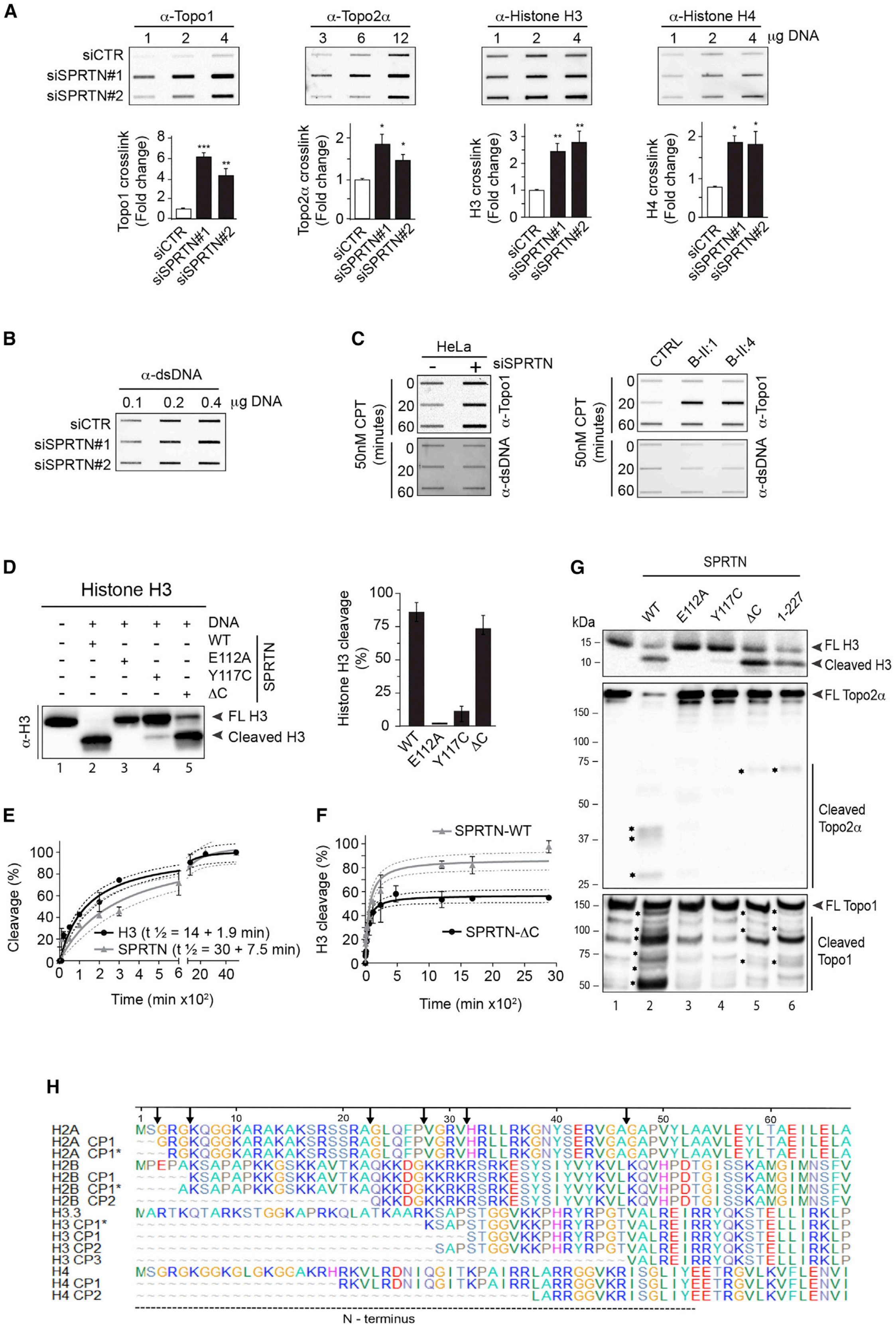

(legend on next page)

Characterization of SPRTN Enzymatic Activity

To characterize the ability of SPRTN to cleave the identified substrates, we performed in vitro cleavage activity assays using purified $SPRTN^{WT}$ protein. $SPRTN^{WT}$ cleaved all tested histones, H2A, H2B, H3, and H4, in a DNA-dependant manner, but not the cytosolic protein glutathione S-transferase (Figures 4D, S5A, and S5B). Kinetic analysis of SPRTN auto-cleavage and histone H3 cleavage in the same reaction revealed that SPRTN cleaves H3 with slightly faster kinetics than itself (Figure 4E). This suggests that SPRTN simultaneously cleaves substrates and itself, but with a higher preference for the substrate. This most probably leads to SPRTN inactivation as the cleaved SPRTN products lose DNA binding affinity (Figure 3A), enzymatic activity (Figure S3E), and have 2-fold lower substrate processivity kinetics (Figure 4F). This might be one of the mechanisms, beside cell-cycle regulation (see below), by which SPRTN protease self regulates to prevent deleterious and uncontrolled cleavage in its vicinity.

We next tested the cleavage activity of various SPRTN variants, focusing on histone H3 (Figures 4D and 4G). As expected, catalytically inactive $SPRTN^{E112A}$ did not cleave histone H3. Similar to SPRTN auto-cleavage activity, patient variant $SPRTN^{Y117C}$ was severely affected with respect to histone H3 cleavage (~8% active), whereas SPRTN-ΔC retained similar activity to $SPRTN^{WT}$. We extended the analysis of the cleavage activity to two other identified substrates, Topo1 and Topo2α. To this end, we purified YFP-Topo1 and GFP-Topo2α from whole cell extracts under denaturing conditions and incubated them with different variants of SPRTN. $SPRTN^{WT}$, but not $SPRTN^{E112A}$, cleaved Topo1 and Topo2α in vitro, thus confirming Topo1 and Topo2α as SPRTN substrates (Figure 4G, lanes 2 and 3). Characterization of patient mutations revealed that $SPRTN^{Y117C}$ was unable to cleave Topo1 and Topo2α. Unexpectedly, in contrast to auto-cleavage (Figure 3H) and cleavage of histone H3 (Figures 4D and 4G), SPRTN-ΔC showed severely reduced proteolysis of Topo1 and Topo2α in vitro, similar to the smallest auto-cleaved form of SPRTN (1–227) (Figure 4G, lanes 5 and 6). These data show that SPRTN protease cleaves various DNA-binding substrates in a DNA-dependent manner. SPRTN protease activity is severely hampered by patient mutation Y117C for all tested substrates. Patient truncated variant SPRTN-ΔC, although able to cleave itself and histone H3, exhibits strongly reduced activity toward Topo1 and Topo2α, suggesting that the C-terminal part of SPRTN is required for optimal proteolysis of these substrates.

SPRTN Is a Pleiotropic Protease for DNA-Binding Proteins

Next, we aimed to identify SPRTN protease cleavage sites and asked if SPRTN protease cleaves at a specific amino acid sequence motif. Cleavage products of H2A, H2B, H3, and H4 as well as auto-cleavage products of SPRTN were analyzed by mass spectrometry to identify cleavage sites (Figures 4H and S5C). We found that SPRTN cleaves histones H2A, H2B, H3, and H4 within their unstructured, positively charged N-terminal tail. All identified cleavage regions were enriched in arginine and lysine residues and were in very close proximity to serine residues in most cases. Analysis of SPRTN auto-cleavage sites (CS) also showed an abundance of lysine, arginine, and serine residues (CS 1 and 3), while specifically CS3 is heavily enriched in serines (and, to a lesser extent, lysines and arginines) (Figure S3C). Similar to cleavage sites of histones, which are present in disordered protein regions (N terminus), SPRTN cleaves itself in multiple places in its C terminus, which is predominantly disordered (Figure S5D). These results suggest that SPRTN is not a sequence-specific protease, but cleaves unstructured protein regions in the vicinity of lysine, arginine, and serine residues.

To test whether SPRTN also cleaves DPCs in vitro, we isolated total DPCs by SDS/KCl precipitation assay and incubated them with recombinant SPRTN. $SPRTN^{WT}$, but not $SPRTN^{E112A}$, cleaves DPCs in vitro (Figure S6A). Thus far, our in vitro and in vivo results suggest that SPRTN cleaves DNA-binding proteins regardless of their DNA-binding status (i.e., covalent DPCs or non-covalent). To further test this observation, we isolated chromatin-bound Topo1 or Topo2 under denaturing conditions from both untreated HEK293 cells and those treated with CPT or ETO, respectively. SPRTN cleavage efficiency of immunopurified Topo1 and Topo2α was similar between untreated and Topo1/2α-cc enriched samples (CPT- and ETO-treated, respectively; Figures S6B and S6C). Altogether, these data suggest that SPRTN does not specifically cleave DPCs, but DNA-binding substrates, and that SPRTN is indeed a pleiotropic protease as it cleaves the majority of high-molecular weight DPCs in vitro (Figure S6A, compare lanes 1 and 2).

SPRTN Removes DPCs during S-Phase and Protects Proliferative Cells from DPC-Inducing Agents

Considering that SPRTN expression is absent in the G1-phase of the cell cycle, and is rapidly upregulated as cells enter the S-phase (Mosbech et al., 2012), we hypothesized that SPRTN is involved in the removal of DPCs during DNA synthesis. To

Figure 4. Identification of SPRTN Substrates

(A) Slot-blots showing presence of Topo1, Topo2α, H3, and H4 DPCs after SPRTN depletion in HeLa cells and corresponding quantifications. Mean ± SEM, n = 3.

(B) DNA loading controls for DPC analysis, prior to benzonase treatment as in (A).

(C) Slot-blots showing accumulation of Topo1-ccs after continuous CPT treatment, as indicated, in Δ-SPRTN cells (left image) and RJALS LCLs (right image).

(D) WB and corresponding quantification showing that SPRTN cleaves histone H3 in the presence of DNA. Mean ± SD, n = 3.

(E) Time kinetics of H3 cleavage versus SPRTN self-cleavage within the same reaction mixtures expressed as increase in cleavage of either H3 or SPRTN over time (min). Mean ± SD, n = 3.

(F) Comparison of time, response of H3 cleavage for $SPRTN^{WT}$, and SPRTN-ΔC as in (D). y axis was normalized to a 0%–100% scale. Mean ± SD, n = 3.

(G) Histone H3, Topo1 and Topo2α are substrates of SPRTN protease. The cleavage products (*) were detected by WB using antibodies against histone H3 (upper image), Topo2α (middle image), or Topo1 (lower image). FL, full length.

(H) Multiple sequence alignment of histones' cleavage products (CP) showing cleavage sites (arrow) in their unstructured N-terminal tails (* denotes alternative cleavage products).

See also Figures S4 and S5.

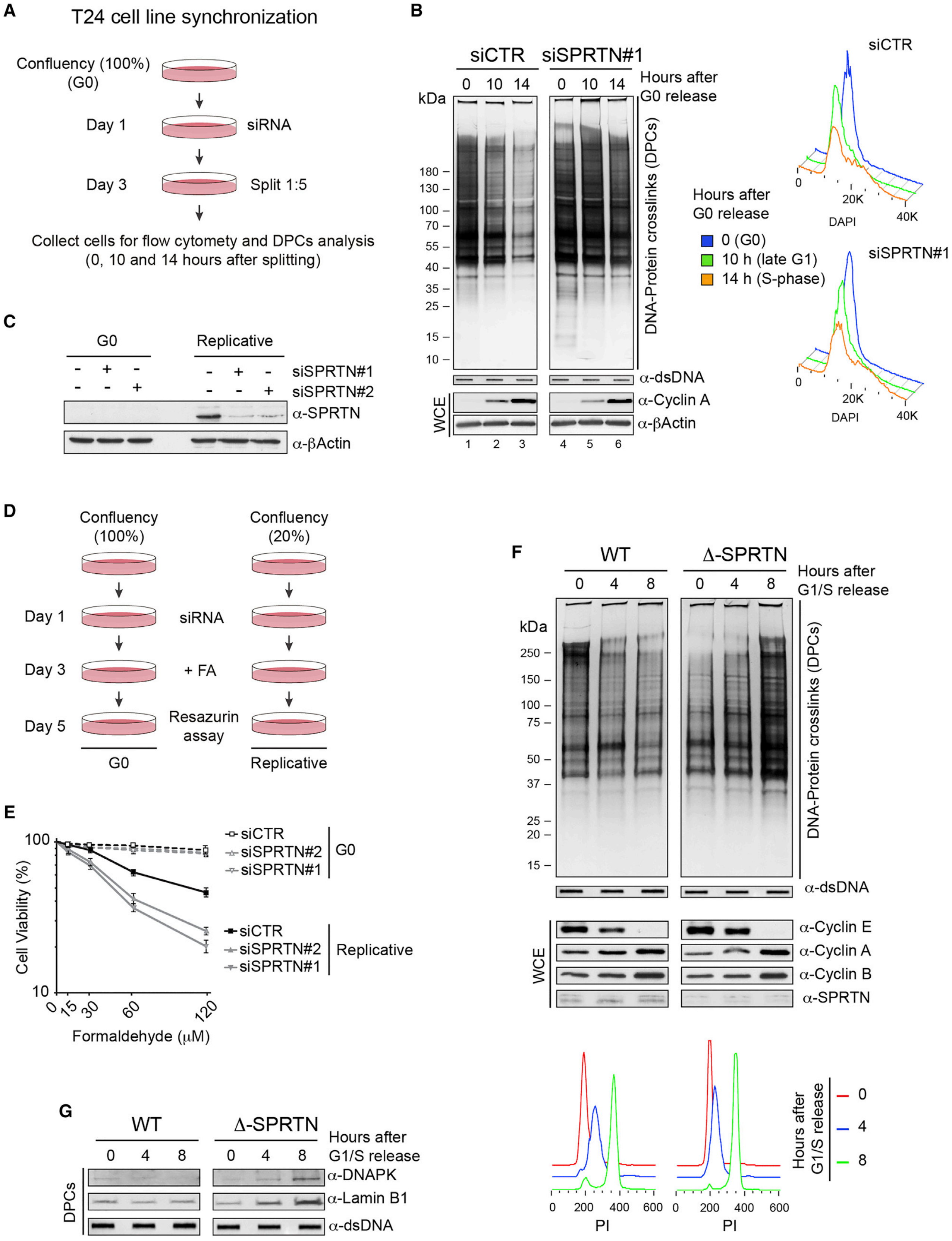

(legend on next page)

test this hypothesis, we analyzed the levels of DPCs during S-phase progression in control and SPRTN-depleted cells. We used T24 cells, which arrest in the G0 phase by contact inhibition at 100% confluency and synchronously enter S-phase when diluted to lower densities. Cells were arrested in G0, treated with control-siRNA or SPRTN-siRNA for 2 days, and then diluted to enter S-phase (Figure 5A). We monitored the total amount of endogenous DPCs during S-phase progression. DPCs were rapidly removed as control cells progressed through S-phase. Conversely, SPRTN-depleted cells showed delayed kinetics of DPC removal during S-phase progression (Figure 5B, compare lanes 2 and 3 with 5 and 6). Western blot analysis of T24 cells revealed that SPRTN is not expressed in G0 cells (Figure 5C), further suggesting an essential role of SPRTN in protection from DPCs during DNA synthesis. To investigate this hypothesis, we treated G0-arrested or proliferative T24 cells with control or SPRTN siRNA for 2 days, exposed the cells to a sub-lethal dose of FA for an additional 2 days, and then monitored cell viability (Figure 5D). Indeed, only SPRTN-depleted proliferative, but not G0-arrested cells, were hypersensitive to FA (Figure 5E). Furthermore, Δ-SPRTN HeLa cells hugely accumulate total DPCs during S-phase progression compared to control cells (Figure 5F). These data show that SPRTN processes DPCs during S-phase progression, thus protecting cells from DPC cytotoxicity.

SPRTN Is a Constitutive Part of the DNA Replication Machinery

Having demonstrated that SPRTN protects proliferative human cells from DPCs and knowing that patient mutation $SPRTN^{Y117C}$ is essential for unperturbed DNA replication fork progression (Lessel et al., 2014), we asked whether SPRTN is a part of the DNA replication machinery. We isolated SPRTN-SSH from total HEK293 cell extracts over Streptactin sepharose under high salt and detergent conditions to remove all unspecific binding proteins from SPRTN-complexes in vivo. SPRTN co-precipitated with the main components of the DNA replication machinery: PCNA, minichromosome maintenance complex (MCM) subunits 2 and 6 and DNA polymerase δ (Figure 6A). We next asked if SPRTN is physically present at sites of DNA replication forks. To address this question, we isolated proteins from nascent DNA by iPOND technology. Similar to PCNA, MCM3, and DNA polymerase δ, SPRTN was present on nascent DNA (Figure 6B, lane 2) and moved with the replisome as shown by thymidine chase (Figure 6B, lane 3). Altogether, these data suggest that SPRTN is part of the DNA replication machinery and moves with the replisome during DNA synthesis.

SPRTN Orchestrates DNA Replication Fork Progression by Removal of DPCs

We further asked whether SPRTN inactivation affects the progression of the DNA replication machinery. We isolated DNA replication forks by iPOND and monitored their progression in control and Δ-SPRTN HeLa cells (Figure 6C). Knock out of SPRTN severely affected progression of DNA replication, as shown by retention of PCNA and MCM3 on mature DNA (thymidine chase, Figure 6C, compare lanes 3 and 4 with lanes 6 and 7). Defects in DNA replication fork progression in Δ-SPRTN cells were demonstrated by DNA fiber assay (Figure S6D). Stalled DNA replication forks in Δ-SPRTN cells accumulated Topo1 (Figure S6E), one of the substrates of SPRTN protease in vitro and in vivo. These results indicate that SPRTN regulates replisome progression and prevents accumulation of Topo1 at sites of DNA replication forks. We further asked whether SPRTN physically interacts with Topos. To this end, we co-precipitated SPRTN, as described earlier, and analyzed the presence of Topo1 and Topo2α in the SPRTN complex. Co-immunoprecipitation experiments revealed that SPRTN indeed forms a complex with both Topo1 and Topo2α in vivo (Figure S6F).

To investigate how DPCs affect the progression of DNA replication, we employed the DNA fiber assay (Figure 6D). Treatment of control cells with a low dose of FA or CPT caused a strong reduction of DNA synthesis, detected as a decrease in DNA replication fork velocity when the second nucleotide (IdU) was incubated along with FA or CPT. Ectopic expression of $SPRTN^{WT}$ rescued the DPC-induced DNA replication fork progression defect in both control and Δ-SPRTN cells (Figures 6D and S6D). By measuring DNA replication fork velocity in RJALS LCLs, we found that RJALS cells showed a stronger reduction (~2.5-fold) in DNA replication fork progression than control cells when challenged with FA or CPT (Figure 6E). Altogether, these results suggest that SPRTN is essential for the progression of DNA replication forks challenged with DPC-inducing agents and is a limiting factor in this process since reduced fork speed in FA/CPT-treated control or Δ-SPRTN cells can be rescued by $SPRTN^{WT}$ overexpression.

SPRTN Prevents DPC-Induced DSBs in the S-Phase

We hypothesized that the decreased velocity of DNA replication forks in SPRTN-depleted or RJALS cells in the presence of DPC-inducing agents leads to prolonged stalling of replisomes and consequently to DNA replication fork collapse, visualized as DNA ds breaks (DSBs), a phenotype observed in RJALS patients (Lessel et al., 2014). We continuously exposed control or Δ-SPRTN cells to mild doses of FA or CPT and monitored the

Figure 5. DPC Removal during S-Phase of the Cell Cycle

(A) Schematic of the experimental approach used to synchronize T24 cell in G0 and monitor DPC levels during S-phase entry.

(B) Total DPC levels in G0-, late G1-, and S-phase before and after SPRTN depletion in HeLa cells visualized by silver staining. The WB of cyclin A and cell-cycle profiles were used as a control of S-phase entry.

(C) WB showing SPRTN expression or depletion in non-replicative and replicative T24 cells.

(D) Schematic of cell viability measurement in replicative and non-replicative cells.

(E) Cell viability of replicative and non-replicative T24 cells after SPRTN depletion and FA treatment.

(F) Total DPC levels after G1/S release in HeLa WT and Δ-SPRTN cells visualized by silver staining. The WB of cyclins and cell-cycle profiles (lower images) were used to control S-phase progression.

(G) WB showing presence of DNA-PK and Lamin B1 in DPCs in Δ-SPRTN HeLa cells after G1/S release. Whole cell extract: WCE. Mean ± SEM, n = 3.

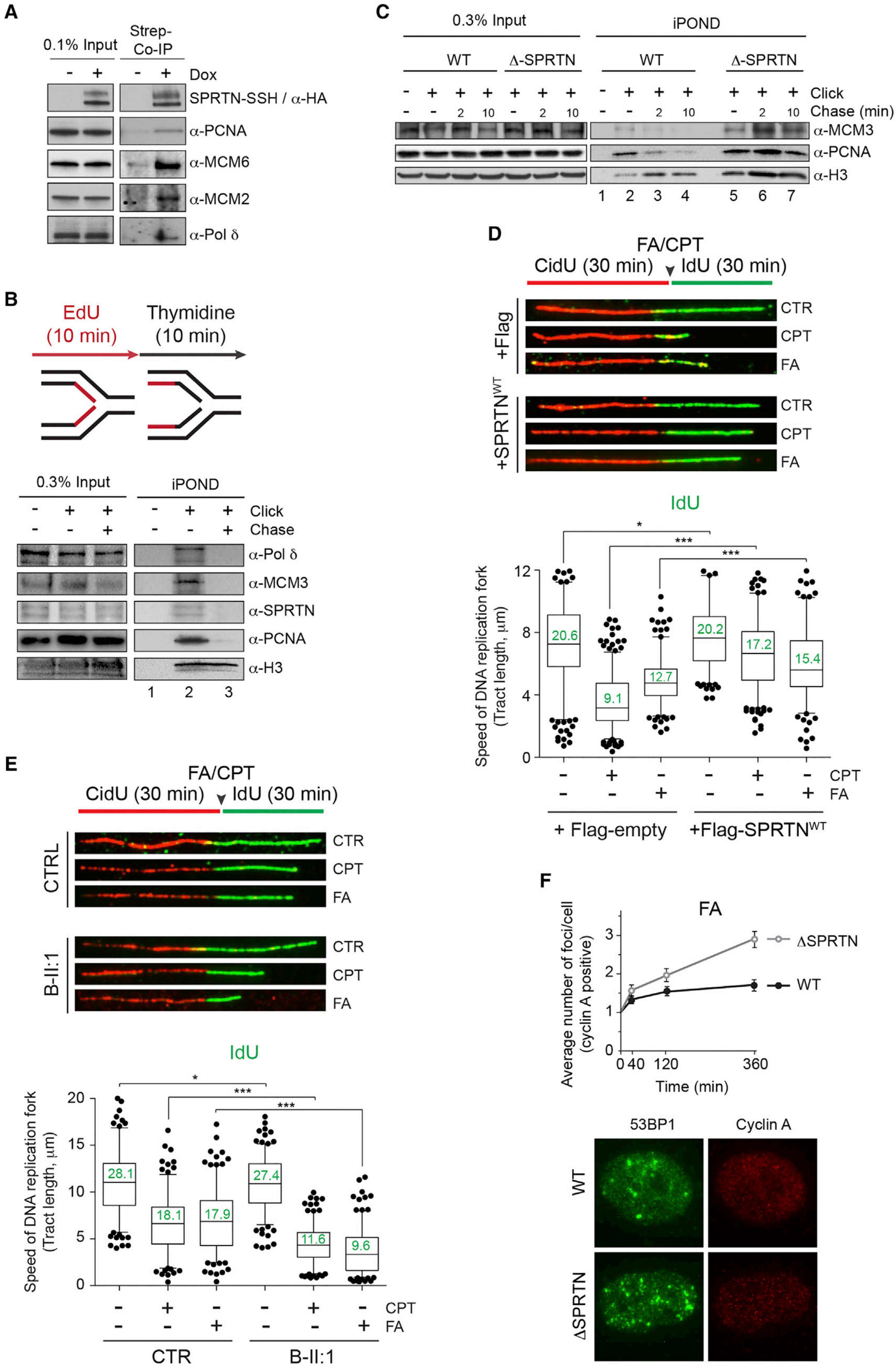

(legend on next page)

formation of 53BP1 foci, a recognized marker for DSBs, over a period of 6 hr by immunofluorescence microscopy in fixed cells. Cyclin A staining was used as a marker for S-/G2-phase-positive cells. The formation of 53BP1 foci was induced in both cyclin A positive and negative cells after FA or CPT treatment, further confirming DPC-induced genotoxicity (Figures 6F and S6G–S6I). However, the average number of 53BP1 foci per cell was 2- to 3-fold increased after FA or CPT treatment in cyclin A-positive Δ-SPRTN HeLa cells compared to control HeLa cells (Figures 6F and S6G). In contrast, the number of 53BP1 foci in cyclin A-negative cells was the same in both Δ-SPRTN and control cells (Figures S6H and S6I). These results suggest that SPRTN prevents DPC-induced DSBs during DNA synthesis.

DISCUSSION

We have revealed mechanistic insights into SPRTN protease activity for DPC repair and established SPRTN as a DNA replication-coupled protease (Figure 7). Our work identifies the missing protease involved in DNA replication and in the processing of Topoisomerase 1 and 2 crosslinks (Ashour et al., 2015; Duxin et al., 2014; Gao et al., 2014; Interthal and Champoux, 2011). Biochemical characterization of SPRTN mutations from RJALS patients shows that RJALS is caused by a defect in SPRTN protease activity, rendering it unable to process DPCs during DNA replication and therefore leading to DNA replication stress, one of the main causes of genome instability and cancer.

DNA Replication-Coupled SPRTN Proteolysis

Our finding is in agreement with recent biochemical data in *Xenopus* egg extract, showing that DNA replication-coupled proteolysis is essential for DPC removal (Duxin et al., 2014), a model proposed by the Sancar laboratory (Reardon et al., 2006). However, the enzyme for this pathway remained unknown until now, when we show that SPRTN is an active protease and constitutive part of the replisome. Taking into consideration that DPCs are ubiquitous and that virtually any protein present in close proximity to DNA can be crosslinked, the proteolytic activity of SPRTN in DNA replication is essential. Cell-cycle dynamics of SPRTN expression support our hypothesis, with SPRTN being absent in G0 of T24-cells, and emerging upon entry into S-phase. This is in agreement with published data, showing that the level of SPRTN is downregulated in cells in G1 phase by the E3-ubiquitin ligase APC-complex and upregulated as cells enter S-phase (Mosbech et al., 2012). We therefore propose this to be a DNA repair pathway for the removal of DPCs in proliferative cells. We cannot exclude a potential role for SPRTN in non-proliferative cells, but if it exists, this function is not associated with DNA replication-coupled DPC repair.

DNA-Protein Crosslink Repair

Our work addresses an emerging question in the field of DNA repair: how are DPCs removed from chromatin? Despite the frequent occurrence of DPCs, we poorly understand the process of DPC repair (Ide et al., 2015). It is believed that DPC repair partially depends on two canonical DNA repair pathways, NER and HR (Barker et al., 2005; Nakano et al., 2007). However, both repair pathways are limited by the fact that NER can only act on smaller protein crosslinks, not larger than 11 kDa, and HR relies on DSB formation that leads to recombinogenic events and associated genomic instability. As a consequence, other repair mechanisms should be involved in DPC processing and maintenance of genomic stability. Indeed, DPC accumulation is neither observed upon inactivation of key NER and HR players nor the Fanconi anemia pathway (Figure S2A), suggesting that the SPRTN-dependent DPC repair pathway is the main, specialized pathway for DPC repair in human cells. Early embryonic lethality of SPRTN knockout mice (Maskey et al., 2014), and the identification of SPRTN as one of the essential genes in the human genome (Hart et al., 2015; Wang et al., 2015), further supports the conclusion that SPRTN is a critical component of the unique DNA repair system, namely, DPC repair pathway.

Characterization of RJALS Patient Mutations

We demonstrated that the protease activity of patient mutation $SPRTN^{Y117C}$ is severely affected on all tested substrates. The reason why the Y117C mutation affects SPRTN enzymatic activity is not clear. Sequence comparisons show that this residue is not strictly conserved among SPRTN homologs (Figure S7A), although it is commonly hydrophobic, even among the wider zincin family. The close proximity of Y117 to the catalytic center, located only two residues downstream of the second potential Zn-binding histidine (H115), and the non-conservative nature of the substitution indicate that the variant may be unable to form the active site with the correct geometry. This is possibly a result of steric effects involving the positioning of the end of the HEXXH-containing helix and the subsequent loop containing the third potential Zn^{2+} ligand.

The other pathogenic mutation, SPRTN-ΔC, is proteolytically active toward itself (auto-cleavage) and histones (Figures 3H

Figure 6. SPRTN Is a Part of the DNA Replication Machinery and Regulates DNA Replication Fork Progression

(A) CoIP of SPRTN-Strep-Strep-His (SSH) from Flp-In HEK293 cells stably expressing SPRTN upon Dox induction showing association with replisome proteins.

(B) Schematic of the iPOND approach (upper image). iPOND is illustrating that SPRTN moves with the replisome. HEK293 cells were treated with EdU for 10 min to label nascent DNA (lane 2) and then chased with thymidine where indicated (mature DNA, lane 3).

(C) iPOND showing increased retention of replisome proteins on mature DNA in Δ-SPRTN HeLa cells (lane 6 and 7).

(D) DNA fiber assay in HeLa cells after overexpression of FLAG-$SPRTN^{WT}$ or empty FLAG-vector. The representative DNA fibers are shown (upper image). The quantification of IdU-labeled tract length in the presence of DPC-inducing agents (50 μM FA, 25 nM CPT) is shown. The numbers (in green) are mean values of tract length in kilobases.

(E) DNA fiber assay in RJALS LCLs (B-II:1) and control LCLs after FA or CPT treatment performed as in (D).

(F) Quantification of 53BP1 foci in cyclin A positive HeLa WT or Δ-SPRTN cells after FA treatment (50 μM). The data represent fold-change in 53BP1 foci compared to untreated cells (time point 0) (upper image). The representative micrographs of 53BP1 foci in Cyclin A positive cell at 6 hr after FA treatment are shown (lower image). Mean ± SEM, n = 3.

See also Figure S6.

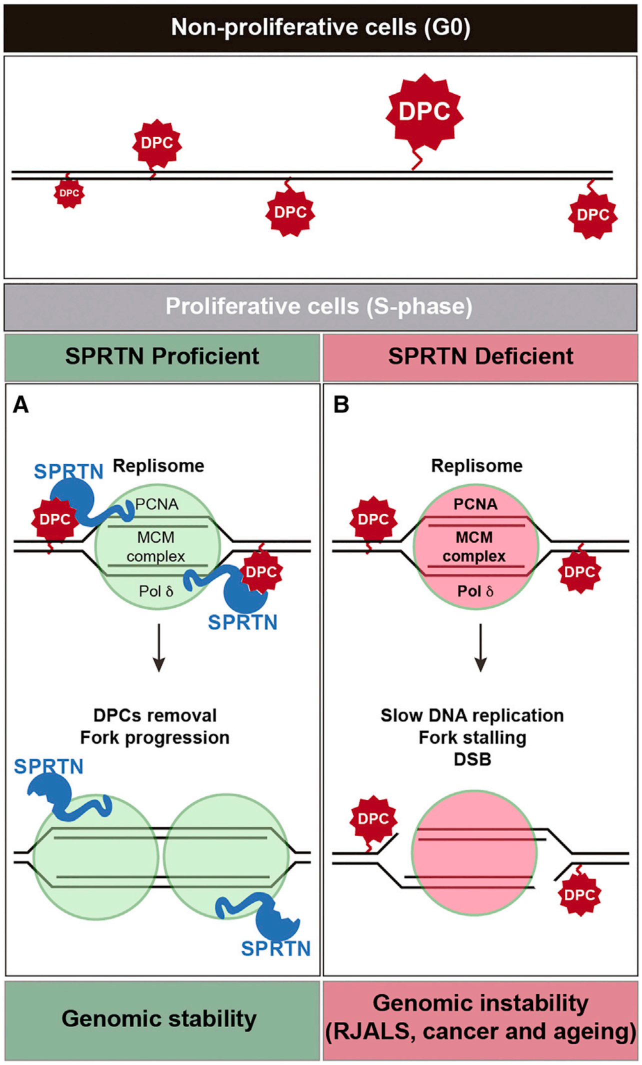

Figure 7. Model of DNA Replication-Coupled DPC Repair

SPRTN is a constitutive part of the replisome and cleaves DPCs during replication fork progression. SPRTN protease protects proliferative cells from DPC-induced cytotoxicity (left). SPRTN deficiency (SPRTN mutations in RJALS, SPRTN haploinsufficient mice) causes stalling of the DNA replication fork due to pathological accumulation of DPCs, which in turn leads to DSBs and genomic instability (right).

and 4D), but is severely impaired in processing Topo1 and Topo2α (Figure 4G). The sensitivity of SPRTN-depleted cells to CPT could not be completely restored by SPRTN-ΔC, suggesting that this patient mutation, although proteolytically active, cannot properly process certain substrates, such as Topo1 and Topo2. One reason could be that the C-terminal part is important for binding particular substrates and is involved in SPRTN substrate specificity. The increased levels of DPCs in RJALS patient cells, and the inability of DNA replication in RJALS cells to cope with the induction of non-enzymatic (FA) or enzymatic (CPT) DPCs, demonstrates that DPCs such as Topo1 and Topo2α, and most probably many others, are the main cause of RJALS syndrome. We propose that RJALS is a human disease linked to defective DPC repair pathway.

Comparison between SPRTN and Wss1

Bioinformatic analysis suggests that SPRTN and Wss1 have a common ancestor (Stingele et al., 2015). While it is true that both enzymes are distantly related to the zinc metalloprotease superfamily, the two sequences can only be aligned over a relatively short 95 amino acid (aa) residue region with 24% sequence identity (comparing the human and *S. cerevisiae* enzymes) (Figure S7B). This common region includes the conserved HEXXH motif, shared among a wider set of enzymes with diverse functions, which provides the platform for Zn^{2+} binding via two histidines, with a neighboring glutamate residue thought to play a role in catalysis (Hooper, 1994). Beside this common core, no other regions of sequence similarity could be found. Wss1 and SPRTN show similar enzymatic properties, but their cellular function is partially uncoupled. SPRTN interacts with DNA replication machinery and binds ubiquitinated substrates via its Ub-binding domains, while Wss1 binds and processes SUMOylated substrates via its SUMO-interacting motifs (Balakirev et al., 2015). Inactivation of SPRTN causes a massive accumulation of total DPCs in proliferative human cells, including Topo1, Topo2α, and histones. In contrast, yeast cells lacking Wss1 do not accumulate total DPCs and do not show hypersensitivity to CPT (Stingele et al., 2014).

EXPERIMENTAL PROCEDURES

DNA-Protein Crosslinks Isolation

DPCs were detected using a modified rapid approach to DNA adduct recovery (RADAR) assay (Kiianitsa and Maizels, 2013). The patient material used in this study (LCLs) was approved by the Human Research Ethics Committee of the Royal Children's Hospital and the Oxford Research Ethics Committee Oxford Radcliffe Biobank (Ref.: 15/A156), University of Oxford. See the Supplemental Experimental Procedures for details.

DNA-Protein Crosslinks Detection

Specific DPCs were detected using a vacuum slot-blot manifold (Bio-Rad) followed by immunodetection. DPCs were visualized using the Bio-Rad ChemiDoc XRS Plus Analyzer. See the Supplemental Experimental Procedures for details.

Protein Purification

For overexpression in *E. coli* cells, SPRTN constructs were cloned in to either the pNIC-ZB vector (full-length SPRTN-WT, SPRTN-E112A, and SPRTN-Y117C), or pNIC28-Bsa4 vector (all other truncated constructs). For purification of full-length constructs containing a TEV cleavable Z-basic-his tag, cells lysates were applied to a Ni-sepharose IMAC gravity flow column. Elution fractions were applied directly to a 5 mL HiTrap SP HP column, washed, and eluted. The purification tag was cleaved with the addition of 1:20 mass ratio of His-tagged TEV protease during overnight dialysis. Protein identities were verified by LC/ESI-TOF mass spectrometry. See the Supplemental Experimental Procedures for details.

Fluorescence Polarization DNA Binding Assays and DNA Probe Annealing

See the Supplemental Experimental Procedures for details.

Generation of CRISPR/Cas9 SPRTN Partial Knockout HeLa Cells

See the Supplemental Experimental Procedures for details.

SPRTN Substrate Cleavage Assay

SPRTN enzymatic reactions were performed in 150 mM NaCl and 25 mM Tris (pH 7.4) in a PCR block at 37°C. The reaction volume was typically 10 μL and contained: *E. coli* purified recombinant SPRTN (1–10 mg/mL solution), substrate (typically 1 mg/mL solution), and 100 bp dsDNA oligonucleotide probe. See the Supplemental Experimental Procedures for details.

Isolation of Proteins On Nascent DNA

See the Supplemental Experimental Procedures for details.

DNA Fiber Assay

The DNA fiber assay was performed as described previously (Lessel et al., 2014). See the Supplemental Experimental Procedures for details.

Mass Spectrometry Analysis

See the Supplemental Experimental Procedures for details.

Quantitative Proteomics and Data Analysis

See the Supplemental Experimental Procedures for details.

Statistical Analysis

See the Supplemental Experimental Procedures for details.

ACCESSION NUMBERS

The accession number for the mass spectrometry raw data reported in this paper is PRIDE: PXD004154 (https://www.ebi.ac.uk/pride/archive/).

SUPPLEMENTAL INFORMATION

Supplemental Information includes Supplemental Experimental Procedures and seven figures and can be found with this article online at http://dx.doi.org/10.1016/j.molcel.2016.09.032.

AUTHOR CONTRIBUTIONS

B.V. performed in vivo and in vitro DPCs analysis, cellular toxicity, and biochemically characterized SPRTN self-cleavage activity. M.P. performed characterization of SPRTN enzymatic activity toward the substrates, identification of cleavage sites, and co-immunoprecipitation (coIP) experiments. J.A.N., H.A., and O.G. purified proteins and performed DNA binding assays. J.F. performed iPOND, and S.H. performed the DNA fiber assay. A.N.S. created stable cell lines. B.V., I.V., R.F., I.T., and B.M.K. performed mass spectrometry and data analysis. R.F. created reagents, G.W.M. provided reagents and analyzed data, and D.J.A. and P.J.L. secured RJALS/SPARTAN patient material. M.P. and J.A.N. analyzed the cleavage products. B.V. and N.D. analyzed DSB formation. B.V. and K.R. designed the majority of the experiments, and B.V., M.P., and K.R. analyzed data and prepared the manuscript. K.R. supervised the project.

ACKNOWLEDGMENTS

This work was supported by the Medical Research Council programme grant (MC_PC_12001/1) to K.R., the Goodger Scholarship of the University of Oxford to S.H., the John Fell Fund 133/075 and the Wellcome Trust grant 097813/Z/11/Z to B.M.K, the Kennedy Trust Fund to R.F. and B.M.K, and the NHMRC Career Development Fellowship (GNT1032364) to P.L. This work was also supported by Victorian State Government Operational Infrastructure Support and Australian Government NHMRC IRIISS. O.G., J.A.N., and H.A. are funded by the SGC, a registered charity (number 1097737) that receives funds from AbbVie, Bayer Pharma AG, Boehringer Ingelheim, Canada Foundation for Innovation, Eshelman Institute for Innovation, Genome Canada, Innovative Medicines Initiative (EU/EFPIA) (ULTRA-DD grant no. 115766), Janssen, Merck & Co., Novartis Pharma AG, Ontario Ministry of Economic Development and Innovation, Pfizer, São Paulo Research Foundation-FAPESP, Takeda, and the Wellcome Trust (106169/ZZ14/Z).

Received: May 19, 2016
Revised: July 15, 2016
Accepted: September 22, 2016
Published: October 27, 2016

REFERENCES

Ashour, M.E., Atteya, R., and El-Khamisy, S.F. (2015). Topoisomerase-mediated chromosomal break repair: an emerging player in many games. Nat. Rev. Cancer *15*, 137–151.

Balakirev, M.Y., Mullally, J.E., Favier, A., Assard, N., Sulpice, E., Lindsey, D.F., Rulina, A.V., Gidrol, X., and Wilkinson, K.D. (2015). Wss1 metalloprotease partners with Cdc48/Doa1 in processing genotoxic SUMO conjugates. eLife *4*, 4.

Barker, S., Weinfeld, M., and Murray, D. (2005). DNA-protein crosslinks: their induction, repair, and biological consequences. Mutat. Res. *589*, 111–135.

Centore, R.C., Yazinski, S.A., Tse, A., and Zou, L. (2012). Spartan/C1orf124, a reader of PCNA ubiquitylation and a regulator of UV-induced DNA damage response. Mol. Cell *46*, 625–635.

Connelly, J.C., and Leach, D.R. (2004). Repair of DNA covalently linked to protein. Mol. Cell *13*, 307–316.

Davis, E.J., Lachaud, C., Appleton, P., Macartney, T.J., Näthke, I., and Rouse, J. (2012). DVC1 (C1orf124) recruits the p97 protein segregase to sites of DNA damage. Nat. Struct. Mol. Biol. *19*, 1093–1100.

de Graaf, B., Clore, A., and McCullough, A.K. (2009). Cellular pathways for DNA repair and damage tolerance of formaldehyde-induced DNA-protein crosslinks. DNA Repair (Amst.) *8*, 1207–1214.

Debéthune, L., Kohlhagen, G., Grandas, A., and Pommier, Y. (2002). Processing of nucleopeptides mimicking the topoisomerase I-DNA covalent complex by tyrosyl-DNA phosphodiesterase. Nucleic Acids Res. *30*, 1198–1204.

Duxin, J.P., and Walter, J.C. (2015). What is the DNA repair defect underlying Fanconi anemia? Curr. Opin. Cell Biol. *37*, 49–60.

Duxin, J.P., Dewar, J.M., Yardimci, H., and Walter, J.C. (2014). Repair of a DNA-protein crosslink by replication-coupled proteolysis. Cell *159*, 346–357.

Friedberg, E.C., Aguilera, A., Gellert, M., Hanawalt, P.C., Hays, J.B., Lehmann, A.R., Lindahl, T., Lowndes, N., Sarasin, A., and Wood, R.D. (2006). DNA repair: from molecular mechanism to human disease. DNA Repair (Amst.) *5*, 986–996.

Gao, R., Schellenberg, M.J., Huang, S.Y., Abdelmalak, M., Marchand, C., Nitiss, K.C., Nitiss, J.L., Williams, R.S., and Pommier, Y. (2014). Proteolytic degradation of topoisomerase II (Top2) enables the processing of Top2·DNA and Top2·RNA covalent complexes by tyrosyl-DNA-phosphodiesterase 2 (TDP2). J. Biol. Chem. *289*, 17960–17969.

Ghosal, G., Leung, J.W., Nair, B.C., Fong, K.W., and Chen, J. (2012). Proliferating cell nuclear antigen (PCNA)-binding protein C1orf124 is a regulator of translesion synthesis. J. Biol. Chem. *287*, 34225–34233.

Hart, T., Chandrashekhar, M., Aregger, M., Steinhart, Z., Brown, K.R., MacLeod, G., Mis, M., Zimmermann, M., Fradet-Turcotte, A., Sun, S., et al. (2015). High-resolution CRISPR screens reveal fitness genes and genotype-specific cancer liabilities. Cell *163*, 1515–1526.

Hooper, N.M. (1994). Families of zinc metalloproteases. FEBS Lett. *354*, 1–6.

Ide, H., Shoulkamy, M.I., Nakano, T., Miyamoto-Matsubara, M., and Salem, A.M. (2011). Repair and biochemical effects of DNA-protein crosslinks. Mutat. Res. *711*, 113–122.

Ide, H., Nakano, T., Shoulkamy, M.I., and Salem, A.M. (2015). Formation, repair, and biological effects of DNA-protein cross-link damage. In Advances in DNA Repair (Amst.), C.C. Chen, ed. (InTech) pp. 44–80.

Interthal, H., and Champoux, J.J. (2011). Effects of DNA and protein size on substrate cleavage by human tyrosyl-DNA phosphodiesterase 1. Biochem. J. *436*, 559–566.

Jackson, S.P., and Bartek, J. (2009). The DNA-damage response in human biology and disease. Nature *461*, 1071–1078.

Kiianitsa, K., and Maizels, N. (2013). A rapid and sensitive assay for DNA-protein covalent complexes in living cells. Nucleic Acids Res. *41*, e104.

Kim, M.S., Machida, Y., Vashisht, A.A., Wohlschlegel, J.A., Pang, Y.P., and Machida, Y.J. (2013). Regulation of error-prone translesion synthesis by Spartan/C1orf124. Nucleic Acids Res. *41*, 1661–1668.

Kuo, H.K., Griffith, J.D., and Kreuzer, K.N. (2007). 5-Azacytidine induced methyltransferase-DNA adducts block DNA replication in vivo. Cancer Res. *67*, 8248–8254.

Lessel, D., Vaz, B., Halder, S., Lockhart, P.J., Marinovic-Terzic, I., Lopez-Mosqueda, J., Philipp, M., Sim, J.C., Smith, K.R., Oehler, J., et al. (2014). Mutations in SPRTN cause early onset hepatocellular carcinoma, genomic instability and progeroid features. Nat. Genet. *46*, 1239–1244.

Lindahl, T. (1993). Instability and decay of the primary structure of DNA. Nature *362*, 709–715.

Lindahl, T., and Wood, R.D. (1999). Quality control by DNA repair. Science *286*, 1897–1905.

Maede, Y., Shimizu, H., Fukushima, T., Kogame, T., Nakamura, T., Miki, T., Takeda, S., Pommier, Y., and Murai, J. (2014). Differential and common DNA repair pathways for topoisomerase I- and II-targeted drugs in a genetic DT40 repair cell screen panel. Mol. Cancer Ther. *13*, 214–220.

Maskey, R.S., Kim, M.S., Baker, D.J., Childs, B., Malureanu, L.A., Jeganathan, K.B., Machida, Y., van Deursen, J.M., and Machida, Y.J. (2014). Spartan deficiency causes genomic instability and progeroid phenotypes. Nat. Commun. *5*, 5744.

Mosbech, A., Gibbs-Seymour, I., Kagias, K., Thorslund, T., Beli, P., Povlsen, L., Nielsen, S.V., Smedegaard, S., Sedgwick, G., Lukas, C., et al. (2012). DVC1 (C1orf124) is a DNA damage-targeting p97 adaptor that promotes ubiquitin-dependent responses to replication blocks. Nat. Struct. Mol. Biol. *19*, 1084–1092.

Nakano, T., Morishita, S., Katafuchi, A., Matsubara, M., Horikawa, Y., Terato, H., Salem, A.M., Izumi, S., Pack, S.P., Makino, K., and Ide, H. (2007). Nucleotide excision repair and homologous recombination systems commit differentially to the repair of DNA-protein crosslinks. Mol. Cell *28*, 147–158.

Ramadan, K., Halder, S., Wiseman, K., and Vaz, B. (2016). Strategic role of the ubiquitin-dependent segregase p97 (VCP or Cdc48) in DNA replication. Chromosoma, Published online April 18, 2016. http://dx.doi.org/10.1007/s00412-016-0587-4.

Reardon, J.T., Cheng, Y., and Sancar, A. (2006). Repair of DNA-protein cross-links in mammalian cells. Cell Cycle *5*, 1366–1370.

Salem, A.M., Nakano, T., Takuwa, M., Matoba, N., Tsuboi, T., Terato, H., Yamamoto, K., Yamada, M., Nohmi, T., and Ide, H. (2009). Genetic analysis of repair and damage tolerance mechanisms for DNA-protein cross-links in Escherichia coli. J. Bacteriol. *191*, 5657–5668.

Shi, Y., Lan, F., Matson, C., Mulligan, P., Whetstine, J.R., Cole, P.A., Casero, R.A., and Shi, Y. (2004). Histone demethylation mediated by the nuclear amine oxidase homolog LSD1. Cell *119*, 941–953.

Shoulkamy, M.I., Nakano, T., Ohshima, M., Hirayama, R., Uzawa, A., Furusawa, Y., and Ide, H. (2012). Detection of DNA-protein crosslinks (DPCs) by novel direct fluorescence labeling methods: distinct stabilities of aldehyde and radiation-induced DPCs. Nucleic Acids Res. *40*, e143.

Stingele, J., and Jentsch, S. (2015). DNA-protein crosslink repair. Nat. Rev. Mol. Cell Biol. *16*, 455–460.

Stingele, J., Schwarz, M.S., Bloemeke, N., Wolf, P.G., and Jentsch, S. (2014). A DNA-dependent protease involved in DNA-protein crosslink repair. Cell *158*, 327–338.

Stingele, J., Habermann, B., and Jentsch, S. (2015). DNA-protein crosslink repair: proteases as DNA repair enzymes. Trends Biochem. Sci. *40*, 67–71.

Tretyakova, N.Y., Groehler, A., 4th, and Ji, S. (2015). DNA-protein cross-links: formation, structural identities, and biological outcomes. Acc. Chem. Res. *48*, 1631–1644.

Wang, T., Birsoy, K., Hughes, N.W., Krupczak, K.M., Post, Y., Wei, J.J., Lander, E.S., and Sabatini, D.M. (2015). Identification and characterization of essential genes in the human genome. Science *350*, 1096–1101.

Zhang, A., Lyu, Y.L., Lin, C.P., Zhou, N., Azarova, A.M., Wood, L.M., and Liu, L.F. (2006). A protease pathway for the repair of topoisomerase II-DNA covalent complexes. J. Biol. Chem. *281*, 35997–36003.

Molecular Cell, Volume *64*

Supplemental Information

Metalloprotease SPRTN/DVC1 Orchestrates Replication-Coupled DNA-Protein Crosslink Repair

Bruno Vaz, Marta Popovic, Joseph A. Newman, John Fielden, Hazel Aitkenhead, Swagata Halder, Abhay Narayan Singh, Iolanda Vendrell, Roman Fischer, Ignacio Torrecilla, Neele Drobnitzky, Raimundo Freire, David J. Amor, Paul J. Lockhart, Benedikt M. Kessler, Gillies W. McKenna, Opher Gileadi, and Kristijan Ramadan

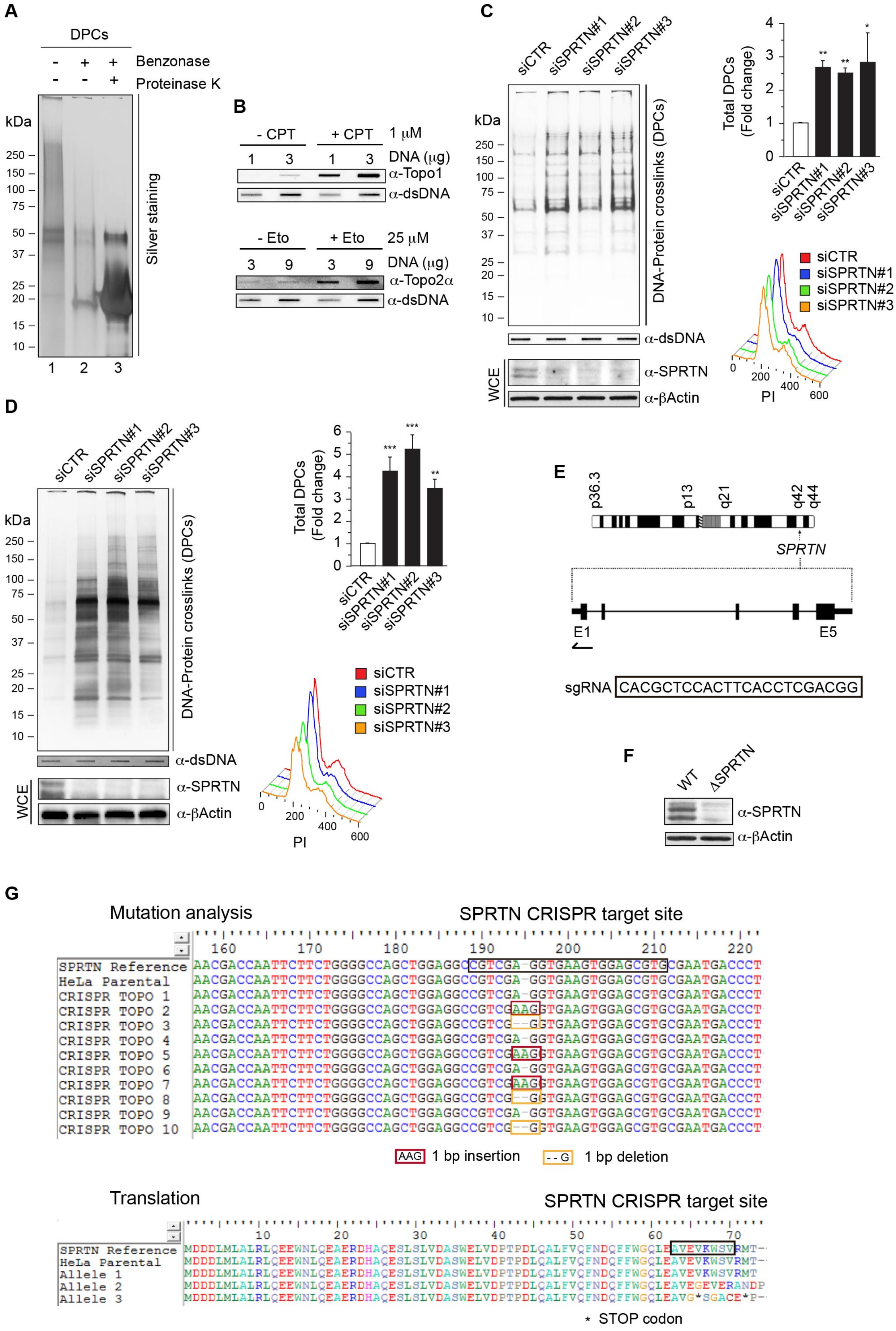

Figure S1 (referred to Figure 1)

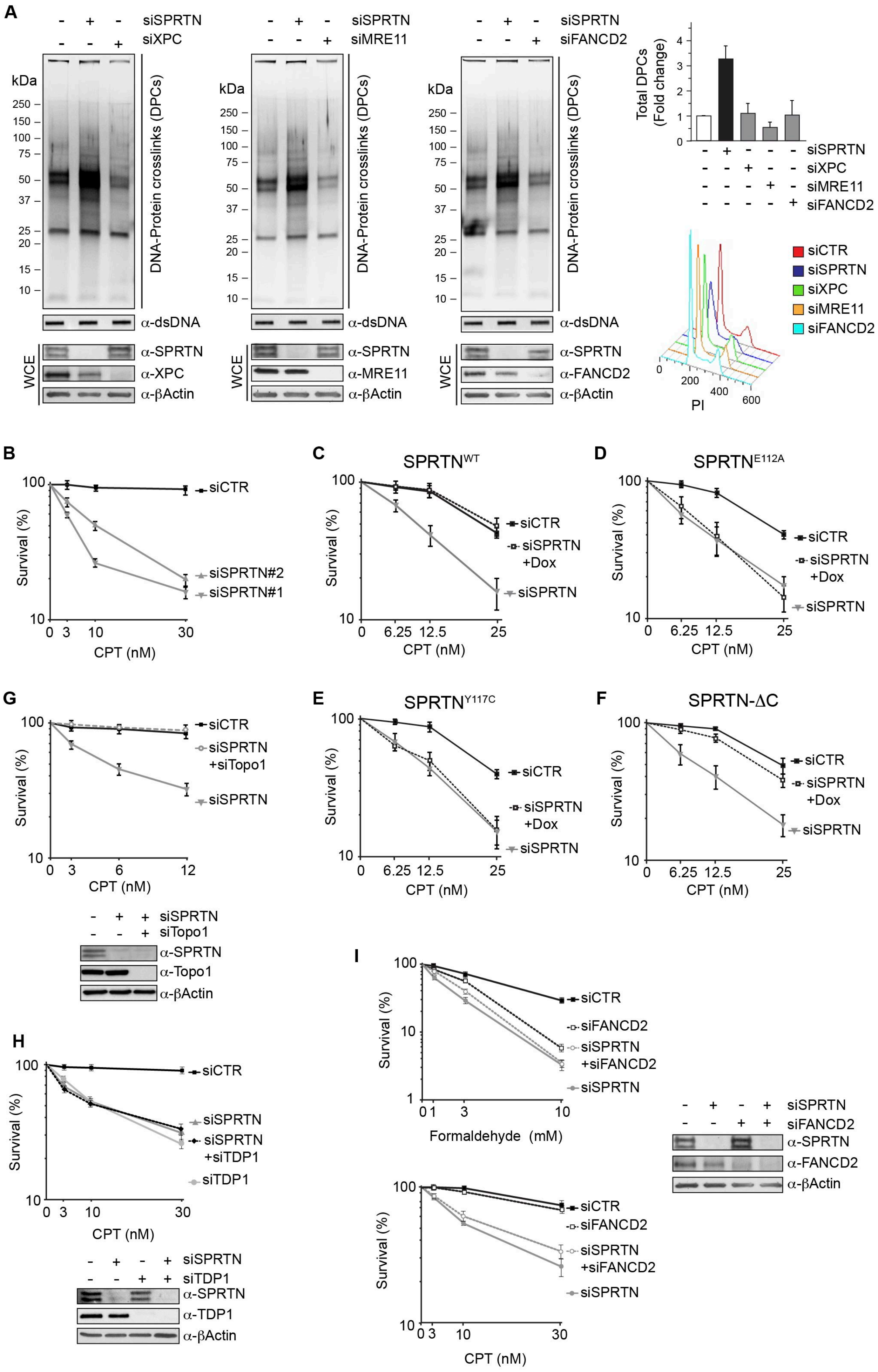

Figure S2 (referred to Figure 1 and 2)

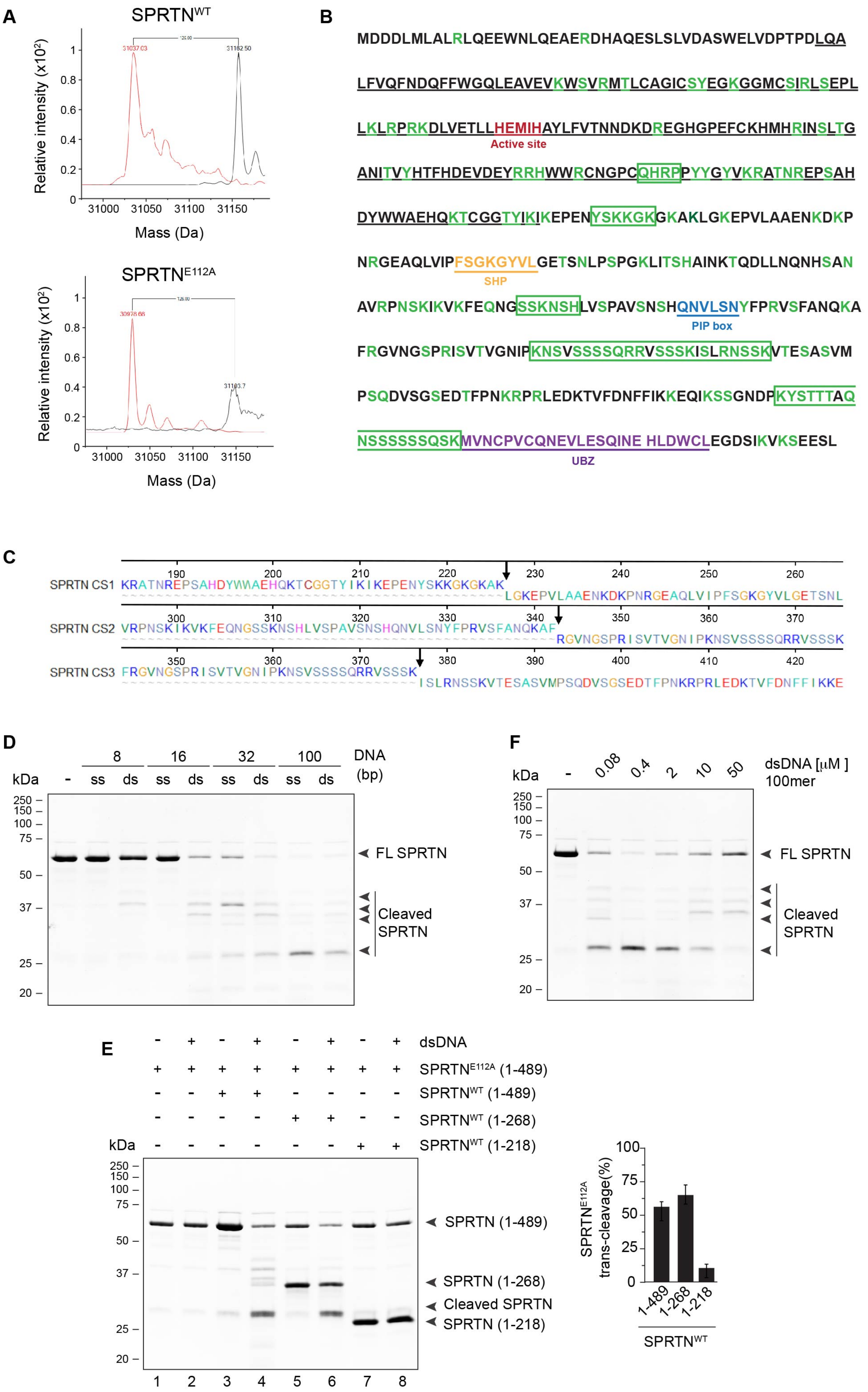

Figure S3 (referred to Figure 3)

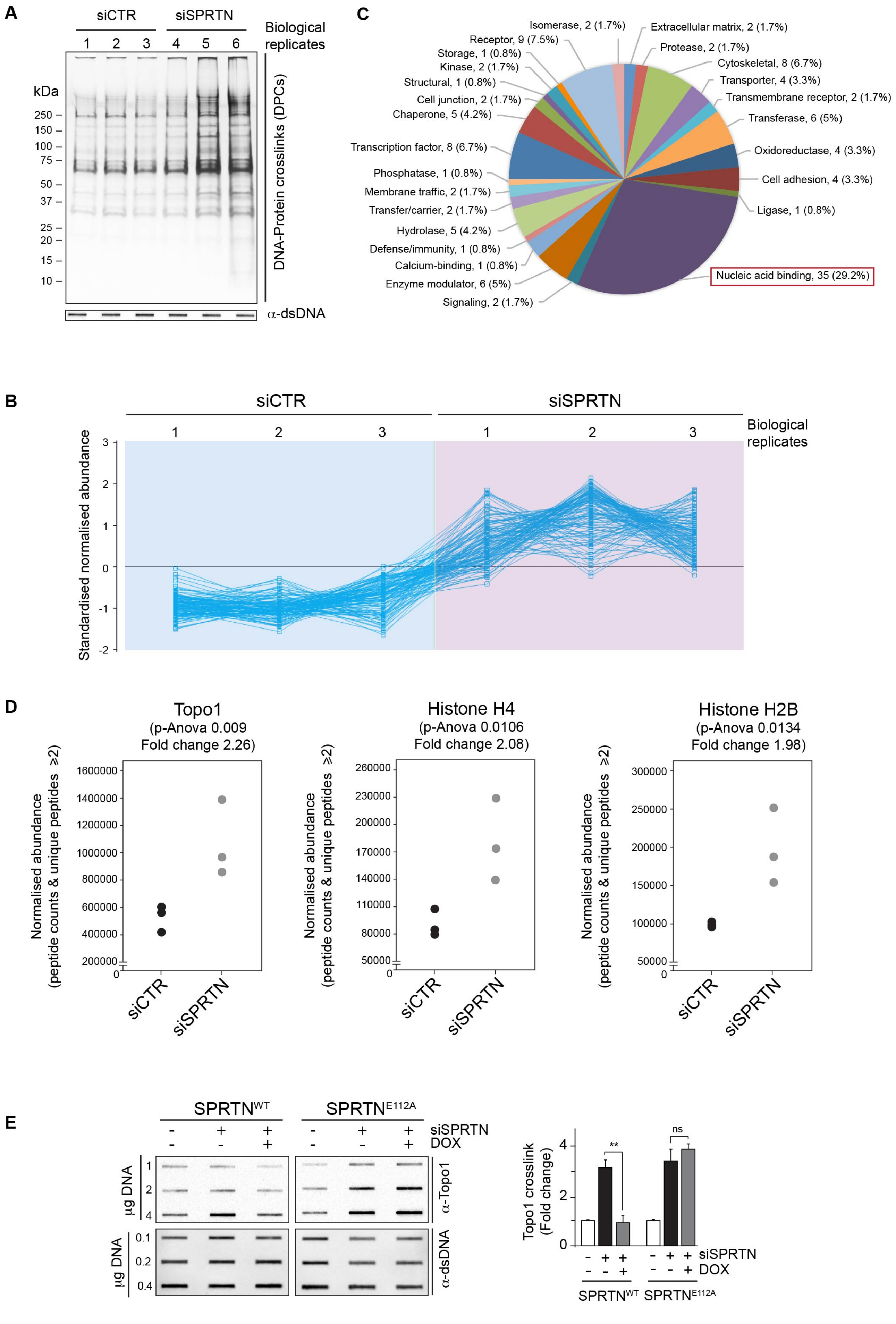

Figure S4 (referred to Figure 4)

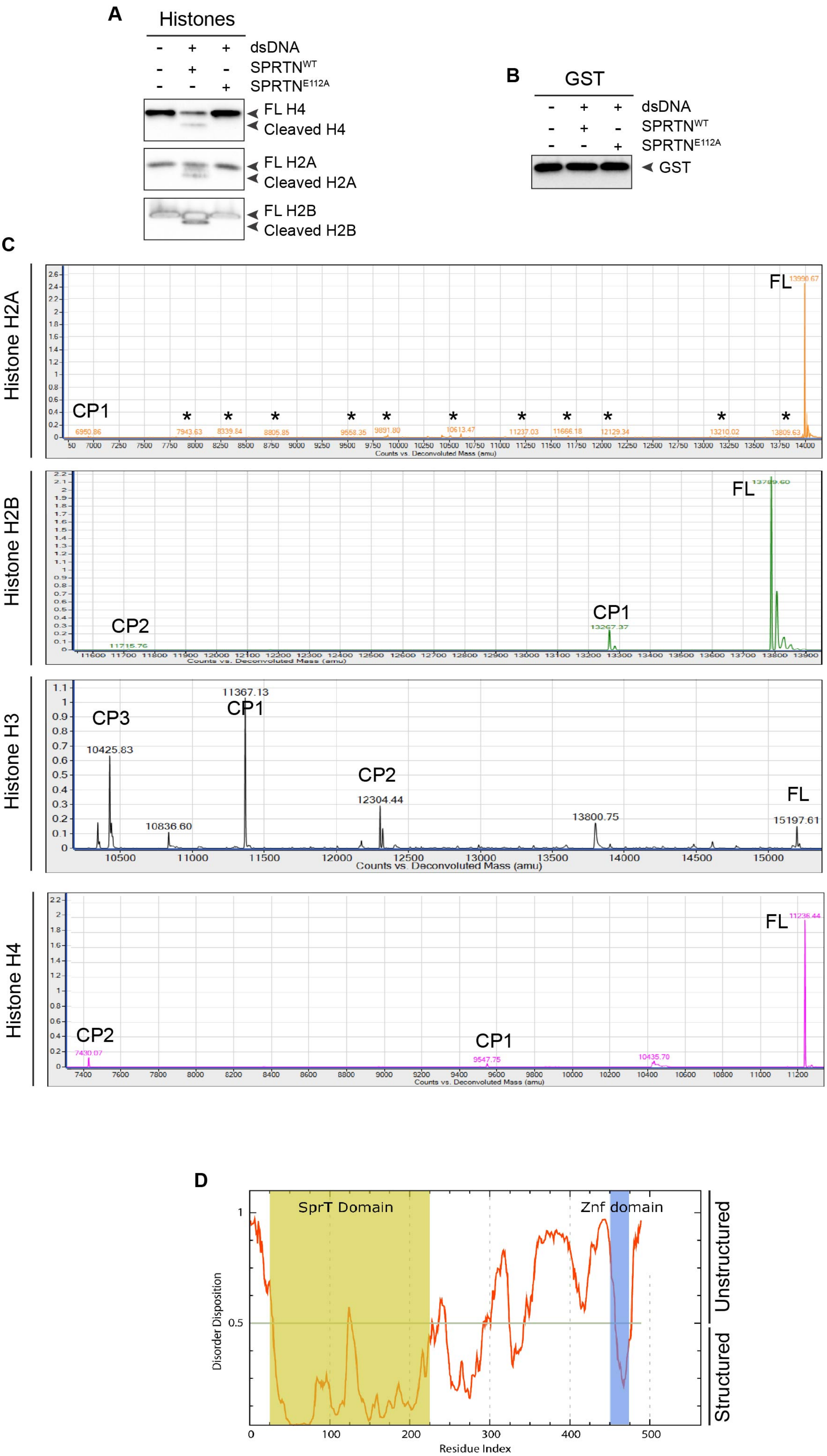

Figure S5 (referred to Figure 4)

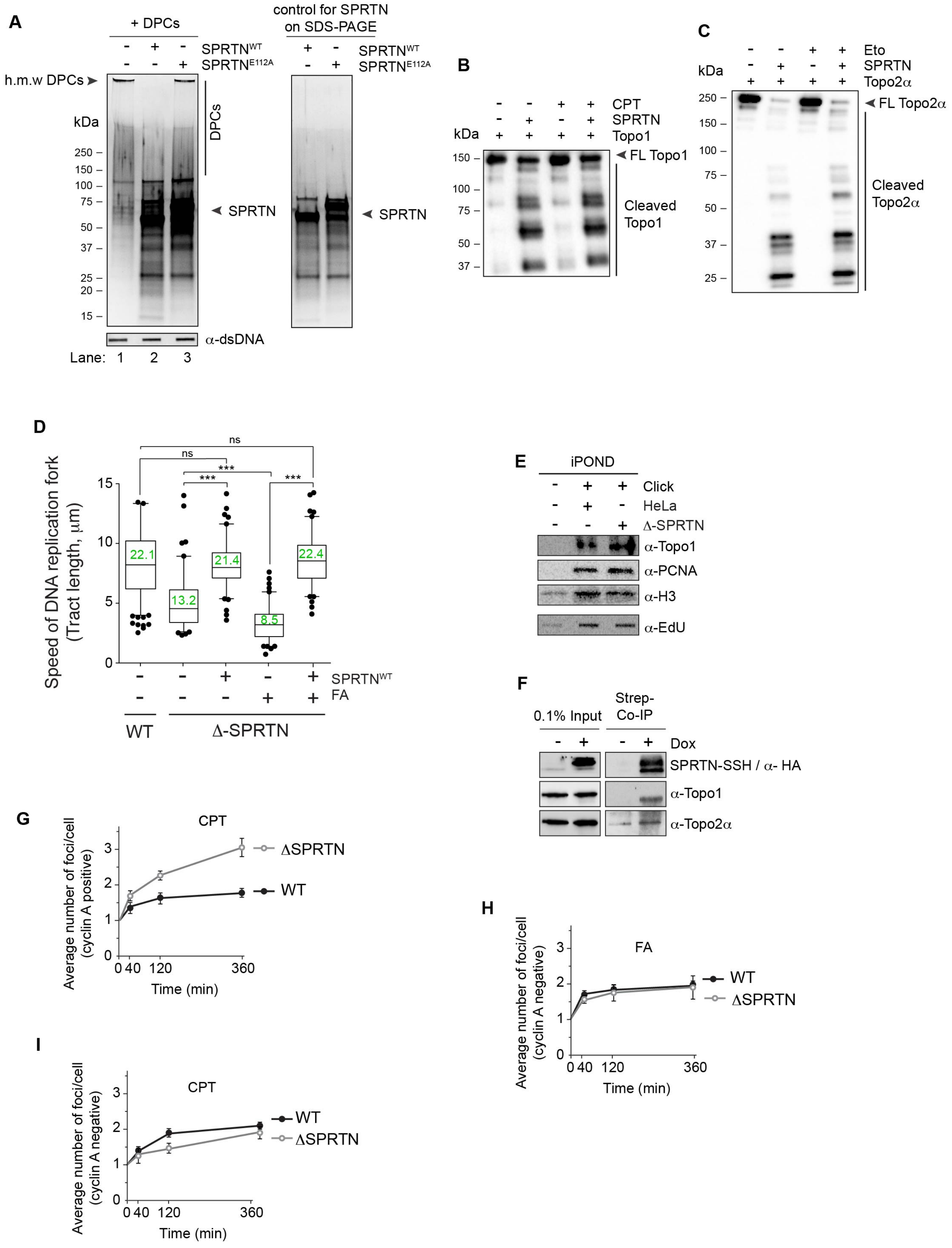

Figure S6 (referred to Figure 4 and 6)

A

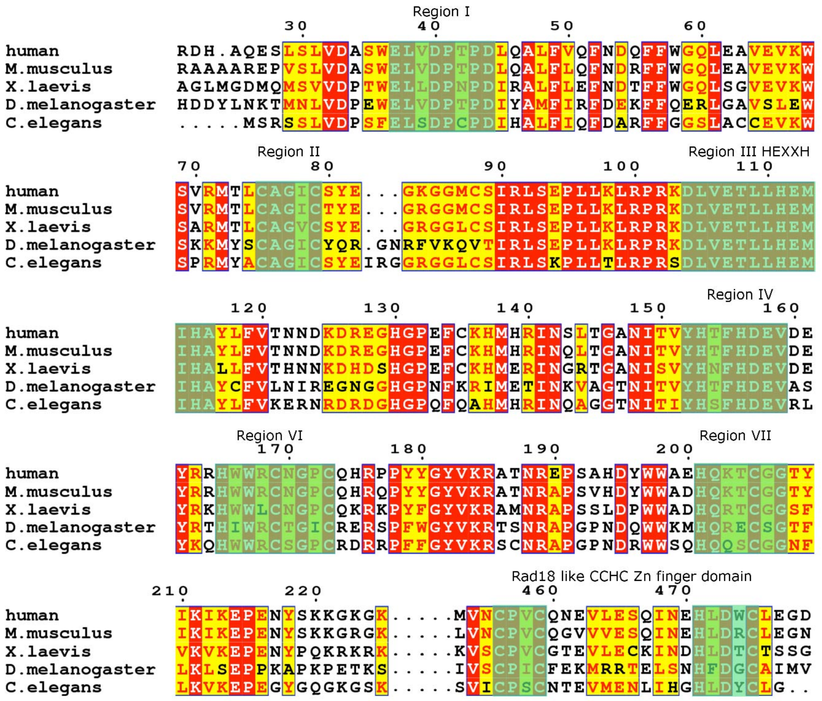

B

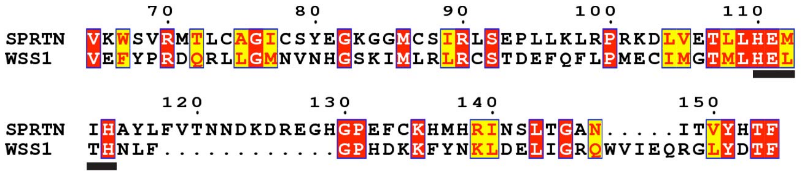

C

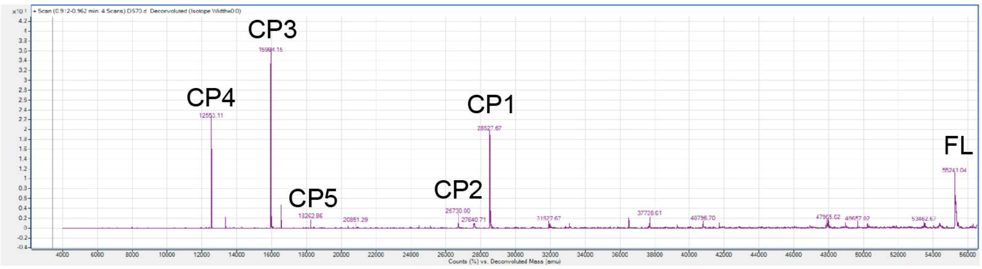

Figure S7 (referred to Figures 7 and 3)

SUPPLEMENTRY FIGURE LEGENDS

Figure S1. SPRTN prevents accumulation of DNA-protein crosslinks (DPCs). Related to Figure 1. (A) Total DPCs isolated from HEK293T cells (lane 1), and after benzonase (30 min, lane 2) or proteinase K digestion (3h, lane 3) at 55 ˚C, resolved with SDS-PAGE and visualised using silver staining. (B) Analysis of enzymatic DPCs by slot-blot; Top1-ccs after CPT treatment (upper panel) and Top2-ccs after ETO treatment (lower panel). dsDNA as a loading control for DPC isolates before benzonase treatment (C) Depletion of SPRTN in HeLa cells with three different siRNAs causes accumulation of total DPCs. Total amount of DPCs was quantified and expressed as a fold change (n = 3). Corresponding cell cycle profiles for each sample (PI; propidium iodide). WCE; whole cell extract. (D) Depletion of SPRTN in HEK293 cells with three different siRNAs leads to a significant accumulation of total DPCs as in C. Total amount of DPCs was quantified and expressed as a fold change (n = 3). WCE; whole cell extract. Cell cycle progression was monitored by propidium iodide (PI) (right panels). (E) Schematics of CRISPR/Cas9 SPRTN partial knock-out in HeLa cells (Δ-SPRTN). The sgRNA sequence targeted the first exon of *SPRTN*. (F) CRISPR partial knock-out was confirmed by western blot with α-SPRTN antibody. (G) Confirmation of SPRTN partial knock-out in HeLa cells by genomic DNA sequencing. Ten parental and 25 CRISPR clones were sequenced. The CRISPR/Cas9 plasmid induced either a 1bp deletion or 1bp insertion, leading to a frameshift and introduction of a premature stop codon. Out of 25 CRISPR clones, 17 were altered while the rest remained unchanged indicating partial knock-out of SPRTN (sequences from 10 clones that show a representative distribution of mutations are shown).

Figure S2. SPRTN protects cells from the DPC-inducing agents and acts independently of HR, NER, and Fanconi Anemia pathways in DPC removal. Related to Figure 2. (A) Total DPC levels after depletion of SPRTN, XPC, MRE11 or FANCD2 in HeLa cells visualised by silver staining with corresponding slot blots showing equal amount of DNA used for DPC analysis and Western blots for depletion efficiencies. WCE; whole cell extract. Corresponding quantification of DPC levels expressed as a fold change to DPCs levels in HeLa WT cells and cell cycle profiles (PI, propidium iodide) (right panel). (B) Clonogenic survival assays of siRNA control (CTR) or siRNA SPRTN-depleted HeLa cells treated with CPT for 24 hrs. Colonies were analysed after 7 days by automated cell counter (n = 3). (C - F) Cell survival of SPRTN depleted Flp-In HeLa cells after doxycycline-induced expression of $SPRTN^{WT}$ (C), $SPRTN^{E112A}$ (D), $SPRTN^{Y117C}$ (E), or SPRTN-ΔC (F) following CPT treatment. (G) Cell survival after SPRTN and Topo1 co-depletion following CPT treatment with corresponding WB to monitor depletion efficiency (lower panel). (H) Cell survival after SPRTN and TDP1 co-depletion following CPT treatment with corresponding WB showing depletion efficiencies (lower panel). (I) Cell survival after SPRTN and FANCD2 co-depletion following FA (upper panel) or CPT (lower panel) treatment with corresponding WB showing depletion efficiencies (right panel).

Figure S3. SPRTN is a zinc and DNA-dependent protease. Related to Figure 3. (A) Comparison of denaturing (shown in red) and native (shown in black) mass spectra for wild type and E112A SPRTN constructs (SPRTN 1-268 amino acid long truncated variant to avoid Zn-binding UBZ-domain). (B) Schematic of the SPRTN protein sequence showing the active site HEXXH (in red), p97 interacting SHP domain, PCNA binding domain (PIP box) and UBZ domain. *In silico* predicted DNA binding residues are shown in green. Four or more consecutive residues with DNA binding capacity are enclosed in green squares. (C) Identification of SPRTN self-cleavage sites. Self-cleavage sites of the SPRTN protease were identified by mass spectrometry analysis. Only cleavage sites (CS) which could be determined with high confidence (unique peptide matches, ppm = 100) are shown (CS 1-3) (See also Figure S7C). (D) Characterization of SPRTN auto-cleavage activity. *In vitro* enzymatic reactions were performed for 2 hours at 37˚C with purified SPRTN^{WT} and DNA probes of different lengths and visualized on gels by Coomassie Blue staining. (E) SPRTN self-cleavage occurs in trans. The enzymatic dead protein SPRTN^{E112A} was incubated with the full length or different C-terminal truncations of the SPRTN^{WT} protein for 2 hours at 37˚C. Trans-cleavage of SPRTN^{E112A} was quantified and expressed as a percentage of cleavage (n = 3), left panel. (F) Titration of the optimal DNA concentration to induce SPRTN self-cleavage. *In vitro* enzymatic reactions were performed with increasing concentrations of dsDNA (100mer) for 2 hours at 37˚C.

Figure S4. Quantitative mass spectrometry analysis of DNA-protein crosslinks in SPRTN-depleted cells. Related to Figure 4. (A) Total DPCs were isolated in triplicate from siRNA control (CTR) or siRNA SPRTN-depleted (siSPRTN#1) HeLa cells according to the RADAR protocol and were visualized by silver staining on SDS-PAGE gels. Samples were processed for mass spectrometry analysis. (B) Abundance profile of the 114 proteins significantly increased (p-ANOVA <0.05) in siSPRTN#1 as compared to control (n = 3 biological replicates). Proteins selected were identified and label-free quantified with at least two unique peptides using Progenesis IQP software. (C) Pie chart showing protein function classification of the 114 significantly overexpressed proteins in siSPRTN#1, which was performed using PANTHER Classification System (Protein ANalysis THrough Evolutionary Relationships, version 10.0). Proteins were identified and quantified with at least two unique peptides. (D) Increased DNA topoisomerase 1, Histone H4 and Histone H2B association to DNA in SPRTN#1 depleted cells. Normalised abundance of three proteins differentially increased in siSPRTN#1 depleted cells as compared to control (fold change ≥ 1.5; p-ANOVA <0.05). (E) Total DPCs were isolated by the RADAR protocol as in Figure 1A from stable Flip/In HeLa cell lines depleted for endogenous SPRTN by 3' UTR siRNA. Ectopic expression of SPRTN^{WT} or SPRTN^{E112A} was induced by DOX and Topo1 levels in DPCs were analyzed by slot blot using a specific antibody against Topo1. Equal amount of DNA was loaded and confirmed by slot blot followed by immune-detection against dsDNA (lower panel). Total amount of Topo1 was quantified and expressed as a fold change compared to siCTR cells (n = 3, right panel).

Figure S5. SPRTN cleaves histones H2A, H2B and H4. Related to Figure 4. (A) SPRTN^{WT} cleaves core histones *in vitro*. Enzymatic reactions were performed with purified SPRTN proteins as indicated and different histones in the presence of dsDNA for 8 hrs at 37˚C. Proteolytic activity was monitored by Western blot using antibodies against the specific histones. (B) Cytosolic protein glutathione S-transferase (GST) is not cleaved by SPRTN. Enzymatic reactions were performed as described above. (C) Mass spectrometry analysis of histones H2A, H2B, H3 and H4 cleavage products (CP) after SPRTN proteolysis. * denotes unspecific peaks. Peaks that are not labelled as CP had more than one unique peptide hit, thus making it impossible to determine exact cleavage site (see Methods) (D) Disorder propensity plot for human SPRTN with the known domain boundaries overlaid. Values greater than 0.5 are considered to be likely disordered.

Figure S6. SPRTN cleaves Topo1-ccs, Topo2α-ccs, and DPCs of high molecular weight and prevents protein accumulation at the replisome and DSB formation. Related to Figure 6. (A) Left gel: *In vitro* enzymatic reactions of SPRTN cleavage of DPCs. Incubation of DPCs isolated from HeLa cells with SPRTN^{WT}, but not SPRTN^{E112A}, leads to a reduction of high molecular weight (h.m.w.) DPCs, and DPCs above 100 kDa (lane 2 in comparison to lanes 1 and 3). Right gel: analysis of SPRTN WT or E112A on SDS-PAGE/Silver staining to demonstrate that products in lanes 2 and 3 are coming from recombinant SPRTN in the reaction. (B) SPRTN cleaves Topo1 and Topo1-ccs with similar efficiency. Topo1 or Topo1-ccs (+CPT) were immunoprecipitated under denaturing conditions from chromatin fraction of HEK293T cells before or after CPT treatment and in vitro enzymatic reactions were performed as in Figure 4F. (C) SPRTN cleaves Topo2α and Topo2α-ccs (+ETO) with similar efficiency. Topo2α and Topo2α-ccs were immunoprecipitated under denaturing conditions from chromatin fraction of HEK293T cells before or after ETO treatment and in vitro enzymatic reactions were performed as in Figure 4F. (D) Ectopic expression of SPRTN^{WT} rescues DNA replication fork velocity in Δ-SPRTN cells before and after FA treatment (n = 3). Numbers in green indicates mean value of DNA synthesis in kilobases (E) Topo1 protein levels increase at the replisome in Δ-SPRTN cells. iPOND was performed as in Figure 6B. (F) SPRTN forms a complex with Topo1 and Topo2α *in vivo*. Co-IP of SPRTN-SSH tag was performed upon doxycycline induction of SPRTN^{WT} expression in Flp-In 293 cells using StrepTactin beads. (G) Graphic representation of the fold-change in the average 53BP1 foci following treatment with camptothecin (25 nM) in HeLa WT or Δ-SPRTN cells. DSB formation was monitored in cyclin A positive cells, as described in Figure 6F. (H and I) Graphic representation of the fold-change in the average 53BP1 foci following treatment with FA (50 μM, H) or CPT (25 nM, I), respectively in HeLa WT or Δ -SPRTN negative for cyclin A.

Figure S7. SPRTN protein alignment and mass spectrometry analysis of SPRTN auto-cleavage (A) Multiple sequence alignment of SPRTN homologues from a diverse set of multicellular eukaryotes. Regions with dense clusters of strictly conserved residues, which also contain possible consensus motifs for metal binding, are numbered and highlighted in green (I-VII). (B) Pairwise

sequence alignment of human SPRTN and *S. cerevisiae* WSS1 shows low similarities over a short region of the catalytic domain around the HEXXH motif, underlined in black. (C) Mass spectrometry analysis of full length SPRTN-WT auto-cleavage products (CP) identified at least five cleaved products within the C-terminal region of SPRTN.

EXTENDED EXPERIMENTAL PROCEDURES

Chemicals, plasmids and recombinant proteins

Formaldehyde (FA), Camptothecin (CPT), Etoposide (Eto), methylglyoxal, hygromycin, puromycin, 5-Chloro-2′ deoxyuridine (CldU) and 5-Iodo-2′-deoxyuridine (IdU) were purchased from Sigma-Aldrich. Topo1/YFP and Topo2α/GFP plasmid constructs were a kind gift from Prof Sherif El-Khamisy. pcDNA3.1/Flag (Invitrogen) and pCDNA5/FRT/TO-cSSH (Invitrogen) were used in the mammalian expression system and pNIC-ZB vector and pNIC28-Bsa4 were used in the E.coli expression system.

Antibodies

The following primary antibodies were used in this study: anti-SPRTN (rabbit, polyclonal) raised against the N-terminal part (1–240 aa) of SPRTN (home made, dilution 1:1000); anti-SPRTN raised against the C-terminal part of SPRTN (Atlas HPA 025073, dilution 1:1000); anti-Topoisomerase 1 (Bethyl A302-589A, dilution 1:5000); anti–topoisomerase 2α (Bethyl A300-054A, dilution 1:10000); anti-dsDNA (Abcam ab27156, dilution 1:5000); anti-histone 2A (Cell signalling 2578, dilution 1:1000); anti-histone 2B (Cell signalling 2934, dilution 1:1000); anti-histone 3 (Abcam ab1791, dilution 1:1000); anti-histone 4 (dilution 1:1000); anti-PCNA (Abcam ab29, dilution 1:1000); anti-MCM6 (Santa Cruz sc-9843, dilution 1:1000); anti-MCM2 (Cell signalling 4007, dilution 1:1000); anti-Polδ (Abcam ab10362, dilution 1:1000); anti-βActin (abcam ab6276, dilution 1:1000); anti-Lamin B1 (ThermoFisher Scientific PA5-19468, dilution 1:1000); anti-cyclin A (Santa Cruz sc751, dilution 1:1000); anti-cyclin B1 (BD Biosciences 610219, dilution 1:1000); anti-cyclin E (Millipore 05-363, dilution 1:1000); anti-DNA-PK (Cell signalling 12311S, dilution 1:1000) and anti-rat and anti-mouse 5-bromo-2′-deoxyuridine (BrdU) (anti mouse Abcam ab6326, dilution 1:500, and anti rat BD Biosciences 347580, dilution 1:100, respectively). Secondary antibodies used in this study are as follows: anti-mouse horseradish peroxidase (HRP) (Sigma A2304, dilution 1:50000); anti-rabbit HRP (Sigma A05451, dilution 1:50000); Anti-rat Cy3 (Jackson ImmunoResearch 712-116-153, dilution 1:300) and anti-mouse Alexa-488 (Molecular Probes A11001, dilution 1:300).

Cloning and site directed mutagenesis

The I.M.A.G.E. full-length *SPRTN* cDNA clone (IRATp970E1156D, ImaGenes) was cloned into pcDNA3.1 (containing the Flag-tag at the N-terminus) and pCDNA5/FRT/TO-cSSH vector (containing Strep and HA tags at the C-terminus) for expression in a mammalian system. For expression in E. coli, *SPRTN* cDNA clone was cloned into pNIC-ZB vector with N-terminal His and ZB tags (full length $SPRTN^{WT}$, $SPRTN^{E112A}$, and $SPRTN^{Y117C}$) or pNIC28-Bsa4 vector with N-terminal His tag (all other truncated constructs). Site-directed mutagenesis was performed using

mutagenic primers by PCR using AccuPrime Pfx DNA polymerase (Invitrogen) according to the manufacturer instructions. DNA sequence was verified by Source Biosciences sequencing service, Oxford, UK.

Mammalian cells, siRNA and transfection protocols

HeLa, HEK293T and T24 cells were cultured in Dulbecco's modified Eagle's medium with 10% fetal calf serum. Epstein-Barr virus (EBV)-transformed LCLs were cultured in RPMI medium with 15% fetal calf serum. Cells were grown to 50–80% confluence before treatment with CPT, ETO, FA or methylglyoxal at indicated concentrations. siRNA transfections were performed using Lipofectamine RNAiMax reagent (Invitrogen) according to the manufacturer's protocol, and depletion was assayed at 72 h post-transfection. siRNA sequences are as follows:

siSPRTN#1 (GUCAGGAAGUUCUGGUUAA);

siSPRTN#2 (CACGAUGAGGUGGAUGAGUAU);

siSPRTN#3 (AGCCAAUAUAACGGUAUACCA);

siTopo1 (GGUCCCUGUUGAGAAACGA);

siTDP1 (GGAUAUUGCGUUUGGAACA);

siMre11 (GAUAGACAUUAGUCCGGUU)

siXPC (SMARTpool, 40nM) (Dharmacon), and

siFANCD2 (SMARTpool, 40nM) (Dharmacon).

Plasmid transfections were performed using FuGene HD reagent (Promega) according to the manufacturer's protocol, and drug response was assayed at 24-48 h post-transfection. For denaturing immunoprecipitation (IP) of Topo1/YFP and Topo2α/GFP, HEK293T cells were transfected with corresponding recombinant plasmid using polyethyleneimine (PEI) reagent (Tom et al., 2008) and collected for IP after 48h.

Generation of SPRTN/Flp-In T-REx stable cell lines

HeLa and HEK-293 SPRTN/Flp-In T-REx stable cell lines were prepared according to manufacturer's protocol for doxycycline inducible constitutive expression of SPRTN variants (WT, E112A, Y117C and DC). The *SPRTN* cDNA was amplified by PCR and cloned in pCDNA5/FRT/TO-cSSH vector (containing Strep and HA tags at C-terminal) using BamH1 and Not1 restriction sites to generate DVC1-WT-FRT/TO construct. The construct was used as a template to generate the E112A, Y117C and DC variants by site directed mutagenesis. The DVC1-wt and DVC1-mutation-FRT/TO constructs were transfected with pOG44 vector into Flip-In host cell lines for site-specific integration in genome. The transformed cells were selected under hygromycin B to generate DVC1-Flp-In T-REx stable cell lines. The expression of DVC1 was confirmed by doxycycline induction (1μg/ml) followed by western blot analysis.

Generation of Crispr/Cas9 SPRTN partial knock-out HeLa cells (Δ-SPRTN)

The CRISPR plasmid was obtained from the Genome Engineering Oxford (GEO) centre, Sir William Dunn School of Pathology, Oxford, UK. The donor plasmid (pX459v2), containing the sgRNA, Cas9 and puromycin resistance marker, was transfected into HeLa cells using Fugene HD (Promega). The sgRNA sequence (CACGCTCCACTTCACCTCGACGG) targeted the first exon of *SPRTN*. 24h after transfection, cells were selected with 0.6 μg/ml puromycin for 72h and then seeded as single cells in a 96-well plate (one cell/well) in order to generate a population of cells derived from a single clone (i.e. a genetically homogenous population of cells). In parallel, 40 distinct CRISPR-targeted HeLa cell clones (each grown from a single cell) were tested for SPRTN protein expression by Western blotting. Only those HeLa clones, which showed a reduction in SPRTN protein levels, were selected for sequencing. Genomic DNA was isolated from each individual pool of genetically-homogenous HeLa cells and the region containing the SPRTN sgRNA target site was amplified by PCR and subcloned into Topo vectors to achieve allele separation and transformed into bacteria. Plasmid was then isolated from 25 bacterial colonies and sequenced. The clones in Figure S1 refer to plasmids isolated from individual bacterial colonies derived from HeLa cells grown from a single clone. 70% (i.e. 17/25 or 2/3) of the sequences showed Cas9-mediated mutations in exon 1 of *SPRTN*. Knowing that HeLa cells (cancer cell line) have three copies of chromosome 1, where the *SPRTN* gene is located, this result suggests that 2 out of 3 alleles of the SPRTN gene have been knocked-out. Therefore we called our SPRTN-knock out cells "partial knock-out" as 2 alleles are knocked-out and 1 allele is still present.

Western Blot (WB)

Standard protocols for sodium dodecyl sulfate-polyacrylamide gel electrophoresis (SDS PAGE) and immunoblotting were used (Henderson and Wolf, 1992). Nitrocellulose membrane (GEHealthcare) or PVDF (BioRad) were used to transfer proteins from polyacrylamide gels depending on the antibody.

DNA-protein crosslinks isolation

DPCs were detected using a modified rapid approach to DNA adduct recovery (RADAR) assay (Kiianitsa and Maizels, 2013). In brief, 1.5 to 2 x 10^6 cells were lysed in 1 ml of M buffer (MB), containing 6 M GTC, 10 mM Tris–HCl (pH 6.8), 20 mM EDTA, 4% Triton X100, 1% Sarkosyl and 1% dithiothreitol. DNA was precipitated by adding 1 ml of 100% ethanol and was washed three times in wash buffer (20 mM Tris–HCl pH 6.8, 150 mM NaCl and 50% ethanol) and DNA was solubilized in 1 ml of 8 mM NaOH. A small aliquot of the recovered DNA was digested with 50 μg/ml proteinase K (Invitrogen) for 3 hours at 50°C and quantified using PicoGreen dye (Invitrogen) according to manufacturer instructions to determine DNA concentration. DNA concentration was further confirmed by slot-blot analysis followed by immunodetection with antibody against dsDNA.

As confirmation of results obtained by RADAR assay for DPC isolation, DPCs were isolated using

KCl/SDS precipitation assay (Zhitkovich and Costa, 1992). In brief, approximately $2x10^6$ cells were lysed in 1 ml denaturing lysis buffer (2 % SDS, 20 mM Tris/HCl pH 7.5) followed by sonication (5 cycles, 20 sec). Proteins were then precipitated in buffer containing 200 mM KCl, 20 mM Tris, pH 7.5 (assay buffer) and incubated on ice for 5 min. The precipitate was pelleted by centrifugation at 4°C (15,000g, 5min). Supernatant was used for quantifying soluble DNA. The pellet was resuspended in 1ml assay buffer and incubated at 55°C for 5 min, cooled on ice for 5 min, and precipitated by centrifugation (15,000g, 5min). Pellet was washed three times in assay buffer prior to final resuspension in 500 µl of assay buffer. Proteins were digested with 0.2 mg/ml Proteinase K (55°C, 3 hours). Samples were cooled on ice for 5 min then centrifuged. The final supernatant contained the crosslinked DNA. Soluble and crosslinked DNA were quantified by PicoGreen. The amount of DPCs was calculated as the ratio between DNA precipitated by SDS/KCl and total DNA (SDS/KCl precipitated plus soluble DNA).

DNA-protein crosslinks detection

Total DPCs were visualized by silver staining (Sigma) as recommended by the manufacturer after electrophoretic separation on polyacrylamide gels. DNA was digested with benzonase (Invitrogen) for 30 minutes at 37°C. Proteins were precipitated by standard Trichloroacetic Acid (TCA) protocol (Link and LaBaer, 2011) and resolved by SDS-PAGE gel. Specific DPCs were detected using a vacuum slot-blot manifold (Bio-Rad) followed by immunodetection. In brief, equal amounts of DNA were diluted in Tris-buffered saline (TBS) and applied to either a polyvinylidene difluoride (PVDF, Millipore) or nitrocellulose (Bio-Rad, Hercules CA) membrane using a vacuum slot-blot manifold. The membrane was then blocked in 3%BSA in TBST (TBS containing 0.1% Tween 20), incubated with primary antibodies followed by incubation with horseradish peroxidase-conjugated secondary antibodies. DPCs were visualized using the Bio-Rad ChemiDoc XRS Plus Analyzer. Sample loading was further confirmed by slot-blot detection of dsDNA with a specific antibody α-dsDNA. For the dsDNA detection samples were digested with proteinase K, diluted in Tris/Borate/EDTA (TBE) buffer, and applied to nylon membrane (Hybond N+).

Colony forming assay

Cell survival after exposure to CPT, Eto, FA or methylglyoxal was determined by standard clonogenic assay. In brief, cells were seeded in 6-well plates and incubated overnight. Cells were exposed to increase doses of the drugs diluted in DMEM, washed and incubated again in fresh medium. Cells were exposed for 24 hours with CPT and methylglyoxal, 1 hour with Eto and 20 minutes with FA. Colonies were fixed 7-10 days later, and the number of clones was counted using the automated colony counter GelCount™ (DTI-Biotech). The number of colonies in treated samples was expressed as a percentage of colony numbers in the untreated samples.

Cell viability assay

Cell viability assay was performed with resazurin dye according to the manufacturer's instructions (Cell signalling).

Cell cycle analysis

For flow cytometry analysis, 0.2 x 10^6 – 0.5 x 10^6 cells were harvested by centrifugation and fixed in ice-cold methanol, followed by resuspension in Phosphate-buffered saline (PBS) containing 1% Bovine serum albumin (BSA), 20 µg/ml of propidium iodide or 10 µg/ml DAPI and 10 µg/ml of RNase A. Flow cytometry was performed on a FACScalibur instrument (BD Biosciences). Cell-cycle phase distributions were analyzed using FlowJo software. Please describe here in brief or cite a paper for thymidine block and release.

Cell cycle synchronization

HeLa cells were synchronized at G1/S of the cell cycle by double thymidine treatment, as described previously(Harper, 2005). T24 cells were synchronized as described previously (Jin et al., 1997).

Protein purification

For overexpression in *E.coli* cells SPRTN constructs were cloned in to either the pNIC-ZB vector (full length SPRTN-WT, SPRTN-E112A, and SPRTN-Y117C), or pNIC28-Bsa4 vector (all other truncated constructs). For purification of full length constructs containing a TEV cleavable Z-basic-his tag, cell pellets were thawed and resuspended in lysis buffer (100 mM HEPES pH 7.5, 500 mM NaCl, 10% glycerol, 10 mM imidazole, 1 mM Tris (2-carboxyethyl) phosphene (TCEP), 0.1% DDM, 1 mM MgCl, 1 x set III protease inhibitors (Merck). Cells were lysed by sonication and 1 unit of Benzonase was added to lysates before the cell were debris pelleted by centrifugation. Lysates were applied to a Ni-sepharose IMAC gravity flow column, washed with 2 column volumes of wash buffer (50 mM HEPES pH 7.5, 500 mM NaCl, 10% glycerol, 45 mM imidazole, 1 mM TCEP), and eluted in elution buffer (50 mM HEPES pH 7.5, 500 mM NaCl, 10% glycerol, 300 mM imidazole, 1 mM TCEP). Elution fractions were applied directly to a 5ml Hitrap SP HP column (GE healthcare), washed with wash buffer (50 mM HEPES pH 7.5, 500 mM NaCl, 1 mM TCEP) and eluted with elution buffer (50 mM HEPES pH 7.5, 1M NaCl, 1 mM TCEP). The purification tag was cleaved with the addition of 1:20 mass ratio of His-tagged TEV protease during overnight dialysis into buffer A (20 mM HEPES, pH 7.5, 500 mM NaCl, 0.5 mM TCEP). Samples were concentrated by ultrafiltration using a 30 kDa molecular weight cut off centrifugal concentrator and loaded on to size exclusion chromatography using a HiLoad 16/60 Superdex 200 column at 1 ml/min in buffer A. The same protocol was used for purification of the truncated constructs with the exception of the omission of the SP HP column and the inclusion of a Ni-sepharose rebind following TEV cleavage (to remove his-

tagged TEV protease). Protein identities were verified by LC/ESI-TOF Mass spectrometry and protein concentrations were determined by absorbance at 280nm (Nanodrop) using the calculated molecular mass and extinction coefficients.

Fluorescence polarisation DNA binding assays and DNA probe annealing

DNA binding was measured using a fluorescence polarisation based assay. DNA oligonucleotides with the sequences as follows

OD1 - ATC GAT AGT CGG ATC CTC TAG ACA GCT CCA TGT AGC AAG GCA CTG GTA GAA TTC GGC AGC GTC,

OD2 - GAC GCT GCC GAA TTC TAC CAG TGC CTT GCT ACA TGG AGC TGT CTA GAG GAT CCG ACT ATC GAT, and

OD3 - GAC GCT GCC GAA TTC TAC CAG TGC CTT GCT AGG ACA TCT TTG CCC ACC TGC AGG TTC ACC C were mixed together at a 10 µM concentration in a buffer consisting of 10 mM HEPES pH 7.5, 50 mM NaCl in the following combinations, single stranded (ss) =OD1, double stranded (ds) OD1+OD2 and splayed duplex OD1+OD3. For all substrates the OD1 oligo was labelled on the 5' end with Fluorescein isothiocyanate and substrates were formed by heating 96°C and allowing to cool on a heat block over 2 hrs. Probes were used at a final concentration of 10 nM and binding experiments were performed in a buffer containing 10 mM HEPES pH 7.5, 150 mM NaCl, with varying protein concentration. Measurements were performed in 384 well plates (30 µl final volume) in a POLARstar plate reader (BMG Labtech) with excitation at 485 nm and emission at 520 nm. Kinetic constants were calculated from binding curves using a 4 parameter logarithmic binding equation using the program PRISM (GraphPad).

***In vitro* self –cleavage assays**

SPRTN self-cleavage assays were performed in 20 µl reaction volume, containing 2 µl SPRTN (1 mg/ml), 10 µl of reaction buffer (25 mM Tris pH 7.5, 150 mM NaCl), 6 µl H2O, and 1 µl DNA (10 µM). Several types of DNA probes were used for induction of cleavage, ssDNA and dsDNA of various lengths: 100, 32, 16, and 8 bp. Samples were incubated for 2 hours at 37°C, and stopped by the addition of 2X Laemmli buffer and resolved by SDS-PAGE followed by coomassie blue staining.

***In vitro* cleavage of the SPRTN substrates**

SPRTN enzymatic reactions with histones were performed in 150 mM NaCl, 25 mM Tris pH 7.4 in a PCR block at 37°C. The reaction volume was typically 10 µl and contained: E. coli purified recombinant SPRTN (1 – 10 mg/ml solution), substrate (typically 1 mg/ml solution) and 100 bp long dsDNA oligonucleotide probe (**OD4** ACGCGGGTTAGCGGTACCCAGTCCAGTGACCTAGGCAGCTTTAAGCTAGTACGACTTGCT TAGATTGCAGTCGACGACGTAGCTGGCATAGAGGTACAGC) (40 µM stock solution) in

Klenow buffer without DTT. Conditions for cleavage reactions of histones were performed with the 3:1 molar ratio of SPRTN:substrate and 15:1 molar ratio of SPRTN: dsDNA in a 10μl volume. The cleavage reactions for Topo1 and Topo2α were performed in 16 μl volume and 30:1 molar ratio of SPRTN : dsDNA. Reactions were incubated for 20h at 37˚C and stopped by the addition of 2X Laemmli buffer. Reactions were resolved by SDS-PAGE followed by western blotting with substrate specific antibody. The enzyme (SPRTN) loading was monitored with α-SPRTN (N-terminal) in each reaction mixture.

***In vitro* DPCs cleavage**

DPCs were prepared according to the SDS/KCl precipitation assasy with the following modifications. Samples were washed in buffer containing 200 mM KCl, 20 mM Tris (pH 7.5), and DPCs were separated from total proteins using the QIAquick PCR Purification Kit (Qiagen). I*n vitro* DPC cleavage was performed in 150 mM NaCl, 25 mM Tris pH 7.4 in a PCR block at 37°C for 3 hours. The reaction volume was typically 20 μl and contained 10 μg of DPCs and 1 μg of recombinant SPRTN protein. Reactions were stopped by the addition of 2X Laemmli buffer, resolved by SDS-PAGE and visualized by silver staining.

Immunoprecipitation

To isolate Topo1 and Topo2α, HEK293T cells were transiently transfected with GFP-Topo2α or YFP-Topo1 using PEI (polyethyleneimine) reagent (Tom et al., 2008). Both proteins were immunoprecipitated using GFP-trap beads (Chromotek) in denaturing conditions (1% SDS, 5mM EDTA). Cell lysates were digested overnight with benzonase nuclease (Sigma) to further solubilize the sample. Sonication was avoided due to the fact that it disrupts binding of YFP-Topo1 or GFP-Topo2α to the GFP beads. Samples were diluted ten times in the IP buffer (150 mM NaCl, 10 mM Tris, 0.5 mM EDTA pH 7.5) with 1% Triton and precleared with bab-20 beads (Chromotek) for 30 min at 4°C. After discarding the bab-20 beads (50 G, 30 sec centrifugation), precleared samples were incubated with GFP beads (Chromotek) for 4h at 4°C, followed by five washes in the IP buffer. Cells were treated with 10 μM CPT or 25 μM ETO for 1h, when indicated.

Co-immunoprecipitation

To isolate SPRTN-interacting proteins, lysates from Flp-In 293 cells upon doxycycline induction of SPRTN-WT-SSH (Strep-Strep-HA tag) were prepared. Samples were lysed in 1% Triton, 150 mM NaCl, 50 mM Tris, 1mM EDTA pH 7.4 for 1h at 4°C with 10 mM NEM and protease and phosphatase inhibitor cocktails (ThermoFisher Scientific) and avidin (IBA), followed by centrifugation at 16,000 g for 10 min. The pellet was digested with nuclease benzonase in the bensonase buffer (Invitrogen) including protease and phosphatase inhibitor cocktail, overnight at 4°C. Both fractions were pooled and diluted 10 times in IP buffer (150 mM NaCl, 50 mM Tris, pH 8)

followed by preclearing of the lysate with blank sepharose (IBA) for 1h at 4°C. Samples were incubated with Strep-Tactin sepharose (IBA) for 2h at 4°C, washed 5 times in IP buffer containing 0.05% NP-40 and eluted in 2X Laemmli for 10 min at 95°C.

Cellular fractionation

Cellular fractionation was performed as previously described (Mendez and Stillman, 2000) with the slight modifications as follows: after isolation of cytosolic and nuclear soluble fractions, the chromatin fraction was digested with benzonase 1µl of benzonase (200u/ml) in a buffer containing 50 mM Tris, pH 7.9, 50 mM NaCl and 5 mM KCl overnight at 4˚C with gentle agitation.

Isolation of Proteins On Nascent DNA (iPOND)

iPOND was performed as described in (Sirbu et al., 2012) with the following modifications. Newly synthesized DNA in HEK293 or HeLa wild-type or SPRTN-knockout cells (~2 x 10^8 cells per condition) was labelled via incubation with 10 µM Edu for 10 and 15 minutes, respectively. For thymidine chases, cell culture media was supplemented with 10 µM thymidine and incubated for 2 or 10 minutes as indicated. HEK293 cells were synchronised by double thymidine block, and washed thoroughly with 1 x PBS followed by cell culture media and released into S-phase for 4 hours before EdU labelling. For experiments in HEK293 cells, chromatin was fragmented into 50-300 bp fragments by sonication with a Bioruptor Plus sonicator (30 seconds ON, 30 seconds OFF for 50 cycles). In HeLa cells, chromatin was fragmented into 400 bp fragments (30 seconds ON, 30 seconds OFF for 5 cycles). To isolate proteins on Edu-labelled DNA, samples from incubated overnight with streptavidin-coupled agarose beads (Merck Millipore). Aliquots of each extract were kept for loading controls.

DNA fiber assay

The DNA fiber assay was performed as described previously (Lessel et al., 2014). Briefly, asynchronous LCL or HeLa cells were labelled with 30 µM of CldU (Sigma, C6891) for 30 min, and then labelled with 250 µM of IdU (Sigma, 17125) for an additional 30 min. HeLa cells were washed 3 times with warm PBS after 1st nucleotide (CldU) incubation. DNA replication was inhibited by treating cells with ice-cold PBS. Cells were lysed in 200 mM Tris-HCl pH 7.4, 50 mM EDTA and 0.5% SDS, DNA fibers were spread onto glass slides, fixed with 3:1 methanol and acetic acid, denatured with 2.5 M HCl, blocked with 2% BSA and stained with anti-rat and anti-mouse 5-bromo-2′-deoxyuridine (BrdU) that specifically recognize either CldU (Abcam-Ab6326, dilution 1:500) or IdU (BD- 347580, dilution 1:100). Anti-rat Cy3 (dilution 1:300, Jackson Immuno Research, 712-116-153) and anti-mouse Alexa-488 (dilution 1:300, Molecular Probes, A11001) were used as the respective secondary antibodies. Microscopy was done using a Leica DMRB microscope with a

DFC360FX camera. The lengths of the CldU- and IdU-labelled tracts were measured by ImageJ software and converted into micron scale. Statistical analysis was done by GraphPad Prism software using unpaired *t*-test. Mean value was then converted into kilo base (1µm = 2.59 kb) to precisely determine the replication fork speed as shown previously (Petermann et al., 2010). For the DNA fiber assay under genotoxic stress, the second nucleotide (IdU) was either mock treated or incubated in the presence of 25 nM CPT or 50 µM formaldehyde (30 min).

Mass spectrometry

Mass Spectrometry of Intact Proteins

For mass spectrometry under denaturing conditions 50-µl protein samples were injected at ~0.02 mg/ml in 0.1% formic acid onto a 2.1 mm x 12.5 mm Zorbax 5 µm 300SB-C3 guard column (Agilent) resolved by reversed-phase chromatography at 40 °C. The solvent system was 0.1% formic acid in LC-MS grade water (buffer A) and 0.1% formic acid in LC-MS grade methanol (buffer B), and proteins were eluted with a linear gradient of 5–95% buffer B over 1.5 min at 1.0 ml/min. Protein masses were determined using an Agilent 6530 QTOF. The analysis of MS spectra was performed in ICP-MS MassHunter Software (Agilent). Analysis of histone and SPRTN peptides was done using Protein Analysis Worksheet (PAWS). Only cleavage products (CPs) which could be determined with high confidence (unique peptide matches, ppm 50) are taken into consideration for the analysis of SPRTN cleavage site. Histone protein sequences H2A (AAN59960.1), H2B (AAN59961.1), H3 (NP_002098.1) and H4 (NP_778224) without initial Methionine residue corresponded to the recombinant protein molar mass as stated by the manufacturer (NEB). For mass spectrometry under native conditions protein samples, 50 µl at 1 mg/ml were buffer exchanged into a buffer consisting of 50 mM ammonium acetate pH 6.5, and injected directly at a flow rate of 6 µl per minute. Protein masses were determined using an Agilent 6530 QTOF.

Mass spectrometry for the identification of proteins crosslinked to the DNA

DPCs were isolated as described previously. Equal amounts of DNA were digested with benzonase (Invitrogen) for 60 minutes at 37°C. Proteins were precipitated by standard Trichloroacetic Acid (TCA) protocol and ressuspend in 6M urea solution. Proteins were then reduced with dithiothreitol (5 mM) alkylated with iodoacetamide (20 mM) and precipitated again via standard methanol/chloroform extraction. Proteins were resuspended in 50 ml 6M urea and urea was adjusted to a final concentration of 1 M. Samples were digested at 37 °C ON with trypsin. Peptides were purified using SEP-C18 purification columns and peptides dry using speed vac. Dried tryptic peptides were reconstituted in 15µl of LC-MS grade water containing 2% acetonitrile and 0.1% trifluoroacetic acid. Samples were subsequently analysed by nano-liquid chromatography tandem mass spectrometry (LC-MS/MS) using a Dionex Ultimate 3000 UPLC coupled to a Q Exactive hybrid quadrupole orbitrap mass spectrometer (ThermoFisher Scientific) as described previously (Chung et al., 2016; Lochmatter et al., 2016; Michalski et al., 2011). Briefly, peptides were desalted on a PepMapC100 column (100µm x 20mm,

5µm particle size, ThermoFisher Scientific) for 3minute at a flow rate of 8 µl/min and separated on a directly coupled nEASY column (PepMap C18, 75 µm x 500mm, 2 µm particle, ThermoFisher Scientific) using a multistep gradient starting with 3 min at 2% Acetonitrile in 5% DMSO with 0.1% Formic acid (buffer B), followed by 60 min linear gradient up to 35% buffer B at 250nl/min flow rate, 7 min linear gradient up to 99% buffer B and maintained at 99% buffer B for 5 min at a flow rate of 250nl/min before reverting to 2% buffer B for 3 min prior to a last 4 min at 99% solvent B. Full MS scans were acquired over the m/z range of 380 - 1800 at a resolution of 70,000 at 200m/z (AGC target of $3x10^6$ ions). MS/MS data was acquired in a data dependent manner by selecting the 15 most abundant precursor ions for HCD fragmentation (CE of 28) and on MS/MS resolution of 17,500.

Quantitative proteomics and data analysis

Label-free quantitation was used to identify overexpressed proteins in SPRTN depleted cells. Raw LC-MS/MS data was uploaded into Progenesis QI Proteomics v2.0 software (Waters). After sample runs alignment, filtering, peak detection and quantification/normalisation (using default parameters), a peak list containing all peptide precursor ions detected across all experimental conditions and biological replicates was generated and exported as mgf file. Peptide and protein identifications was performed using MASCOT v2.5. Data was searched against the Human UniProt SwissProt database (20,268 Homo sapiens sequences; retrieved 20151126) using the Decoy function, whilst selecting trypsin as enzyme (allowing 1 miscleavages), peptide charge of +2, +3, +4 ions, peptide tolerance of 10 ppm and MS/MS of 0.05 Da; $^{\#13}C$ at 1; Carboamidomethylation as fixed modification, and Oxidation (M) and Deamidation (N and Q) as a variable modification. MASCOT data search results were filtered using ion score cut off at 20 and a false discovery rate (FDR) of 1%. Filtered data was imported into Progenesis for subsequent revision of normalization and statistical analysis. Only proteins identified with at least two peptides (peptide counts) and quantified with at least two peptides (unique peptides) were considered for statistical analysis. Differentially expressed proteins and in particular proteins overexpressed in SPRTN depleted cells were selected by p-Anova $p<0.05$ and fold change. The gene ontology tool (PANTHER; Protein ANalysis THrough Evolutionary relationships, version 10.) was used to determine the protein function class of the proteins significantly overexpressed in SPRTN depleted cells.

Statistical analysis

Statistical analysis was done by GraphPad Prism software using unpaired *t*-test or as otherwise described under individual methods subheadings. p value = ns; non-significant, $*p < 0.05$, $**p < 0.01$ and $***p < 0.001$.

In silico methods

Disordered protein regions were predicited using PONDR-FIT (Xue et al., 2010).

DNA binding residues were predicted using MetaDBsite (Si et al., 2011).

SUPPLEMENTARY REFERENCES

Chung, V.Y., Konietzny, R., Charles, P., Kessler, B., Fischer, R., and Turney, B.W. (2016). Proteomic changes in response to crystal formation in Drosophila Malpighian tubules. Fly (Austin) *10*, 91-100.

Harper, J.V. (2005). Synchronization of cell populations in G1/S and G2/M phases of the cell cycle. Methods Mol Biol *296*, 157-166.

Henderson, C.J., and Wolf, C.R. (1992). Immunodetection of proteins by Western blotting. Methods Mol Biol *10*, 221-233.

Jin, Y., Xu, X.L., Yang, M.C., Wei, F., Ayi, T.C., Bowcock, A.M., and Baer, R. (1997). Cell cycle-dependent colocalization of BARD1 and BRCA1 proteins in discrete nuclear domains. Proc Natl Acad Sci U S A *94*, 12075-12080.

Kiianitsa, K., and Maizels, N. (2013). A rapid and sensitive assay for DNA-protein covalent complexes in living cells. Nucleic acids research *41*, e104.

Lessel, D., Vaz, B., Halder, S., Lockhart, P.J., Marinovic-Terzic, I., Lopez-Mosqueda, J., Philipp, M., Sim, J.C., Smith, K.R., Oehler, J., *et al.* (2014). Mutations in SPRTN cause early onset hepatocellular carcinoma, genomic instability and progeroid features. Nature genetics *46*, 1239-1244.

Link, A.J., and LaBaer, J. (2011). Trichloroacetic acid (TCA) precipitation of proteins. Cold Spring Harb Protoc *2011*, 993-994.

Lochmatter, C., Fischer, R., Charles, P.D., Yu, Z., Powrie, F., and Kessler, B.M. (2016). Integrative Phosphoproteomics Links IL-23R Signaling with Metabolic Adaptation in Lymphocytes. Sci Rep *6*, 24491.

Mendez, J., and Stillman, B. (2000). Chromatin association of human origin recognition complex, cdc6, and minichromosome maintenance proteins during the cell cycle: assembly of prereplication complexes in late mitosis. Mol Cell Biol *20*, 8602-8612.

Michalski, A., Damoc, E., Hauschild, J.P., Lange, O., Wieghaus, A., Makarov, A., Nagaraj, N., Cox, J., Mann, M., and Horning, S. (2011). Mass spectrometry-based proteomics using Q Exactive, a high-performance benchtop quadrupole Orbitrap mass spectrometer. Mol Cell Proteomics *10*, M111 011015.

Petermann, E., Woodcock, M., and Helleday, T. (2010). Chk1 promotes replication fork progression by controlling replication initiation. Proc Natl Acad Sci U S A *107*, 16090-16095.

Si, J., Zhang, Z., Lin, B., Schroeder, M., and Huang, B. (2011). MetaDBSite: a meta approach to improve protein DNA-binding sites prediction. BMC Syst Biol *5 Suppl 1*, S7.

Sirbu, B.M., Couch, F.B., and Cortez, D. (2012). Monitoring the spatiotemporal dynamics of proteins at replication forks and in assembled chromatin using isolation of proteins on nascent DNA. Nat Protoc *7*, 594-605.

Tom, R., Bisson, L., and Durocher, Y. (2008). Transfection of Adherent HEK293-EBNA1 Cells in a Six-Well Plate with Branched PEI for Production of Recombinant Proteins. CSH Protoc *2008*, pdb prot4978.

Xue, B., Dunbrack, R.L., Williams, R.W., Dunker, A.K., and Uversky, V.N. (2010). PONDR-FIT: a meta-predictor of intrinsically disordered amino acids. Biochim Biophys Acta *1804*, 996-1010.

Zhitkovich, A., and Costa, M. (1992). A simple, sensitive assay to detect DNA-protein crosslinks in intact cells and in vivo. Carcinogenesis *13*, 1485-1489.
